# Losing identity: structural diversity of transposable elements belonging to different classes in the genome of *Anopheles gambiae*

**DOI:** 10.1186/1471-2164-13-272

**Published:** 2012-06-22

**Authors:** Rita D Fernández-Medina, José M C Ribeiro, Claudia M A Carareto, Luciane Velasque, Cláudio J Struchiner

**Affiliations:** 1 Escola Nacional de Saúde Pública Sergio Arouca, Fundação Oswaldo Cruz, Rio de Janeiro, Brazil; 2 Section of Vector Biology, Laboratory of Malaria and Vector Research, National Institute of Allergy and Infectious Diseases, National Institutes of Health, Rockville, MD, 20852, USA; 3 Laboratório de Evolução Molecular, Departamento de Biologia, UNESP, Universidade Estadual Paulista, 15054-000, São José do Rio Preto, SP, Brazil; 4 Departamento de Matemática e Estatística, Universidade Federal do Estado do Rio de Janeiro, Rio de Janeiro, Brazil; 5 Instituto de Medicina Social/Universidade do Estado do Rio de Janeiro, Rio de Janeiro, Brazil

**Keywords:** Transposable elements, LTR, Non-LTR, Class II, Deterioration, *Anopheles gambiae*

## Abstract

**Background:**

Transposable elements (TEs), both DNA transposons and retrotransposons, are genetic elements with the main characteristic of being able to mobilize and amplify their own representation within genomes, utilizing different mechanisms of transposition. An almost universal feature of TEs in eukaryotic genomes is their inability to transpose by themselves, mainly as the result of sequence degeneration (by either mutations or deletions). Most of the elements are thus either inactive or non-autonomous. Considering that the bulk of some eukaryotic genomes derive from TEs, they have been conceived as “TE graveyards.” It has been shown that once an element has been inactivated, it progressively accumulates mutations and deletions at neutral rates until completely losing its identity or being lost from the host genome; however, it has also been shown that these “neutral sequences” might serve as raw material for domestication by host genomes.

**Results:**

We have analyzed the sequence structural variations, nucleotide divergence, and pattern of insertions and deletions of several superfamilies of TEs belonging to both class I (long terminal repeats [LTRs] and non-LTRs [NLTRs]) and II in the genome of *Anopheles gambiae*, aiming at describing the landscape of deterioration of these elements in this particular genome. Our results describe a great diversity in patterns of deterioration, indicating lineage-specific differences including the presence of Solo-LTRs in the LTR lineage, 5′-deleted NLTRs, and several non-autonomous and MITEs in the class II families. Interestingly, we found fragments of NLTRs corresponding to the RT domain, which preserves high identity among them, suggesting a possible remaining genomic role for these domains.

**Conclusions:**

We show here that the TEs in the *An. gambiae* genome deteriorate in different ways according to the class to which they belong. This diversity certainly has implications not only at the host genomic level but also at the amplification dynamic and evolution of the TE families themselves.

## Background

Transposable elements (TEs) are genetic elements that share the main characteristic of amplifying their own representation within genomes. Due to their ability to spread in the absence of selection at the host level, they persist in genomes even at the expense of a net negative fitness to the hosts [1]. Under this view, these elements are conceived as genomic parasites. Today, the view of TEs within genomes has changed considerably, and these elements have been shown to be major contributors to both genome evolution and function [2-5]. The relationships between TEs and host genomes where they reside are now considered a continuum from parasitic to beneficial to genomes [6]. TEs show very different genetic structures and transposition strategies and accordingly have been classified into two classes (I and II) and subsequently into orders, superfamilies, and families [7]. Class I elements— also called retrotransposons, which depend on a RNA intermediary for their replication—are further classified into two orders: LTRs (which harbor flanking long terminal repeats [LTRs]) and non-LTRs (NLTRs), which share the transposition mechanism known as “copy and paste.” Both types of elements are first transcribed into mRNA, but LTRs are then retrotranscribed into a DNA copy that is later inserted into the genome, while NLTRs are retrotranscribed at the time they are inserted into the genome. Both processes lead to generation of novel copies. Class II—also called DNA transposons—present a simpler genetic structure that includes a gene encoding for the enzyme transposase (responsible for the element transposition) and flanking terminal inverted repeats (TIRs), which are recognition sites for transposase. Their transposition mechanism is known as “cut and paste” because they are usually excised from one strand at its original place and inserted into a new genomic position. Increasing copy number depends on strand repair mechanisms from homologous chromatids that are dependent on host enzymes. These intrinsic differences imply differences in the way elements belonging to the different classes and orders amplify and degenerate within genomes.

A TE family “life cycle” has been conceived as the successive steps from its “birth” in a given genome until its “death”, and includes the invasion, amplification, inactivation, and elimination from a genome that can occur in geological times. It has been shown that once an element has been inactivated, it progressively accumulates mutations and indels at neutral rates, until completely losing its identity or being lost from the host genome. It has also been shown that these deteriorated elements behave in the genome as “neutral sequences” and might serve as raw material for domestication by host genomes. The concept of “molecular domestication” was used to describe a process whereby a TE sequence is co-opted to perform a different role from the original function that it was selected for and that benefits the host genome [8]. Indeed, TE truncated copies can modulate host gene expression by providing new regulatory sequences, alternative splice sites, polyadenylation signals [9], and new transcription factor binding sites [10], as well as in post transcriptional regulation through RNA editing and translation regulation (reviewed in [11]). In addition, it has been shown that several microRNA genes derive from TEs [12].

An almost universal feature of TEs now in eukaryotic genomes is their inability to transpose by themselves, mainly as the result of sequence degradation although even TEs that have lost their functional transposition machinery can continue to be mobilized by other intact element products. For example, a class II element that has mutated its transposase but not its inverted repeats can be mobilized by the intact transposase of another element of the same family. Accordingly, elements can also be classified as autonomous and non-autonomous according to the nature of their mobilization. TEs thus are also genomically interesting because they can persist and live in the genome as dead elements [13] (after inactivation by mutations, indels, or recombination), and these “fossil sequences” will continue to evolve in the genome, leaving traces of their past history. The relationship between TEs belonging to different families and superfamilies has been recently analyzed as the relationship between different populations and species in community ecology [14,15]. Within this scope, autonomous and non-autonomous elements of the same family are considered as competitors. TEs are simultaneously part of the genome and independent entities living their own life within the genome [16]. TE dynamics can be analyzed both at the intra-genomic level (the different TE families considered as different populations in ecology) and at the intra-population level (considering the complex networks of interactions between autonomous and non-autonomous elements, besides the relationships between different families of elements residing in the same genome). This view is of particular interest when asking about the relative abundances and degrees of activity or level of degradation of TEs in a given genome. Then, in genomes where several TE families coexist, the amplification of one family could have an impact on the dynamics of other families [16]. Frequencies, intragenomic dynamics, distributions, and abundances of retrotransposons and transposons differ considerably between different species.

The description of the “deterioration landscape” of the TE families in a given genome might shed light on the distribution and abundance of the TE families coexisting in a genome. Although the various sequencing projects have allowed the study of the TE content in different genomes, the aspects related to deteriorated elements have been barely studied [17]. Polymorphisms seen at the nucleotide level of sequences belonging to the same family can give information regarding the way that TEs are transposed and regulated as well as how they degenerate (by insertions, deletions, substitutions, or rearrangements).

Differences in these aspects could indicate the existence of regulatory mechanisms acting in a class-specific way. In fact, self-regulation [18], which has been shown to depend on the TE family, regulation by mutant copies, and genomic regulation [19,20] are mechanisms that have all been described in several TE families. On the other hand, host genomes have evolved mechanisms to control or restrict TE replication. Chromatin remodeling [21], methylation of TE DNA sequences [22], and non-coding small RNAs [23,24] or cytidine deaminases [25] are examples of these mechanisms (wee [26] for a review). We have previously analyzed the TE families present in AnoTExcel, an online *Anopheles gambiae* TE-specific database [27]. In that database, we presented the general features of the TE landscape in the malaria mosquito genome. AnoTExcel presents all the individual sequences belonging to different families in the mosquito genome, which allows analysis of the dynamics and demographic trajectory of certain TE families [28]. Now, to better characterize the *An. gambiae* mobilome, we have analyzed the sequence structural variations, focusing specifically on the TE deteriorated landscape of elements belonging to classes I and II. We describe a diversity of patterns of deterioration, indicating lineage-specific differences including the presence of Solo-LTRs, 5′-deleted NLTRs, and several non-autonomous and MITEs belonging to class II families.

## Results and discussion

Although different software tools have been used [29-36] for successful identification and/or characterization of repeats in eukaryotic genomes, so far there is no universal TE detector tool, and most softwares are biased toward specific questions (i.e., masking of repeats or identification of a certain class or family of TE by genome blasting). On the other hand, there is no universal database for finding detailed information on the TE families present in a given genome. Repbase is a fairly complete collection of repetitive elements in several eukaryotic genomes [37]; however, it only presents consensus sequences, and the individual sequences used to generate those consensuses are not available. Consequently, there is a lack of reports dealing with the general features of the whole repertoire of TEs in certain genomes, even if there are excellent exceptions as in Lerat et al. 2003 [17]. It is due to this lack of information that we are missing a complete picture of the characteristics of the TE families in *An. gambiae*, especially in relation to the sequence diversity within given families.

In an attempt to describe the diversity of elements and their deteriorated patterns, we have first compiled all the TEs so far described in the *An. gambiae* genome and used several families of elements from AnoTExcel—a previously characterized database [38] presenting detailed information on the TEs in *An. gambiae*[27]—to characterize their degree and patterns of deterioration.

### TE in *An. gambiae*

**Figure 1:**
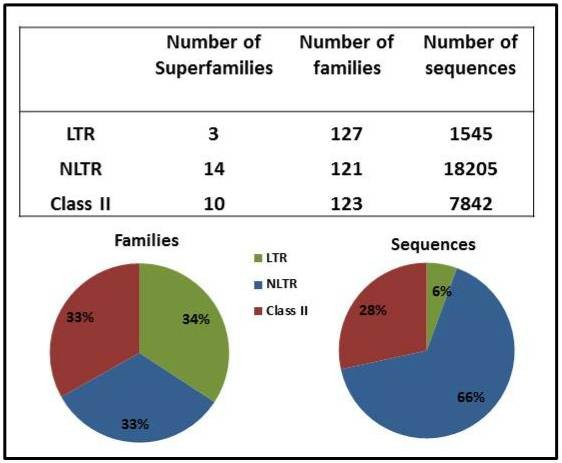
**Distribution of transposable elements in the genome of *Anopheles gambiae* .** Data were compiled from Repbase (RB), Tefam (TF), and a series of publications describing transposable elements in the mosquito genome.

**Figure 2:**
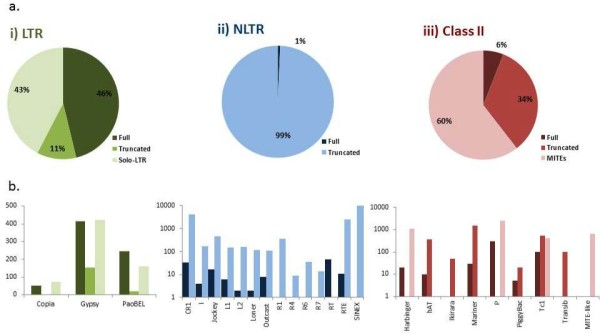
**A) Proportion of full-length, truncated, Solo-LTR, and MITE-like sequences for i) LTRs, ii) non-LTRs and iii) class II elements in the *An. gambiae* genome.B**) Copy number of full-length, truncated, Solo-LTR, and MITE-like sequences for each superfamily analyzed. Data were collected from Repbase, Tefam, and AnoTExcel. Obs: the y-axis for Figure b ii and iii are logarithmic.

The TEs in the *An. gambiae* genome are represented by members of both classes (I and II) with approximately 25 different superfamilies, each composed of several families (127, 121, and 123 for the LTRs, NLTRs, and Class II, respectively) (Figures 1 and 2). They constitute between 12% and 16% of the mosquito genome [39,40]. According to the preliminary genomic analysis [40], LTR elements were the most abundant TEs, followed by SINEs (non-autonomous NLTRs) and MITEs (non-autonomous class II elements) [40]. We have now scrutinized all the TE elements deposited in Repbase [37], TEfam [41], and the publications reporting TEs in *An. gambiae* so far [27,42-51]. In accordance with previous publications [39,52], we found that NLTRs are the most abundant type of elements in this organism, in terms of both diversity (number of superfamilies) and abundance (number of individual sequences), followed by elements belonging to class II ( Additional file 1: Table S1 and Figure 1); however, the proportion of full-length and truncated elements varies considerably for each class. The former corresponds to 46, 1, and 6% for LTR, NLTR, and class II, respectively (Figure 2). The higher proportion of full-length elements in the Class I, LTR order in comparison to NLTRs and Class II could have important consequences in the dynamic of this particular genome. The activity of LTR elements could have an impact on expression of mosquito genomes, but in addition, these elements could produce important structural consequences through recombination among their LTRs; however, a great part of the LTR elements here analyzed correspond to Solo-LTRs (43%; Figure 2a). Within the LTR order, the Gypsy superfamily has the majority of the families and the higher diversity of elements (Figure 2b) [43]. On the other hand, there are only ten Copia superfamilies in the mosquito genome, and they contain a similar number of full-length and Solo-LTR sequences. The Copia families are composed of very few sequences presenting high degrees of nucleotide identity, indicating that they were active until recently. The Pao-Bel elements have an intermediate number of families and are mainly represented by full-length sequences. As a whole, the LTR elements present a low proportion of fragmented sequences together with a high proportion of Solo LTRs; this seems to indicate that the main mechanism driving the deterioration of LTR elements is through the generation of Solo elements, probably driven by LTR recombination as it was previously described for the Ty3/Gypsy elements [52].

On the other hand, NLTRs form a diverse group with all the families presenting heavily deteriorated sequences constituting up to 99% of this order, with some superfamilies presenting only truncated sequences (Figure 2a,b). The retrotransposition mechanism of NLTRs commonly involves generation of 5′ truncated sequences [53-55] by a mechanism that is not clearly understood. These truncated sequences are integrated in the genome and are believed to evolve neutrally [54] and have been designated “dead-on-arrival” (DOA). The mechanism for generation of 5′-truncated sequences seems not to be universal for all NLTR superfamilies, as they are not present in all the species where NLTRs have been described. A great part of the total amount of NLTR elements in this genome is due to the presence of thousands of SINE elements belonging to the SINEX-1 family [56].

The class II elements are also heavily deteriorated (94% correspond to truncated sequences or MITEs) (Figure 2a,b). A great part of them belongs to MITEs (60%), or non-autonomous elements, with several internal deletions and with no coding capacity but able to be mobilized by the action of active elements in *trans*. This means that the truncated class II elements are still participating—or at least are able to do so—in the transposition dynamics of the families that they belong to.

### Deterioration of TEs in *An. gambiae*

TEs are expected to diverge from their original sequence both in nucleotide composition and in structure during their genomic evolution; however, elements belonging to different classes and orders incorporate errors at different rates and in different ways according to their own mechanisms of replication and to the enzymes involved in their replication. These errors would be lost or fixed, leaving a different landscape of deterioration of the several TE families in different genomes.

The TE content of a genome contributes significantly to differences in the amount of genomic DNA between phylogenetically close species. For instance, the TE content of *Aedes aegyptiCulex quinquefasciatus,* and *An. gambiae* differs significantly with DNA genomic size [39]. On the other hand, the great part of the TEs identified in today’s genomes, including *An. gambiae,* corresponds to deteriorated sequences or remnants of once active elements [57]. It has been previously described [35] that the TEs in *Drosophila melanogaster* and *An. gambiae* are, on average, 12 and 24% of the length of their full-length counterparts, respectively, indicating that these elements suffer an important degree of deterioration through deletions in their life cycle. The deterioration process of TEs drives their sequences to a loss of identity, which can be guided by the accumulation of deleterious mutations, incorporation of indels generating frame shifts, or deletion of longer regions in their coding sequences or recognition sites (segmental deletions).

TEs belonging to different classes are known to deteriorate in different ways, in accordance with their different structures and mechanisms of amplification. Class I LTR elements form Solo-LTRs as a by-product of recombination between flanking LTRs of the same element, which is actually believed to be the main force driving the deterioration process of these elements [52,58,59]. The other elements belonging to this class, the NLTRs, have a completely different mechanism of transposition involving a target site-primed reverse transcription (TPRT), which is believed to be related to generation of DOA elements, i.e., sequences lacking different sizes of their 5′ ends [60,61]. Nevertheless, generation and maintenance of 5′-deleted NLTR sequences are not understood completely, and it might be possible that other mechanisms are involved—for instance, previous deterioration of the sequences that are lost during replication, or the presence of common motifs that might serve as templates for (endo) nucleases. Last, class II elements tend to incorporate nucleotide substitutions and internal deletions, which in turn are believed to be related to the formation of MITEs, which are small, non-autonomous class II elements that maintain their terminal inverted repeats, and amplify by using the transposases of active elements *in trans*.

Here, in an effort to understand the patterns of deterioration of TEs in the genome of *An. gambiae*, we have analyzed several TE families belonging to the main superfamilies in the genome. We obtained nucleotide sequences belonging to different TE families from AnoTExcel [27]. The family sequences were further aligned to reference sequences from Repbase or TEfam and analyzed by the EMBOSS program Plotcon, which gives a graphic representation of the point by point similarities of sequences in family alignments. This qualitative analysis was complemented by the estimates of the p-distances among different regions of the full-length elements as well as the deteriorated sequences in each family, to present a picture of the main differences in the deterioration process followed by different elements.

### Class I, LTR elements

**Figure 3:**
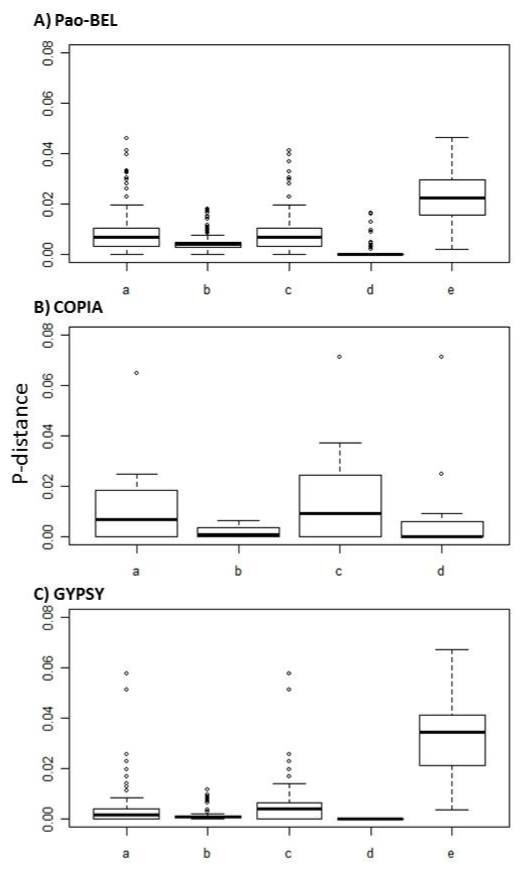
**Number of base substitutions per site (p-distance) from between sequences for the three LTR superfamilies in *Anopheles gambiae* .** Analyses were conducted using the Kimura 2-parameter model. Rate variation among sites was modeled with a gamma distribution (shape parameter = 1). All ambiguous positions were removed for each sequence pair. a) 5′-LTR (population comparison); b) INTERNAL REGION (population comparison); c) 3′-LTR (population comparison); d) 5′ to 3′-LTR comparison within each full-length sequence within the population; and e) SOLO-LTRs (only for Pao-BELs and Gypsy superfamilies). **A**) Pao-Bel superfamily (12 full-length and 3 Solo-LTR families). Kruskal-Wallis test: p value < 0.001. Ad hoc, Tukey non-parametric test: all comparisons were significant at p < 0.05 except a–c. **B**) Copia superfamily (four families). Kruskal-Wallis test: p-value = ns. Not significant differences among the regions and (**C**) Gypsy superfamily (six full-length and eight Solo-LTR families). Kruskal-Wallis test: p-value < 0.001. Ad hoc, Tukey non-parametric test: all comparisons were significant at p < 0.05, except a–b, a–c, and b–c with p-value > 0.05.

We analyzed the full-length and Solo-LTR elements of several families belonging to the three LTR superfamilies: Pao-Bel, Copia, and Gypsy. The alignments corresponding to full-length sequences were divided into three regions—the 5′ LTR, the internal region, and the 3′ LTR—and p-distances were calculated for each of the three regions. To evaluate the relative age of the LTR elements, we also calculated the p-distances between the 5′ and 3′ LTR for each individual sequence in the alignment. The p-distances of Solo-LTR families were also calculated (i.e., group of Solo-LTRs that share more than 90% of total identity) (Figure 3a–c).

Notably, at the population level, comparison of the 5′ and 3′ LTRs shows higher p-distances than the internal regions for the three LTR superfamilies (Figure 3a–c; boxplots a and c versus b). Although these differences are significant only for the elements in the Pao-Bel superfamily, dispersion of the data for LTR regions (a and c) in the Copia and Gypsy superfamilies is also larger than the distances in the internal regions. It has been previously shown that the noncoding LTR-ULR region, despite its regulatory functional importance, is the most rapidly evolving region of LTR retrotransposons [62]. A similar observation indicating different levels of nucleotide substitutions in different genes was reported for the retroviruses HTLV type I and II [63], suggesting a higher heterogeneity of the LTR regions.

Interestingly, comparison of the 5′ and 3′ LTRs within each full-length sequence shows that these regions are almost identical in all the cases, with a slightly higher dispersion for the Copia elements (Figure 3a–c; boxplots d). This is also evident in the deterioration profiles of these sequences shown in Additional file 2: Figure S1, i.e., even if the sequences present heterogeneity in the LTRs at the population level, the deterioration level is quite symmetrical within each individual sequence.

All of the families containing full-length sequences (with the exception of Gypsy3_AG) ( Additional file 2: Figure S1-t) also contain open reading frames. These previous observations together indicate that these families might still be active or have been recently transposed.

On the other hand, as expected, the families of Solo-LTR sequences belonging to the Pao-Bel and Gypsy superfamilies present highly deteriorated sequences, presenting both nucleotide substitutions and indels or segmental deletions along their alignments ( Additional file 2: Figure S1), indicating their neutral way of evolution.

It has been generally assumed that after transposition, the new copies of LTR elements are full length in size and identical in nucleotide composition to the source sequences and that during evolution, it is expected that these sequences will diverge in sequences and structure due to Solo-LTR formation and indel accumulation [52]. In fact, the level of divergence estimated between copies and consensus or canonical sequences has been used to estimate the ages of insertions in several genomes [52][64]. Recently, Arensburger et al. [39] performed a comparative analysis of the TEs in three mosquitoes genomes, *Aedes aegyptiAn. gambiae,* and *Culex quinquefasciatus,* and used the divergences among the members of different families to compare the relative ages of TEs in the three genomes. They found that both LTR and NLTR retroelements dominated the most recent relative age classes, consistent with the presence of recently active retrotransposons and a gradual degradation of the sequences. Our data showed that alignments of Pao-Bel families present greater heterogeneity than the Copia or the Gypsy families. This heterogeneity is mainly due to the presence of nucleotide substitutions along their alignments with most of the point or segmental deletions being present in the flanking regions. The Copia families appeared to be the most conservative sequences, which together with the absence of Solo_LTRs sequences in this superfamily could indicate a more recent introduction of this superfamily in the *Anopheles* genome. Finally, most of the Gypsy families here analyzed present quite homogeneous sequences, with most of the differences between the sequences being due to the presence of nucleotide substitutions. These data are in agreement with a complete analysis of the Ty3-Gypsy [52], which suggested that the main mechanism driving the evolution of Gypsy elements is the formation of Solo LTRs, which in turn must be subjected to lower selective pressure than the full proviral sequences and therefore persisting longer in the genome, allowing for accumulation of mutations and deletions.

The absence of a spectrum of divergence of class I elements and a relatively high homogeneity of LTR sequences found in the genome of *Saccharomyces cerevisiae* has been attributed to a rapid turnover of copies that become inactivated in that genome by LTR-LTR recombination leading to the formation of Solo-LTRs [17]. On the contrary, Solo-LTRs are mostly absent from the genome of *D. melanogaster*[17], so the LTR degradation depends on different mechanisms.

In the *An. gambiae* genome, the LTR elements are represented by members of the three main superfamilies, which present slightly different characteristics. The Copia elements are represented by families of elements with few copies and high homogeneity among their sequences, besides presenting few Solo-LTRs. These features indicated that these elements are young and active in this genome. On the other hand, both the Pao-Bel and Gypsy families here analyzed have quite heterogeneous sequences, even for putative active elements, presenting several nucleotide substitutions and deletions that are far more representative than insertions, as has been previously described for Gypsy elements [52]. Most of the deletions in these sequences are outside the ORFs or inside a few sequences in the alignment, indicating that for the group of sequences analyzed here, the Solo-LTR is a more important source of LTR-family degradation than the deletion of their sequences.

### Class I, NLTR elements

Five different superfamilies of NLTR elements were analyzed (CR1, I, Jockey, Outcast, and RTE). The profile of deterioration of the CR1 and RTE superfamilies presents the previously described 5′ truncated sequences [54,65-67], giving a stair-like pattern to the sequence alignments ( Additional file 3: Figure S2a-q), while Jockey, I, and Outcast are represented by full-length sequences with no significant differences in the frequencies of indels or nucleotide substitutions along the full-length alignments ( Additional file 3: Figure S2r-v). Most of the families analyzed belong to the CR1 superfamily, which is the most abundant superfamily in the mosquito genome.

**Figure 4:**
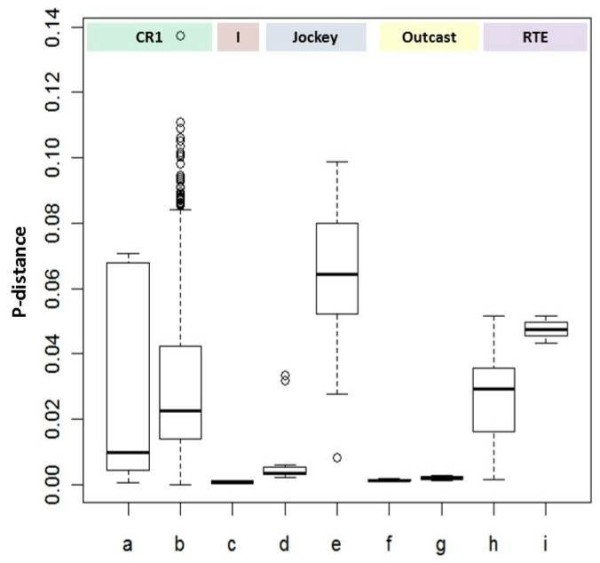
**Number of base substitutions per site (p-distance) from between sequences belonging to five superfamilies of NLTR elements.** See Figure 3 for detailed information. **a**) and **b**) CR1 superfamily: full length (four families) and fragmented sequences (16 families), respectively; **c**) I elements (one full length family); **d**) and **e**) Jockey superfamily, full length (three families) and fragmented sequences (two families), respectively; **f**) and **g**) Outcast superfamily, full length (two families) and fragmented sequences (one family), respectively; **h**) and **i**) RTE superfamily, full length (two families) and fragmented sequences (one family), respectively. We tested the significance of the difference between families containing full-length sequences and those containing only truncated sequences. Kruskal-Wallis test: p-value < 0,001. Ad hoc, Tukey non-parametric test. p-values: a–b = ns; d–e**; h–i*. P-value: ns = not significant; * < 0.05; ** < 0.001.

Analysis of the similarities at the nucleotide level of the sequences in the families of NLTR elements is shown in Figure 4. The p-distances of the families containing full-length sequences corresponding to the Jockey and RTE superfamilies were significantly smaller than their truncated counterparts (Figure 4d,e and h,i), and the Outcast families have very low distances both in their full-length and truncated sequences (Figure 4f, g); however, there were no significant differences between families containing full-length and truncated sequences and those containing only truncated sequences from the CR1 superfamily (Figure 4a, b). CR1 families present a very broad range of p-distances both among full-length and truncated sequences. We have analyzed fifteen CR1 families: five presenting full-length and 5′-truncated sequences ( Additional file 3: Figure S2a–e), eight with only 5′-truncated sequences of different lengths ( Additional file 3: Figure S2f–m), and two composed only of 3′deleted sequences ( Additional file 3: Figure S2n–o). Some of the CR1 families containing both full-length and truncated sequences have large p-distances (more than 6%) (Figure 4a), which might indicate the process of deterioration of the truncated sequences that are also present in the families and included in this analysis. In fact, the deterioration profile of those sequences ( Additional file 3: Figure S2a–e) shows that some of the 5′-truncated sequences from each of the CR1 families analyzed are quite deteriorated, containing nucleotide substitutions, insertions, and deletions. Still, all the full-length sequences within these families contain ORFs and no indels at all; however, closer inspection of the individual sequences in these families showed that several of the 5′ truncated sequences keep ORFs covering the whole ORF2, the RT domain, or even a conserved region in the 3′ of ORF2 with unknown function ( Additional file 3: Figure S2a–e). In some cases, an elevated number of truncated sequences maintain their ORFs (see Additional file 3: Figure S2). On the other hand, some families containing only truncated sequences (Figure 4b) present low p-distances, which might indicate a constraint in the evolution of these truncated sequences. A detailed analysis of truncated sequences in these families also showed that several of the truncated sequences preserved ORFs comprising the regions mentioned before. It is interesting to note that most of the insertions or deletions in the deterioration profiles shown in Additional file 3: Figure S2 are present in the 3′ region after the end of ORF2. This is a very unusual pattern, because most commonly the truncated sequences of CR1 elements lack ORFs at all [68].

**Figure 5:**
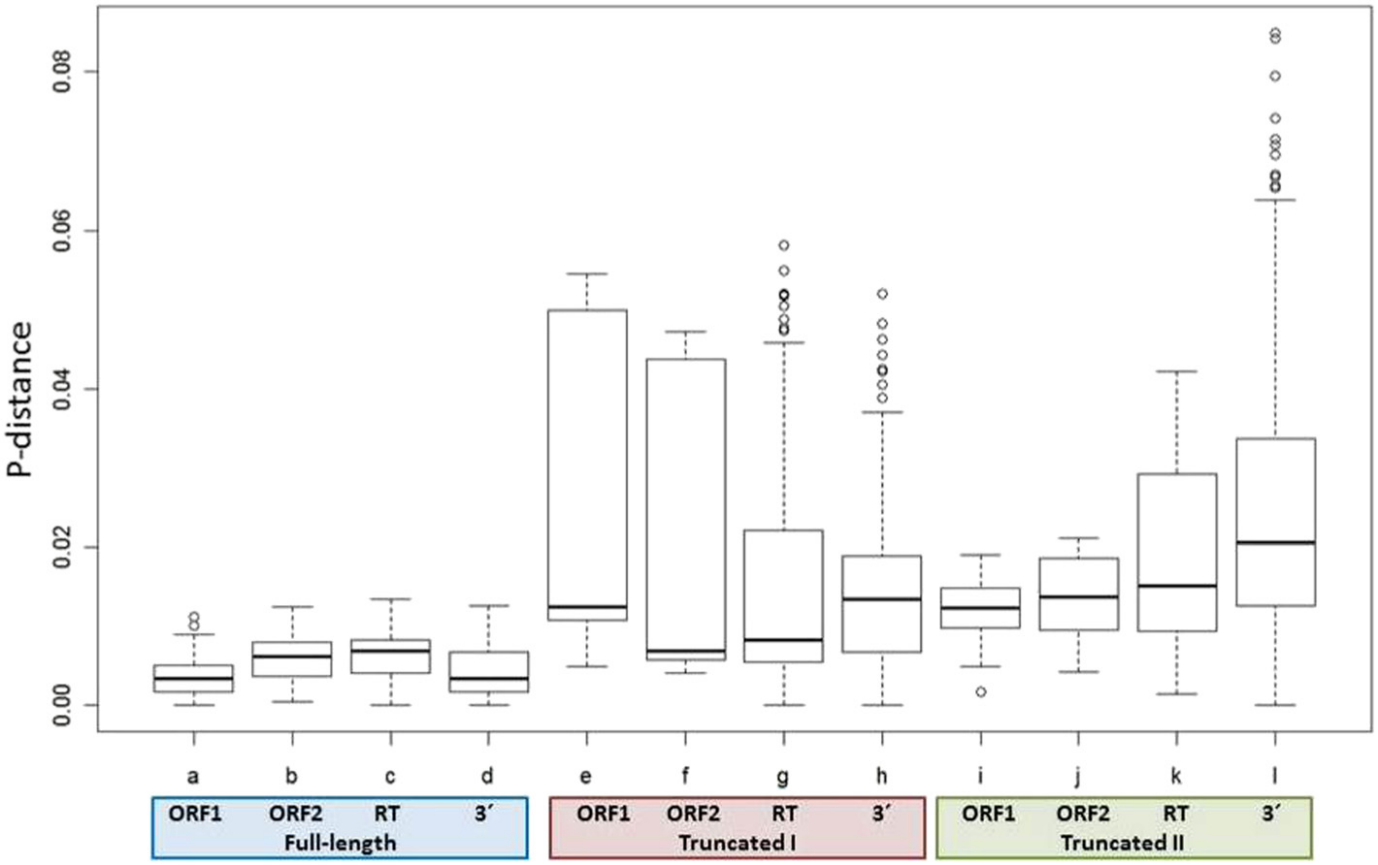
**Number of base substitutions per site (p-distance) from sequences belonging to CR1 elements.** See Figure 3 for detailed information. Lines **a**–**d** represent the p-distances among different regions (ORF1, ORF2, RT, 3′-conserved region) of full-length sequences; lines **e**–**h** represent the p-distances from truncated sequences belonging to the same families analyzed in lines **a**–**d** (truncated I); and lines **i**–**l** represent distances from truncated sequences from families were no full-length sequences were identified (truncated II). The mean p-distances and standard deviation are shown on top of each boxplot. Kruskal-Wallis test: p-value < 0.001. Ad hoc, Tukey non-parametric test: for full length sequences: a–b**, a–c**, d–c*; the rest are not significant. For ORF1 comparisons: a–e***; a–i***; e–I = ns; for ORF2: b–f = *; b–j = *; f–j = ns; for RT: c–g = **; c–k = **; g–k = *; for 3′ conserved region: d–h = ***; d–l = ***; h–l = ***. p-value: ns = not significant; * < 0.05; ** < 0.005, *** < 0.0001.

To better understand the possible significance of the above findings, we further analyzed the CR1 families and classified them into three groups according to the level of sequence deterioration in each family as *i)* only full-length, *ii)* truncated sequences belonging to families where full-length sequences are also present, and *iii)* families composed only of truncated sequences. The multiple sequence alignments analyzed were divided into four regions comprising ORF1, ORF2, the RT domain, and the 3′ conserved region. Analysis of the similarities among the different regions of the full-length and truncated sequences is shown in Figure 5. When considering only the full-length sequences, ORF1 presents significantly less variability than ORF2, including the RT domain, but surprisingly have no significant differences with the 3′ conserved region of ORF2 (Figure 5). This conserved region within ORF2 has no known function, although it appears to be much conserved in NLTRs. There were no significant differences in any of the regions for the two groups of truncated sequences analyzed.

When comparing the different regions for the three groups of sequences, both ORF1 and ORF2 presented significantly lower distances in the full-length than in the truncated sequences from both groups. On the other hand, there were no significant differences in ORFs 1 and 2 for the truncated sequences. For the RT region and the 3′ conserved region, the full-length sequences presented significant lower distances than their truncated counterparts, but in both cases, the truncated sequences belonging to families with full-length sequences presented significantly lower distances among their sequences than the other group of truncated sequences.

**Figure 6:**
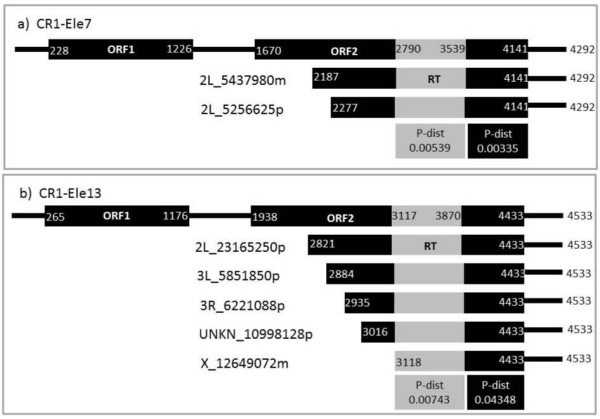
**Schematic representation of truncated sequences spanning the RT domain and 3′ conserved regions for two CR1 families: A)** family CR1-ele7 (cluster 36) and **B)** family CR1-ele13 (cluster 16). Top sequences are canonical sequences for reference. The numbers in white represent the relative positions of each truncated fragment. At the bottom of each panel, the p-distances for each of the regions indicated are shown. The exact chromosomal position of each truncated sequence is shown at the left.

We further analyzed the truncated sequences corresponding to RT and 3′ conserved regions of two families of CR1 elements that contain complete ORFs: CR1-Ele7(clu36) and CR1-Ele13(clu16) contain sequences covering only the complete RT domain and the “3-common” region. Two truncated sequences from family CR1-Ele7, both located in chromosome 2L, are very similar, presenting distances between them both in the RT and in the 3-common region, 0.00539 and 0.00335, respectively (Figure 6a). Five sequences of the CR1-Ele13(clu16) family present a very small distance among them only in the RT region, 0.00743, while the distance of the 3-common region is one order of magnitude higher (Figure 6b). The high similarity among these sequences is surprising, as they are not part of full-length elements. They are all located in different chromosomes. These observations are compatible either with a recent origin of these sequences by an incomplete transcription of full-length elements or with a direct constraint in the evolution of these sequences, such as domestication of the element. We further analyzed expression of these sequences by comparing them by blastn to expression libraries, but they did not show any significant result. It has been previously suggested that genomes might have recurrently recruited TE-derived enzymatic or structural functions for their benefit [69]. In the case of the CR1 families here analyzed, it is surprising that ORF preservation in truncated sequences appears in most of the families. It has been previously noted that DOA elements present low nucleotide polymorphism together with a large number of internal deletions, a fact that has been related to a high rate of DNA loss in *Drosophila*[70]. On the other hand, other NLTR elements previously analyzed as t F, docs, or Jockey elements do not present the same pattern of DNA loss [17].

We also found two families of CR1 elements (CR1-2 (clu46) and CR1-3 (clu17) presenting only 3′ deleted sequences, which is a very unusual pattern for NLTR elements ( Additional file 3: Figure S2n,o). The CR1-2_AG (clu46) family is composed of 20 sequences that were aligned to the respective canonical sequence in Repbase. Twelve of these sequences consist of three blocks of homology with canonical CR1-2_AG covering positions 1–1762, 2150–2600, and 4511–4674; three of the sequences cover the positions 2150–2600 and 4511–4674, and six sequences cover the first 545 nucleotides of the canonical element. The overall similarity of the sequences is high (p-dist = 0.0141, sd = 0.0012) and they present few indels. Eight of these truncated sequences present full-length ORF1 sequences, with p-distances among them of 0.0114 (sd = 0.0015). The 50 sequences belonging to the CR1-3_AG(clu17) family, on the other hand, correspond to the first 820 nucleotides of the canonical sequence, which is 5515 nucleotides long and contain several insertions, deletions, and point substitutions that are evident in the Plotcon analysis ( Additional file 3: Figure S2). A different mechanism than that proposed for the generation of 5′-deleted elements of the NLTR order (DOA)—involving the reverse transcription of these elements—needs to be invoked to explain this finding. Analyses of diverse NLTRs in several species have shown that the 5′ deletion is common among these elements [71-73]. The reverse transcription starts at the 3′ end and is believed to fail to proceed to the 5′ end, generating a large number of copies with varying lengths in the genome [54,66,67]. On the other hand, complete absence of 5′-deleted NLTRs in other species has also been described. In the rotifer *Adineta vaga,* NLTRs have been found to be inactivated by internal deletions, while no 5′ truncated elements were found [74]. A mechanism related to the target site-primed reverse transcription of NLTR elements is normally used to explain the 5′-deleted elements; however, considering the total absence of these types of truncated elements in other NLTR families, together with the fact that reverse transcription is the mechanism used to replicate these families—which in many species are shown to be extremely successful families (e.g., Alu sequences in humans)—indicate the possible presence of a different, yet unknown, mechanism involved in this process.

### Class II

**Figure 7:**
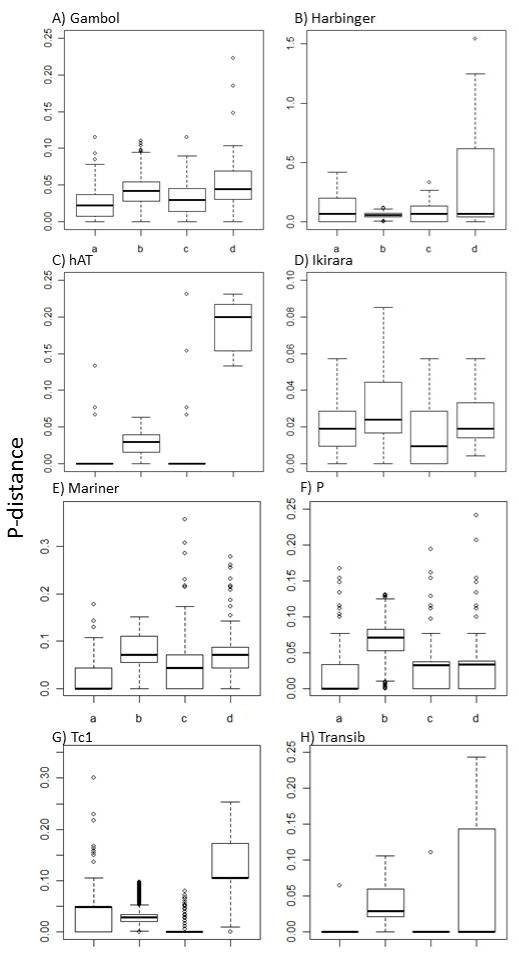
**Number of base substitutions per site (p-distance) from between sequences belonging to eight superfamilies of class II elements.** See Figure 3 for detailed information. **A**) Gambol (seven families). Kruskal-Wallis test: p-value = **. Ad hoc, Tukey non-parametric test: p-value = ** for all comparisons except b–d that was ns. **B**) Harbinger (three families). Kruskal-Wallis test: p-value = **. Ad hoc, Tukey non-parametric test: p-value a–b = **; a–c = **; a–d = ns; b–c = ns; b–d = ns; c–d = **. **C**) hAT (four families). Kruskal-Wallis test: p-value = **. Ad hoc, Tukey non-parametric test: all comparisons were significant at p < 0.001 except a–c = ns. **D**) Ikirara (two families). Kruskal-Wallis test: p-value = **. Ad hoc, Tukey non-parametric test: a–b = **; a–c = ns; a–d = ns; b–c = **; b–d = ns, c–d = ns. **E**) Mariner (seven families). Kruskal-Wallis test: p-value = **, Ad hoc, Tukey non-parametric test: integer overflow. **F**) P (five families). Kruskal-Wallis test: p-value = **. Ad hoc, Tukey non-parametric test: integer overflow. **G**) Tc1 (six families). Kruskal-Wallis test: p-value = ** Ad hoc, Tukey non-parametric test: integer overflow. **H**) Transib (Two families). Kruskal-Wallis test: p-value = **. Ad hoc, Tukey non-parametric test: all comparisons were significant at p < 0.001 except b–d = ns. P-value: ns = not significant; * < 0.05; ** < 0.001. **a**) 5′-TIR (population comparison), **b**) internal region (population comparison), **c**) 3′-TIR (population comparison), **d**) 5′-TIR to 3′-TIR within element (performed for each element within each of the populations analyzed). On top of each boxplot, the mean and standard deviation for the p-distances in each group of sequences are shown.

Class II elements clearly represent the most heterogeneous set of sequences of all the TE families in *An. gambiae*. For all the families analyzed, the profiles of deterioration show heavily deleted sequences ( Additional file 4: Figure S3), and the distances among their sequences present broad differences (Figure 7a–h). Most of the families are represented by putative MITE elements, i.e., groups of sequences sharing TIRs with known or even unknown class II elements that are amplified by the transposases of active elements in the genome.

Some of the MITE-like sequences analyzed share TIRs with recognizable elements present in the mosquito genome, as is the case of the Gambol elements, hAT and Harbinger; however, for some families—although they have a MITE-like structure due to their small size, absence of coding capacity, and presence of TIRs—there is no obvious full-length counterpart described in the *An. gambiae* genome so far. This is the case for all the marinerN elements harbingerN1 and N2, hatN1, as well as of a series of MITE-like described in the genome that do not show any similarity with known class II elements in their TIRs or their internal regions [27].

All the Gambol elements analyzed correspond to MITE-like sequences, which have been previously described [27]. Interestingly, they present shorter TIRs than the canonical elements, which in one case have different sizes (Gambol_ele3). The TIRs of the canonical Gambol families are larger than typical TIRs of class II elements, while the TIRs of the MITE like sequences are shorter. This might indicate that a smaller portion of the TIR is necessary for proper recognition by the active transposase. On the other hand, TIRs are very similar at the population level with slightly higher distances for the internal regions (Figure 7A:a–c). The 5′ to 3′ TIR comparison reveals differences at the nucleotide composition of the flanking TIRs in certain sequences (Figure 7A:d).

The Harbinger and hAT elements also present MITE-like structures with families presenting imperfect TIRs, which is revealed by the larger p-distances in the 3-5′ nucleotide similarities comparison (Figure 7B:d and C:d). The 5′and 3′ TIRs for the hAT families are identical at the population level, favoring the idea that they might have transposed recently.

The Ikirara, Mariner, and P elements (Figure 7D–F) show larger similarity distances in their 5′ and 3′ TIRs. The Mariner sequences seem to be composed of MITE-like families and have been previously characterized as MarinerN families (Repbase). There are 21 sequences of this type of element described in *An. gambiae* (Repbase reports). Most of them contain imperfect TIRs; two of the families present sequences lacking either the 3′ or the 5′ region and therefore lack one TIR, which probably makes them dead elements. They have been compared to the MarinerN elements to which they belong ( Additional file 4: Figure S3), as they do not share identical TIRs with any full-length Mariner. The P MITE elements [35] have also been compared to the MITE sequences already described as such ( Additional file 4: Figure S3). The Tc1 families present heterogeneous 5′ TIRs (Figure 7G:a) but conserved 3′ TIRs (Figure 7G:c) at the population level, and both TIRs are quite different within the same sequences (Figure 7G:d). Finally, the Transib elements (Figure 7H) have almost identical 5′and 3′ TIRs at the population level, although they have imperfect TIRs ( Additional file 4: Figure S3).

Some of the families analyzed are quite heterogeneous in relation to the canonical sequences of the families to which they belong, giving very irregular profiles on the Plotcon analysis ( Additional file 4: Figure S3), but they are quite homogeneous within their own families. This probably means that they constitute subfamilies of MITEs that are different from the sequences used to create the consensus sequences deposited in Repbase.

Finally, we have based our analysis on the families of deteriorated elements that we found in our previous study and that are present in AnoTExcel [27]. We did not find any member of the Herves family and therefore did not include it in the present analysis.

## Conclusions

We described an *in silico* analysis of elements representing the main superfamilies of TEs identified in the genome of *An. gambiae*. Our study concentrates on the deterioration pattern of the TEs in this genome and is based on families presenting both full-length and truncated sequences.

It is not an easy task to represent the deterioration profile of sequences that are repetitive and that have undergone nucleotide substitutions, insertions, deletions, and recombinations during their genomic evolution. Although there have been previous works on the well characterized and model organism, *D. melanogaster*[17,64], there are to our knowledge few reports that have presented a description of the different structural variations and nucleotide polymorphisms of the various TEs in other genomes. This information is of great importance when trying to understand how these elements replicate, amplify, degrade, and are eventually eliminated from a genome.

One such study is that of Lerat et al. [17] that presented an analysis of sequence divergence of 23 transposable element families in the *D. melanogaster* genome. These authors found a high degree of homogeneity and a lack of divergent elements between sequences of TEs within a given family. On the other hand, they found divergent elements displaying very low percentage of similarities to the full-length sequences. These findings suggested that TEs are highly active within the genome and that the highly divergent copies reflect relics that have been degenerated and rearranged. In the *An. gambiae* genome, several reports have described the evolution and dynamics of different superfamilies of TEs (e.g., [27,42,43,52]); however, as far as we are concerned, there is no previous report dealing with TEs belonging to all the main superfamilies in this genome.

Here we present the deterioration landscape of most of the TE superfamilies described in *An. gambiae*. We chose to show the patterns of deterioration as a graphic representation of the different elements present in this particular genome. This approach has not been used before, and we believe that it helps to reveal the overall differences identified in the TE families of the *An. gambiae* genome.

In any given genome, TEs might present significant diversity regarding the level of sequence deterioration of a given family. The relationship between TEs and the genome where they reside produces particular conditions for evolution of different TE families, and the success or failure in settling and amplifying within a genome would be the result of interactions and evolutionary dynamics between the element and the specific genome. What different strategies can be adopted by different classes of TEs—and what evolutionary forces are involved in this process— are still unanswered questions.

We have not found truncated LTR elements other than the Solo-LTRs; on the other hand, a great part of the NLTRs appears as truncated sequences. In addition, most of the class II are MITE-like elements with highly degraded copies. Overall, we found less divergence for class I (both LTR and NLTR families) than for class II families. A similar finding was described in the *Drosophila* genome [17]. This result is somewhat surprising, as it is well known that replication based on reverse transcriptases (as is the case of retroelements and retroviruses as well) are quite error prone mechanisms [75,76]. Indels are present in several LTR families and are evident in the deterioration profiles in Additional file 2: Figure S1, tending to be more frequent at the 5′ and 3′ ends of the elements. The absence of divergence of the LTR copies could be interpreted as a rapid turnover of the elements once they have been inactivated.

For NLTRs, besides the 5′ truncated sequences previously described, the elements tend to be quite similar within families. They contain indels and point mutations throughout their lengths, but the internal deletions do not seem to be a driving force in their deterioration process as has been previously observed in *Drosophila*[77-79]. It is also interesting to note the high frequency of truncated sequences preserving ORFs. The presence of truncated sequences belonging to biologically important domains—such as the reverse transcriptase in the NLTR elements with very small nucleotide distances among some of the sequences—is intriguing. One would expect that the truncated sequences would have the same time of evolution than other truncated sequences of the same family, and therefore, according to a molecular clock, these neutral sequences might have accumulated mutations at more or less the same rate. This is not the case for families CR1-ele7(clu36) and ele13(clu16). It might well be that they have been domesticated by the host genome in a certain manner.

On the other hand, the distances among the sequences of the same families for the class II elements tend to be larger than the NLTRs and LTRs. The high sequence variation and the indels between different copies of the same family, mainly for the MITE-like elements, indicate they are ancient sequences in the *An. gambiae* genome.

Although it is possible to compare the relative ages of TEs belonging to the same family found in different genomes based on their level of nucleotide divergence [17,39,52], comparison of ages of elements belonging to different orders/classes or even families is more complicated, because the mechanisms responsible for their replication involves quite different processes. For instance, it is well known that the replication of retroviruses is a very error-prone process due in particular to the lack of proof reading repair activity of the RNA polymerase and reverse transcriptase enzymes (review in [80]). As retrotransposition of class I elements resembles retroviral replication, it is reasonable to think that this process might also be error prone. In fact, the mutation rate during a single transposition cycle of the yeast Ty1 element has been estimated to be 2.5 × 10^–5^ substitution/nucleotide [81]—as high as that for retroviruses. We do not have data regarding mutation rates associated with different retrotransposons in *An gambiae*. Given the lack of information regarding the dynamics and rates of evolution of these elements, we consider it safer not to make comparisons on the relative ages of the different classes of elements in this genome.

In summary, we show here that the transposable elements in the *An. gambiae* genome deteriorate in different ways according to the class to which they belong. This diversity certainly has implications not only at the host genomic level, but also at the amplification dynamic and evolution of the TE families themselves.

## Methods

### Pipeline description

The TE families analyzed in this work were extracted from AnoTExcel, a TE-specific database from the *An. gambiae* genome [27]. Sequence alignments belonging to the main classes of TEs in *An. gambiae* were further analyzed. We used families containing elements at different deterioration levels. We analyzed 33 families of LTR elements (12 Pao-Bel with full length sequences, and 3 with Solo-LTRs; 4 Copia with full-length sequences; and 14 Gypsy families—6 with full-length elements and 8 with Solo-LTRs), 24 families of NLTRs (16 CR1; 1 I; 3 Jockey; 2 Outcast; and 2 RTE), and 21 class II (4 Gambol; 3 Harbinger; 2 hAT; 1 Tsessebeii; 5 Mariners; 2 P; 1 Tc1; and 2 Transib).

### Sequence similarity (Plotcon)

Similarity along the sequences was calculated by moving a window of different lengths along the aligned sequences ([82]). Within the window, the similarity of each position is taken to be the average of all the possible pairwise scores of the bases or residues at that position. The pairwise scores are taken from the specified similarity matrix. The average of the position similarities within the window is plotted against the positions in the alignment. The average similarity is calculated as:

(1) $$\text{Av}.\text{Sim}.=\frac{\text{sum}\left({\text{M}}_{\text{ij}}{*}_{\text{wi}}+{\text{M}}_{\text{ji}}{*}_{\text{wj}}\right)}{\left({\text{N}}_{\text{seq}}*{\text{W}}_{\text{size}}\right)*\left(\left({\text{N}}_{\text{seq}}-1\right)*{\text{W}}_{\text{size}}\right)}$$

sum, over column*window size. w, sequence weighting; M, matrix comparison table; i,j, with respect to residue i or j; N_seq,_ number of sequences in the alignment; W_size_, window size.

### p-Distances

The number of base substitutions per site between sequences were calculated using MEGA 5.0 [83]. Analyses were conducted using the Kimura 2-parameter model. Rate variation among sites was modeled with a gamma distribution (shape parameter = 1). All ambiguous positions were removed for each sequence pair.

### Statistics

Data analysis was carried out with the R System (R version 2.14) [84]. We used non-parametric tests Kruskal-Wallis [85] and non-parametric ad-hoc Tukey test [86]. Differences were considered significant at p values < 0.05.

## Abbreviations

Clu: Cluster; DOA: Dead-on-arrival; EST: Expressed sequence tag; LTR: Long terminal repeat; Mb: Megabase; MITE: Miniature inverted TE; NLTR: Non-LTR element; ORF: Open reading frame; RB: Repbase; RT: Reverse transcriptase; TE: Transposable element; TF: TEfam; TIR: Terminal inverted repeat; TRIM: Terminal-repeat retrotransposons in miniature.

## Competing interests

The authors declare that they have no competing interests.

## Authors’ contributions

RDFM conceived the report, participated in its design, performed data analysis, interpreted results, and drafted the manuscript. CMAC, JMR, and CJS participated in intellectual discussion and manuscript drafting. JMR: helped with bioinformatics. LV helped with statistics. All authors read and approved the final manuscript.

## Supplementary Material

Additional file 1 ** Tabl S1. Catalog of transposable elements described in *Anopheles gambiae* to date.** There are three worksheets in the excel file named “LTRs”, “NLTRs,” and “CLASS II”. Each contains information regarding the main TE classes and orders of the respective elements. The source where the specific family was described for the first time is presented. Each worksheet contains a table describing the number of full-length, truncated sequences, Solo-LTRs, and MITEs (for the LTR and class II, respectively) and the total number of families for each superfamily. Click here for file

Additional file 2 ** Figure S1. Graphic representation of the deterioration profiles for the LTR superfamilies and families analyzed in this study.** The graphics performed with Plotcon (http://emboss.bioinformatics.nl/cgi-bin/emboss/plotcon) represent the similarity of the sequences along multiple alignments performed for several families belonging to the LTR superfamilies: Pao-Bel: (**a**–**l**) correspond to families of full-length sequences, (**m**–**o**) correspond to families of Solo-LTR sequences; Copia superfamily (**p**–**s**) correspond to families of full-length sequences; and the Gypsy superfamily: (**t**–**y**) correspond to families of full-length sequences, (**z**–**ac**) correspond to families of Solo-LTRs. Each family was aligned to the respective reference sequence as described in Repbase. The family name and the cluster number according to the AnoTExcel numbering (in parenthesis), are indicated in each graph as. Red circles are included to highlight regions where the differences between sequences are mainly due to indels; blue circles indicate regions where the differences are due to nucleotide substitutions; and green circles indicate regions where segmental deletions are present. Red asterisks are included to indicate single deletions and blue asterisks single insertions. The horizontal green bars represent the ORFs in the alignments, with numbers indicating their relative position to the first nucleotide of the alignment. Green arrows at the top of each graph indicated the relative position of the LTRs; the numbers within the arrows indicate the length of the LTRs. The X axis for all plots refers to the relative residue position in each alignment and the Y axis to their similarity indicated as the pairwise scores that are taken from the specified similarity matrix (see Methods section for detailed information). (PDF 331 kb) Click here for file

Additional file 3 ** Figure S2. Graphic representation of the deterioration profiles for the non-LTR families analyzed in this study.** See legend for Additional file 1: Figure S1 for detailed information. (**a**-**e**) CR1 families composed of full-length and 5′ truncated sequenced; (**f**–**m**) CR1 families composed of 5′ truncated sequences; (**n**,**o**) CR1 families containing 3′ truncated sequences; (**p**,**q**) families of RTE elements composed of full-length and 5′ truncated sequences, (**r**,**s**) Jockey families composed of full-length sequences, (**t**) family of I full-length sequences, (**u**,**v**) families of Outcast elements composed of full-length sequences. Colored arrows indicate the relative positions of ORF1 (blue), ORF2 (red), RT domain (yellow), and 3′ conserved region (green). The relative positions according to multiple alignments of the sequences to canonical element are indicated in each region. The number of sequences represented in each region is indicated above the arrows. (PDF 350 kb) Click here for file

Additional file 4 ** Figure S3. Graphic representation of the deterioration profiles for the class II families analyzed in this study.** See legend for Additional file 1: Figure S1 for detailed information. The blue arrows at the top of each graph represent the position and length of the TIRs in the canonical full-length element. The red arrows indicate the actual TIRs in the MITE-like elements. An “I” after the number indicates incomplete TIRs. Note the different scales on the y-axis. (PDF 201 kb) Click here for file
