# Supplementary figures and images for: Losing identity: structural diversity of transposable elements belonging to different classes in the genome of Anopheles gambiae
Source: BMC Genomics. 2012 Jun 22;13:272. doi: 10.1186/1471-2164-13-272 (PMC3442997; doi:10.1186/1471-2164-13-272)

a)

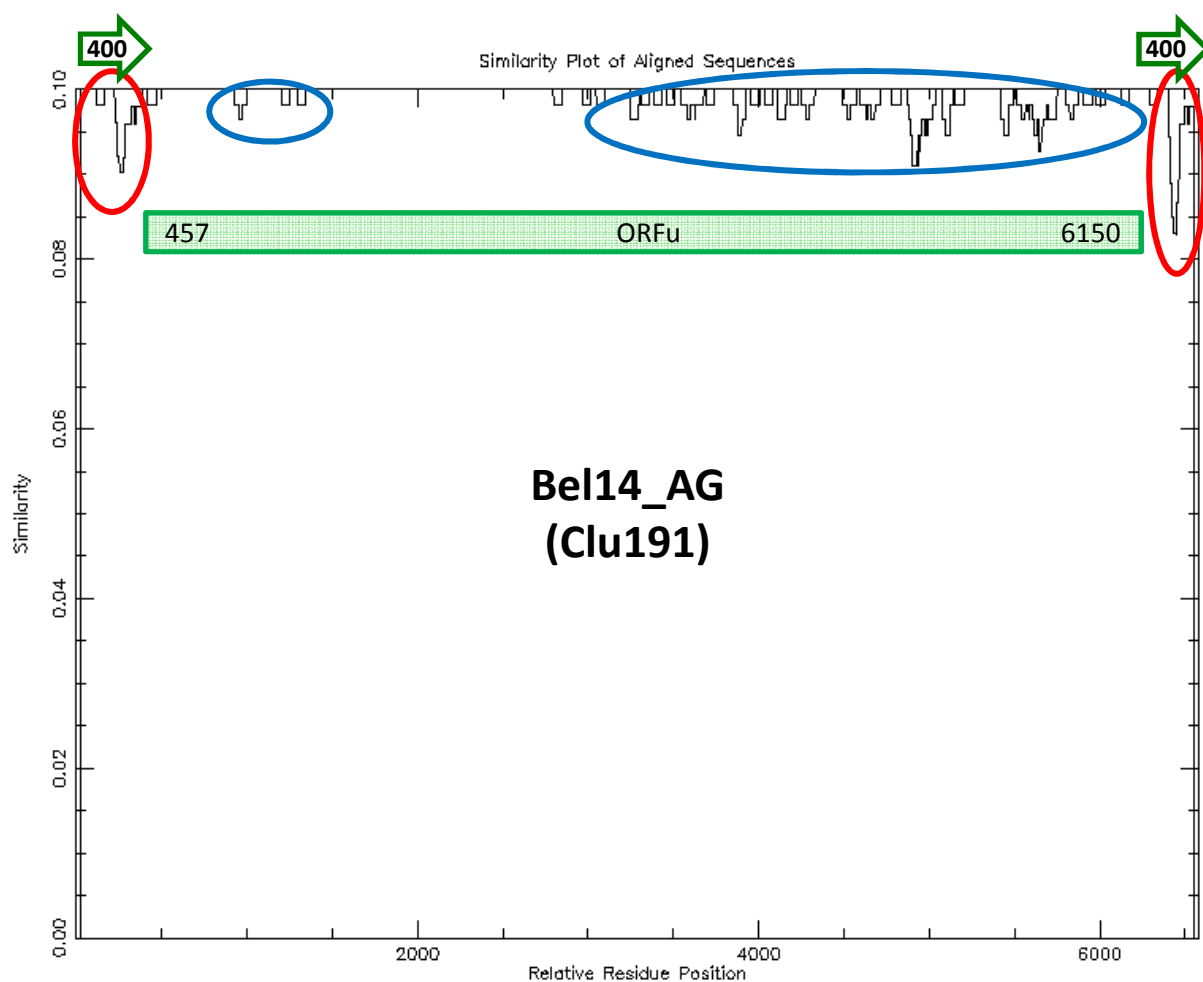

b)

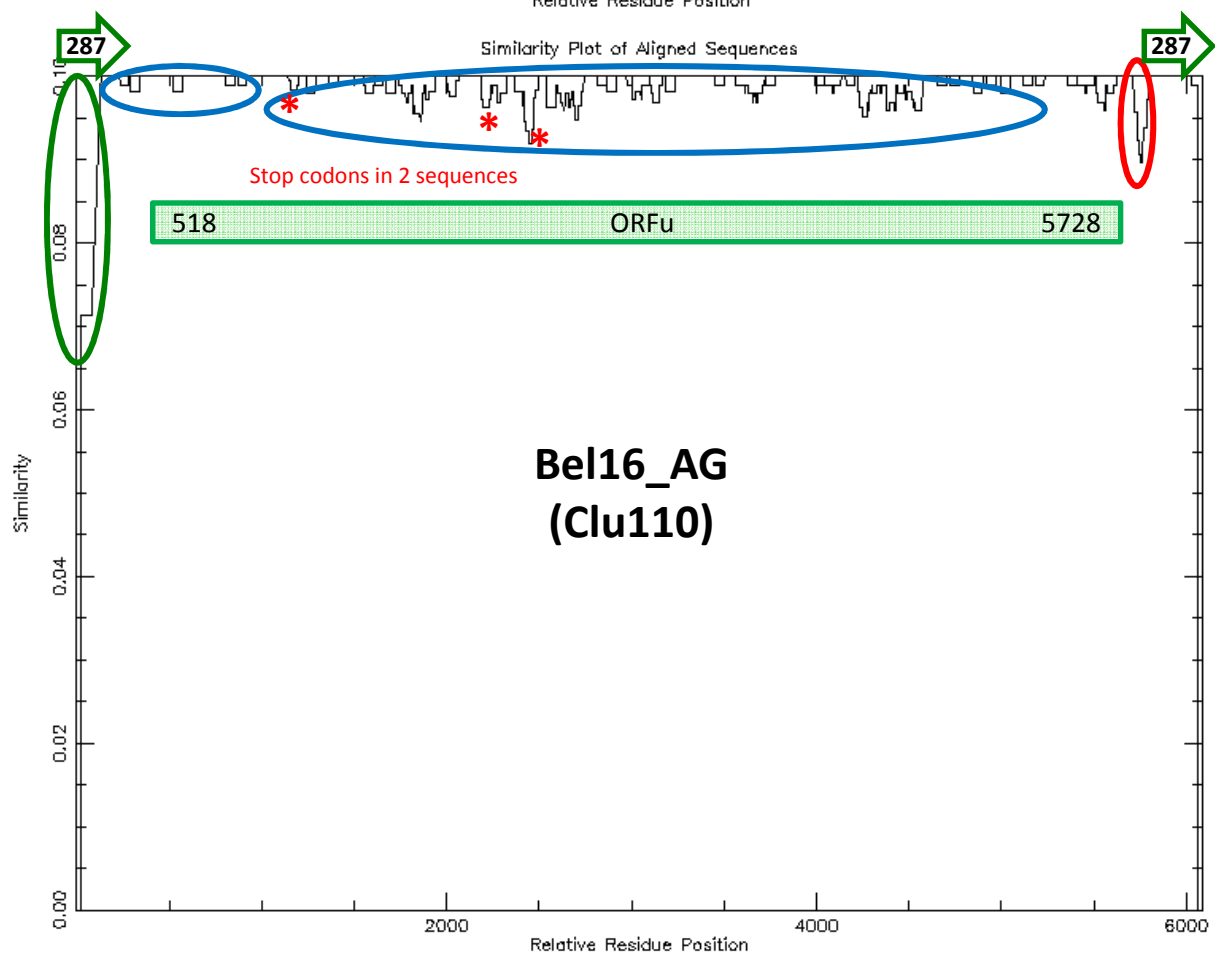

c)

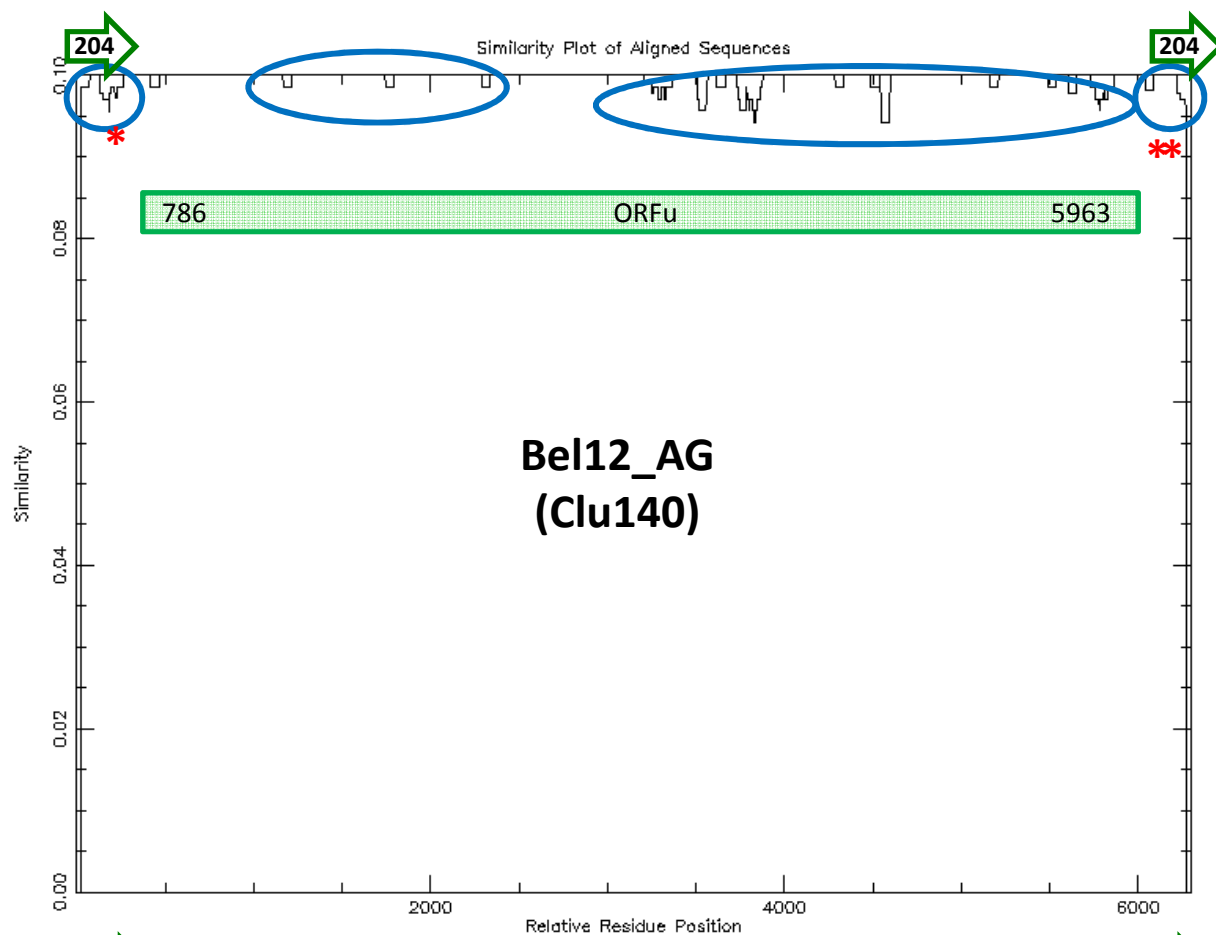

d)

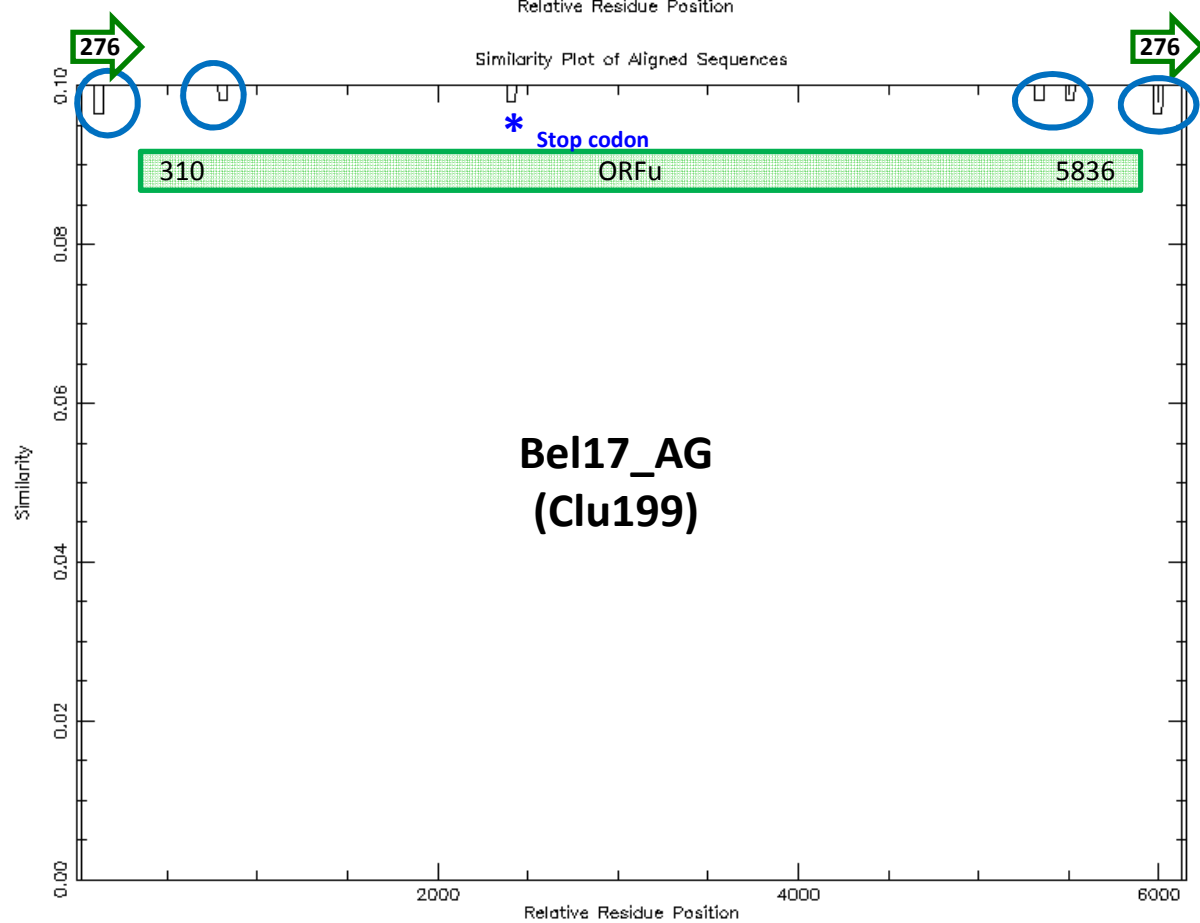

e)

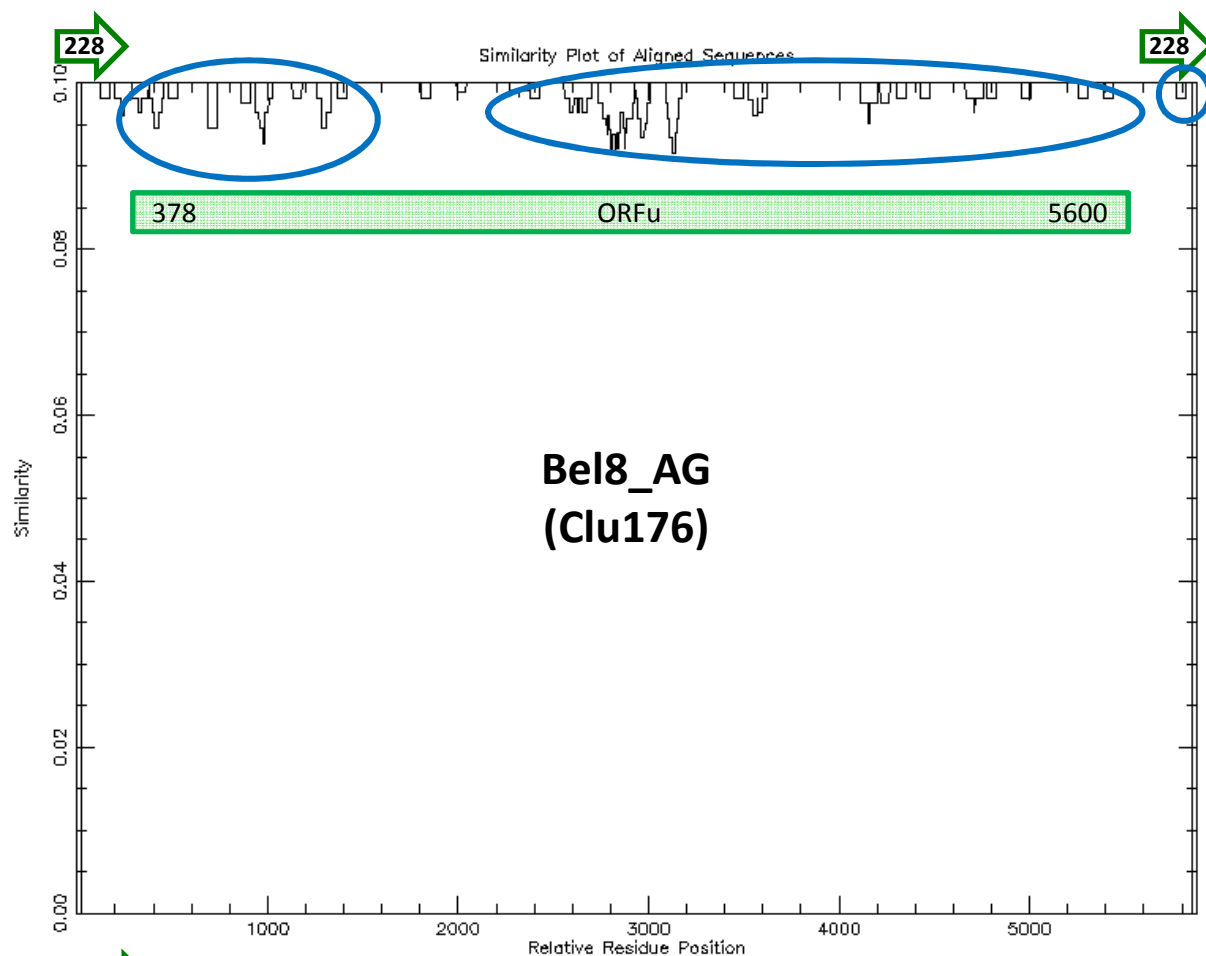

f)

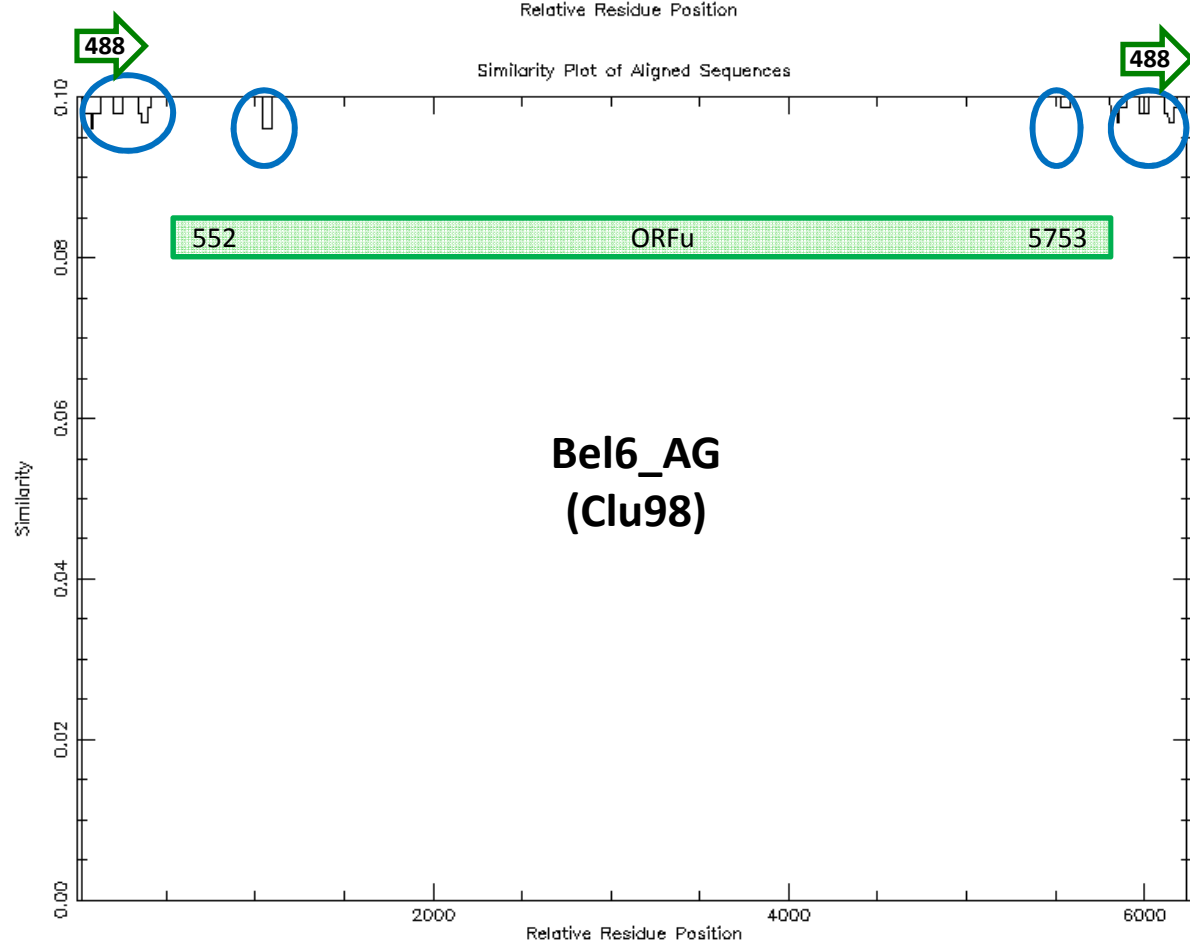

g)

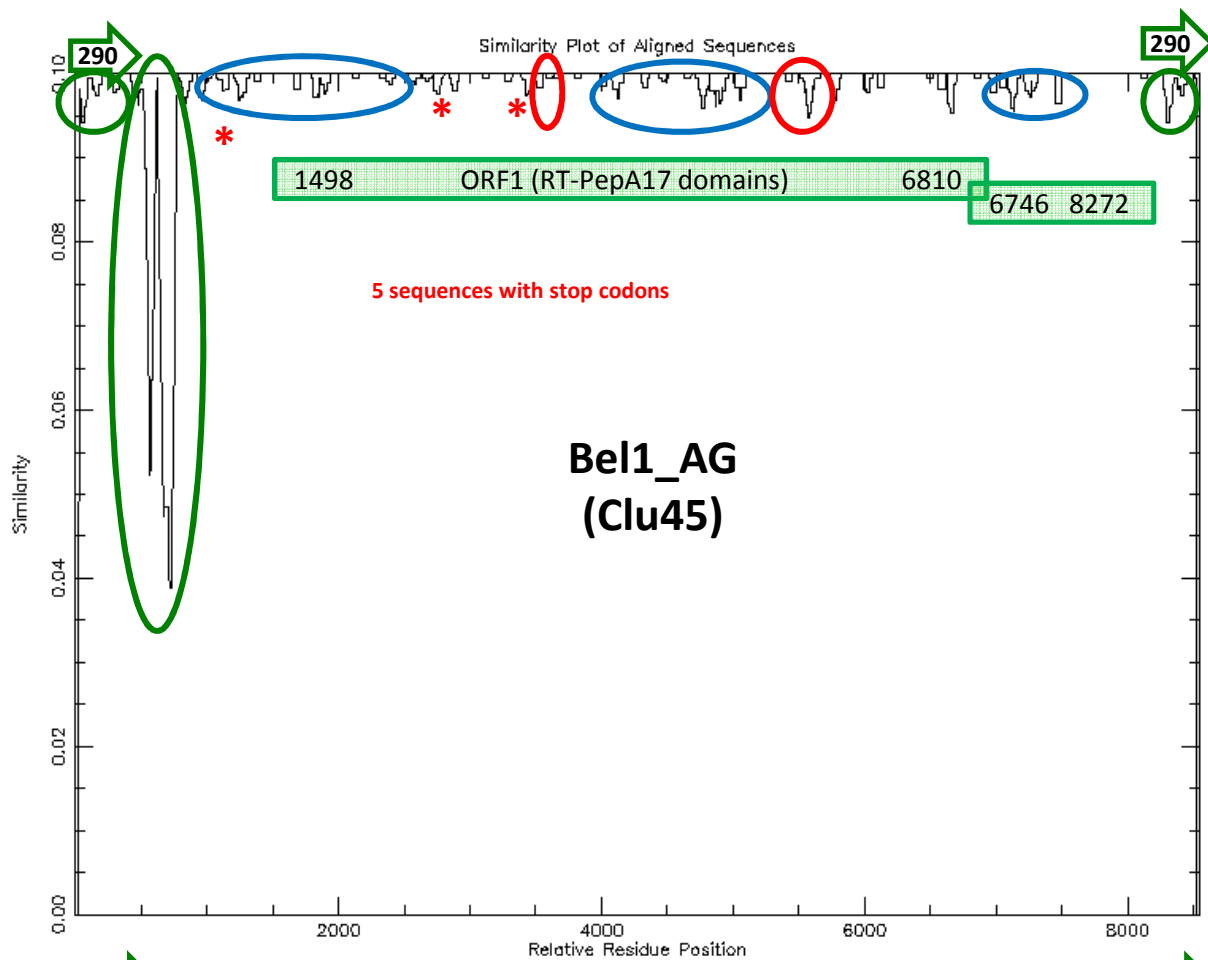

h)

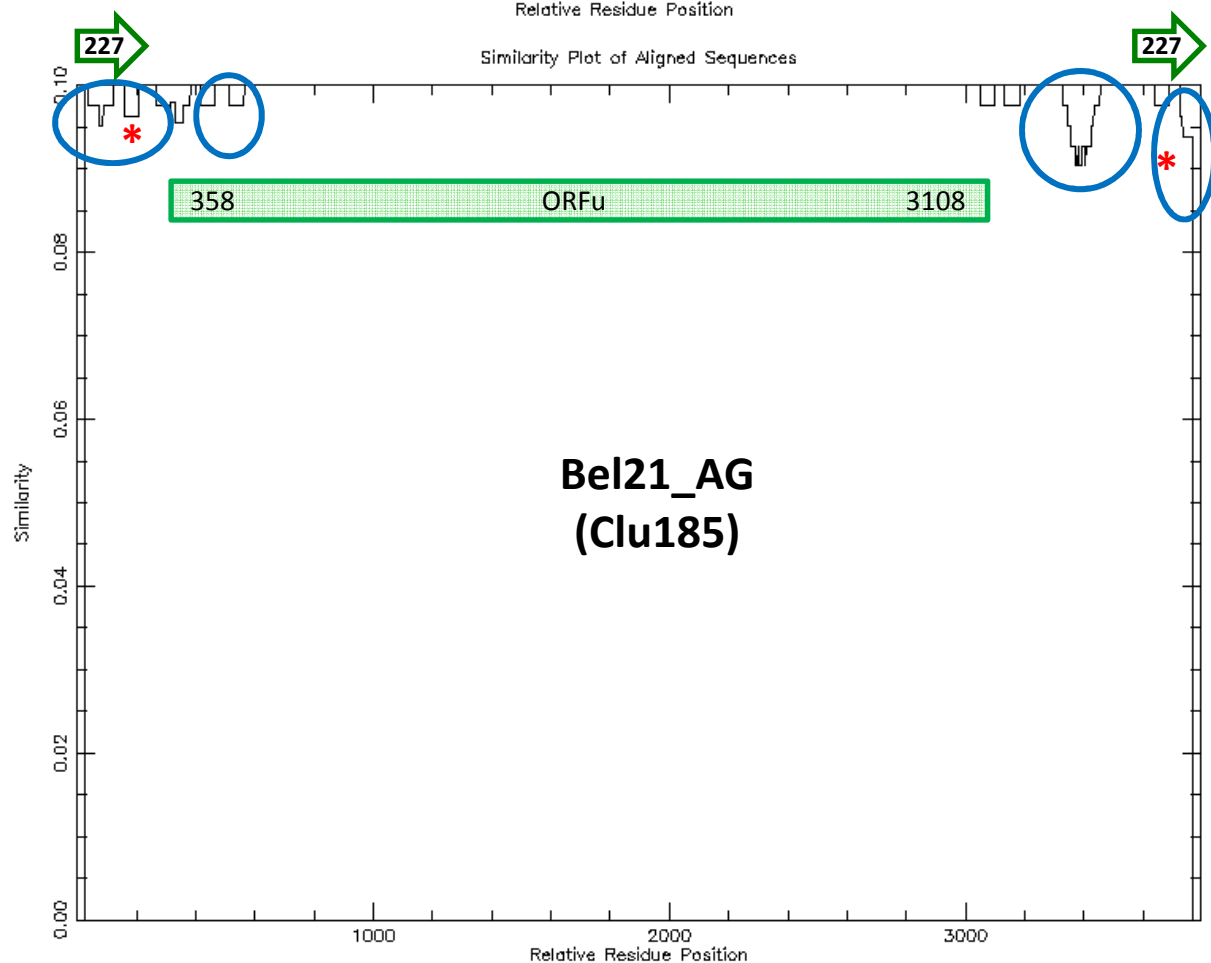

i)

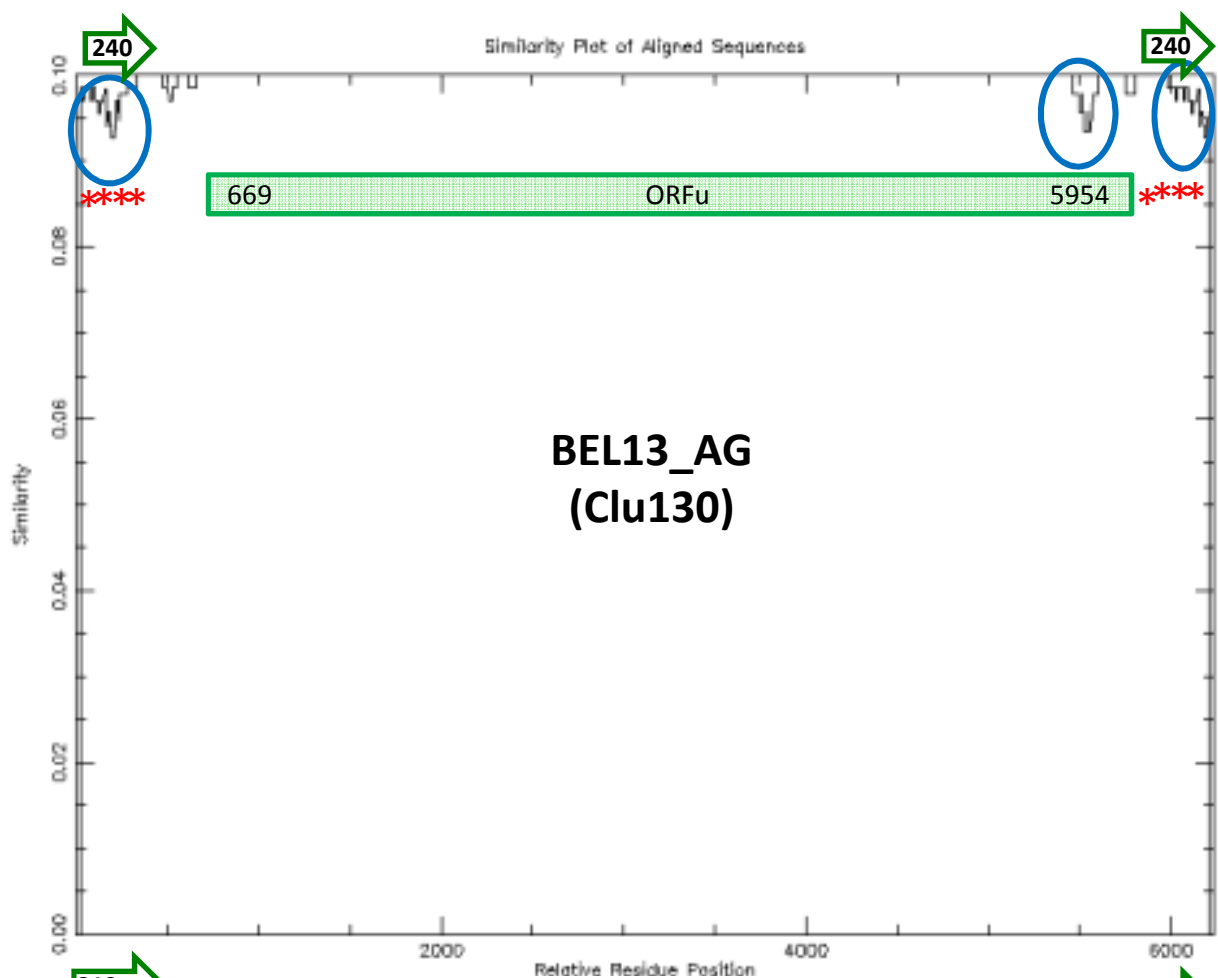

j)

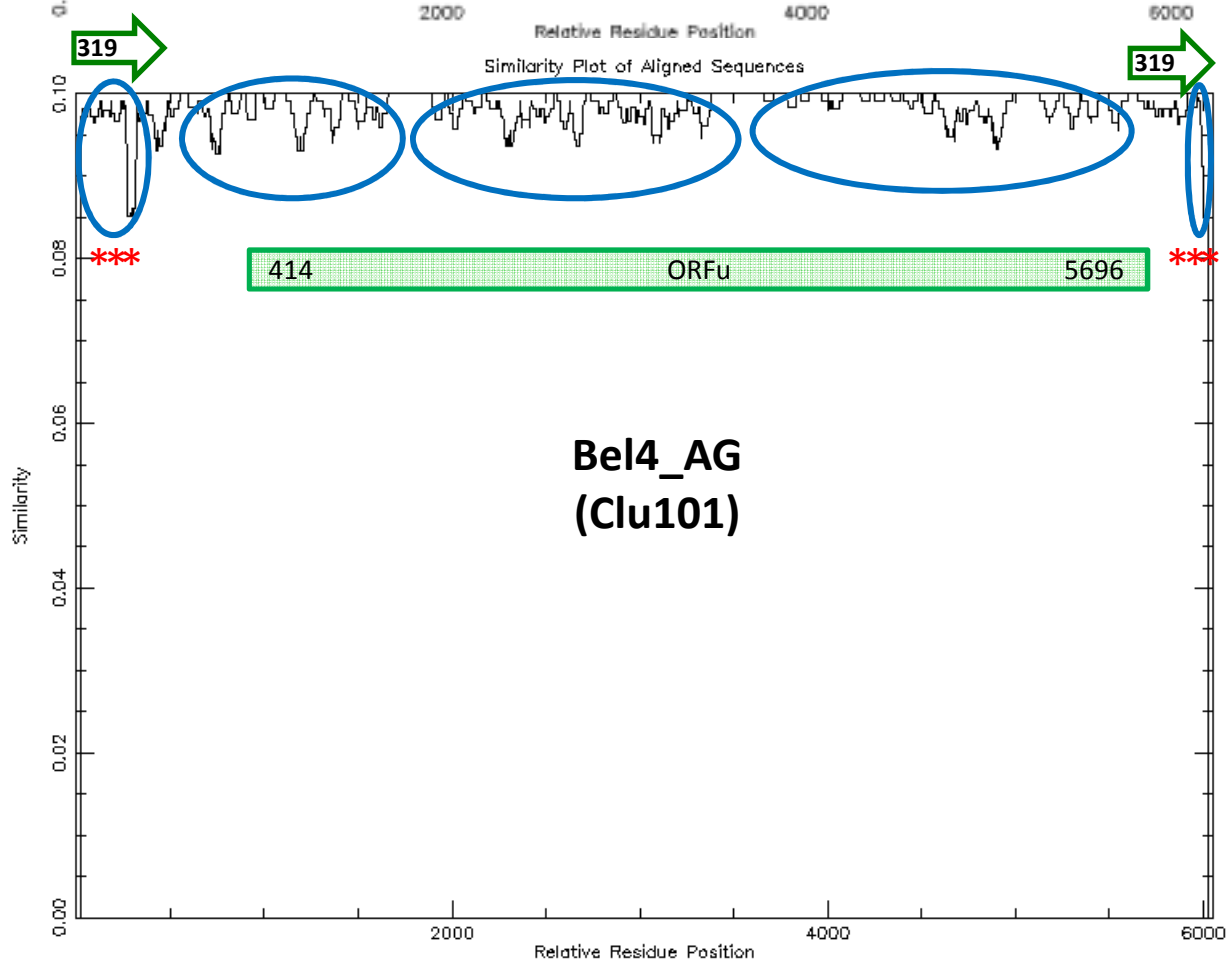

k)

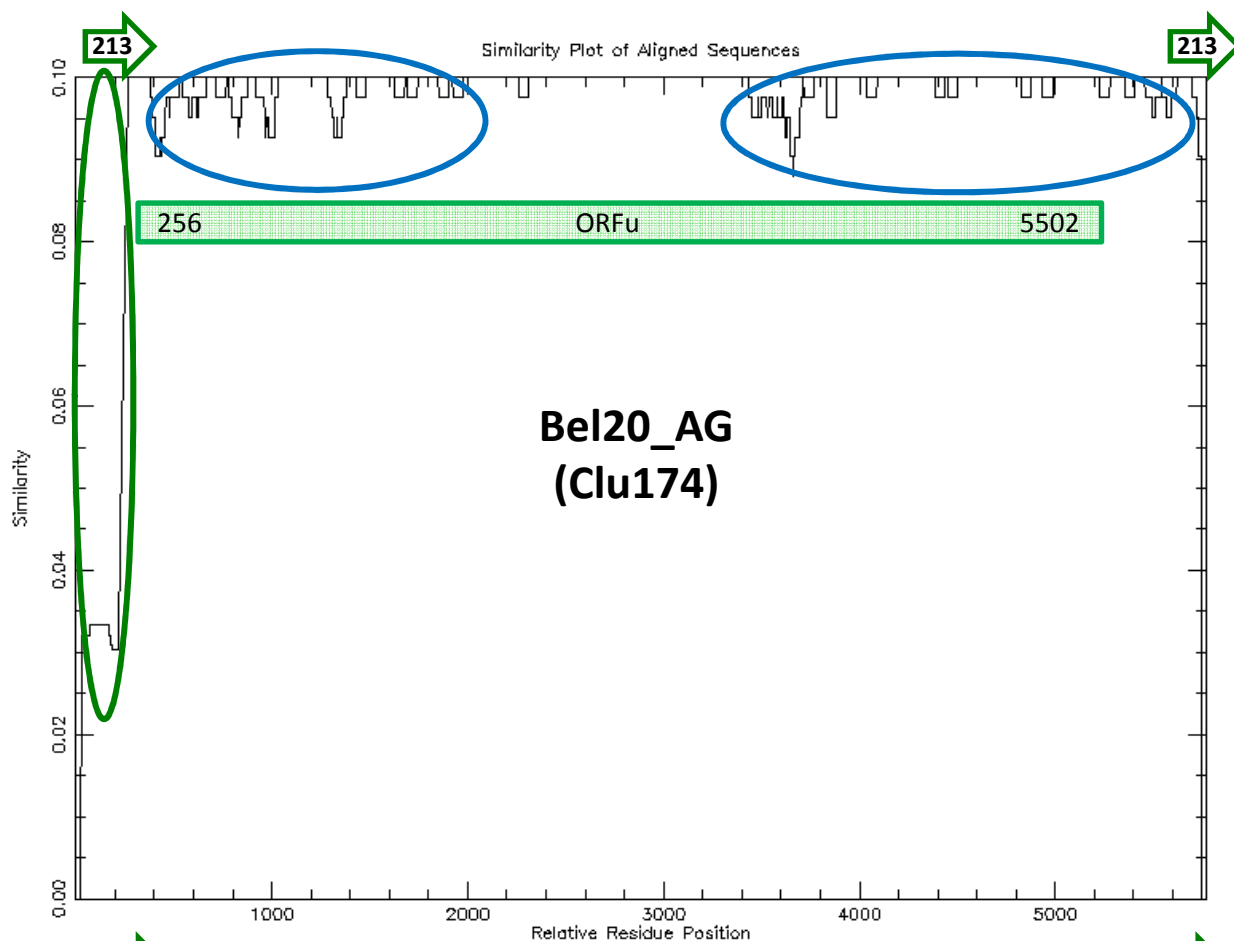

l)

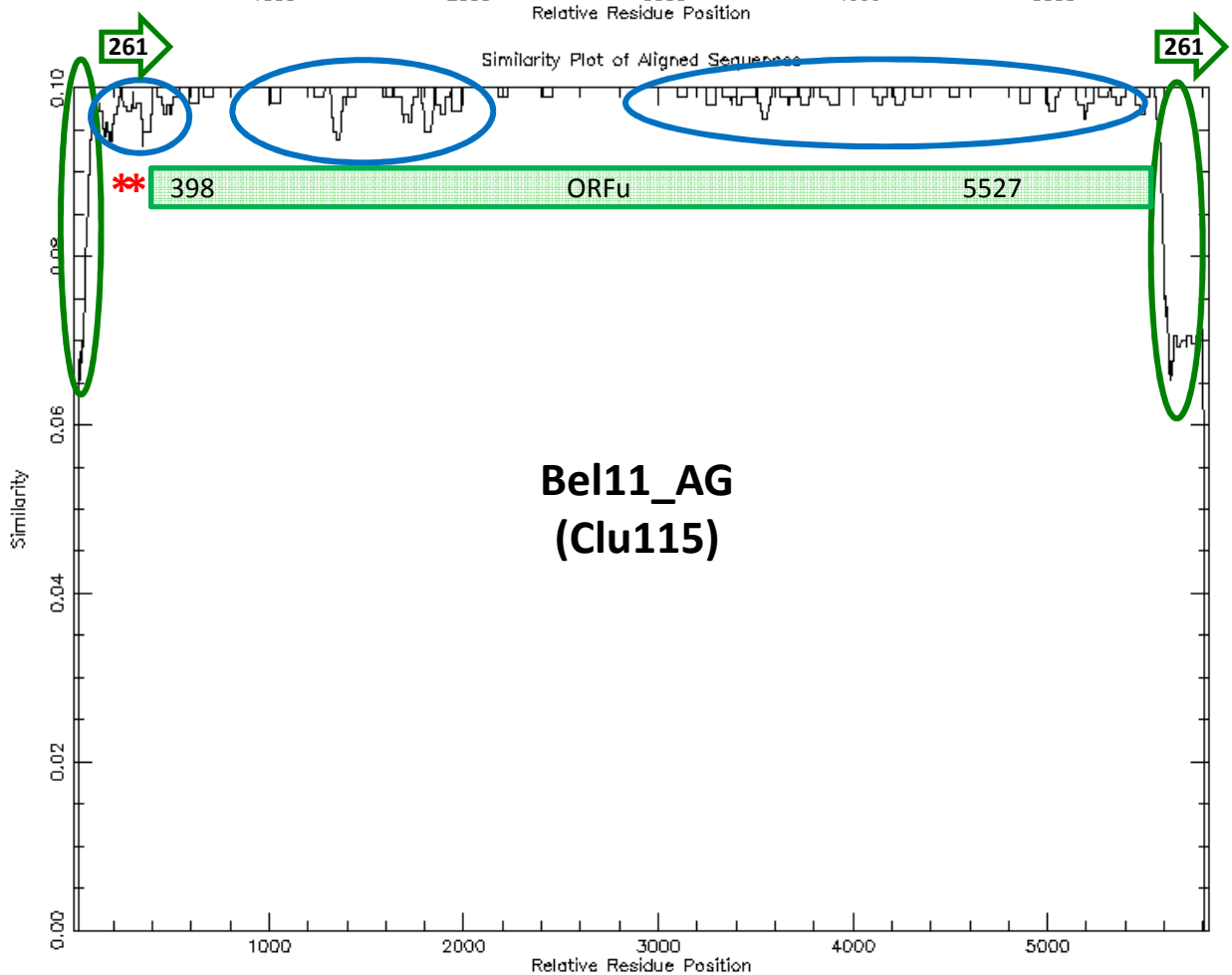

m)

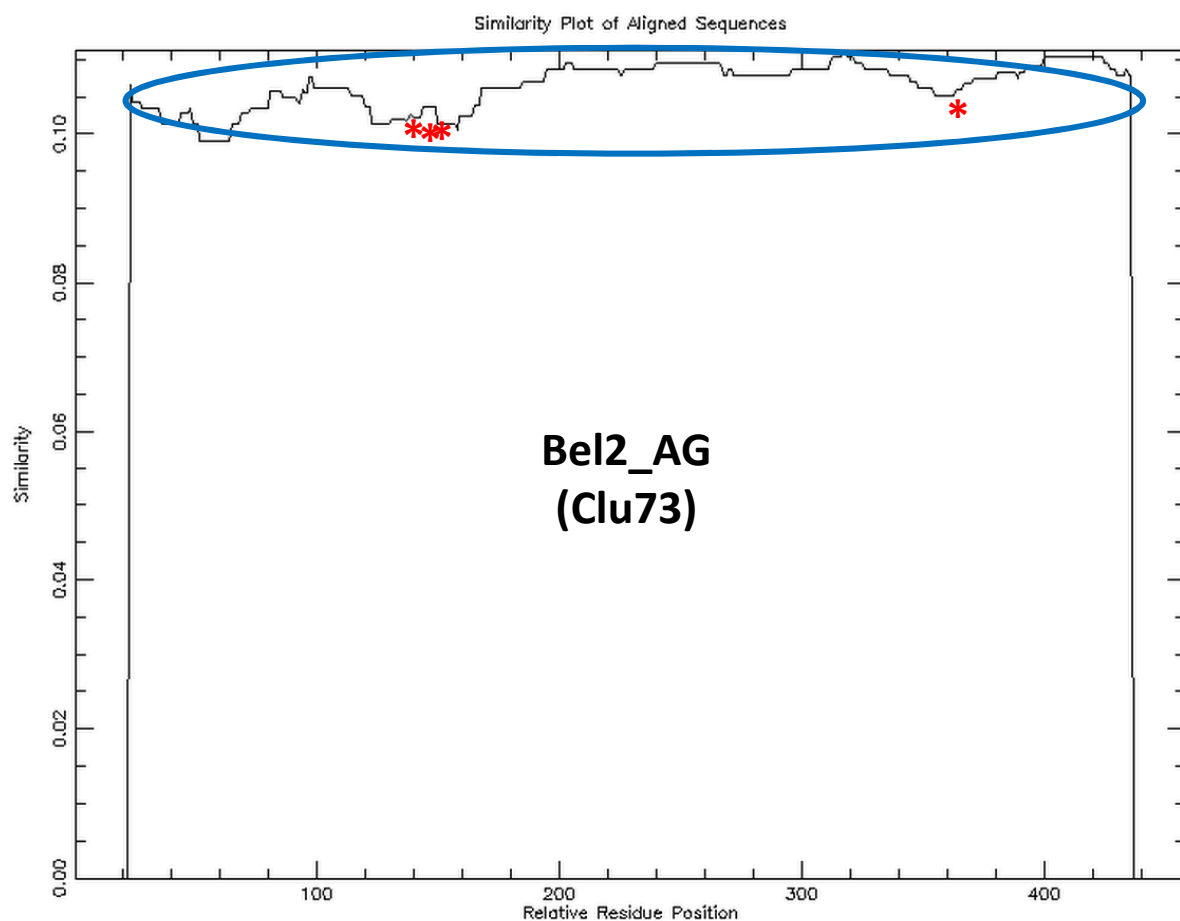

n)

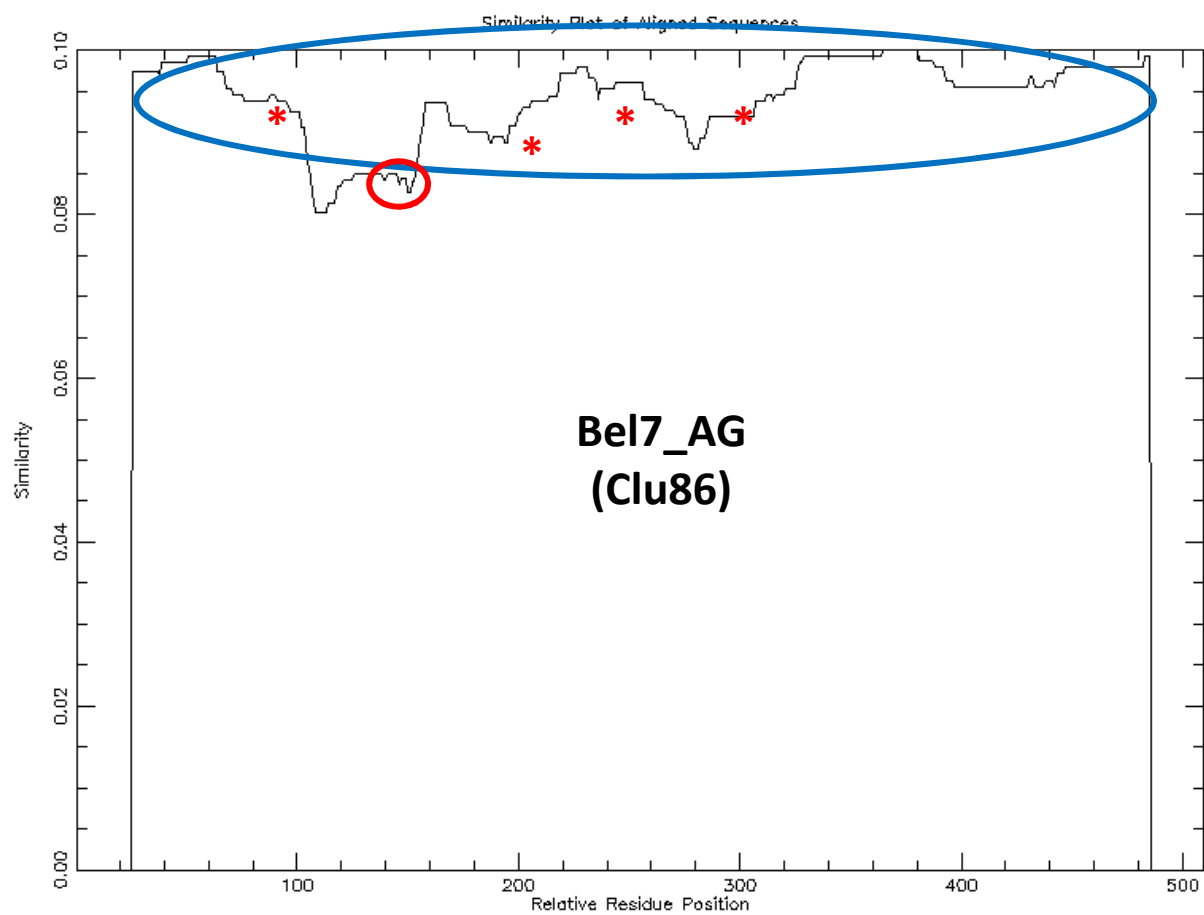

o)

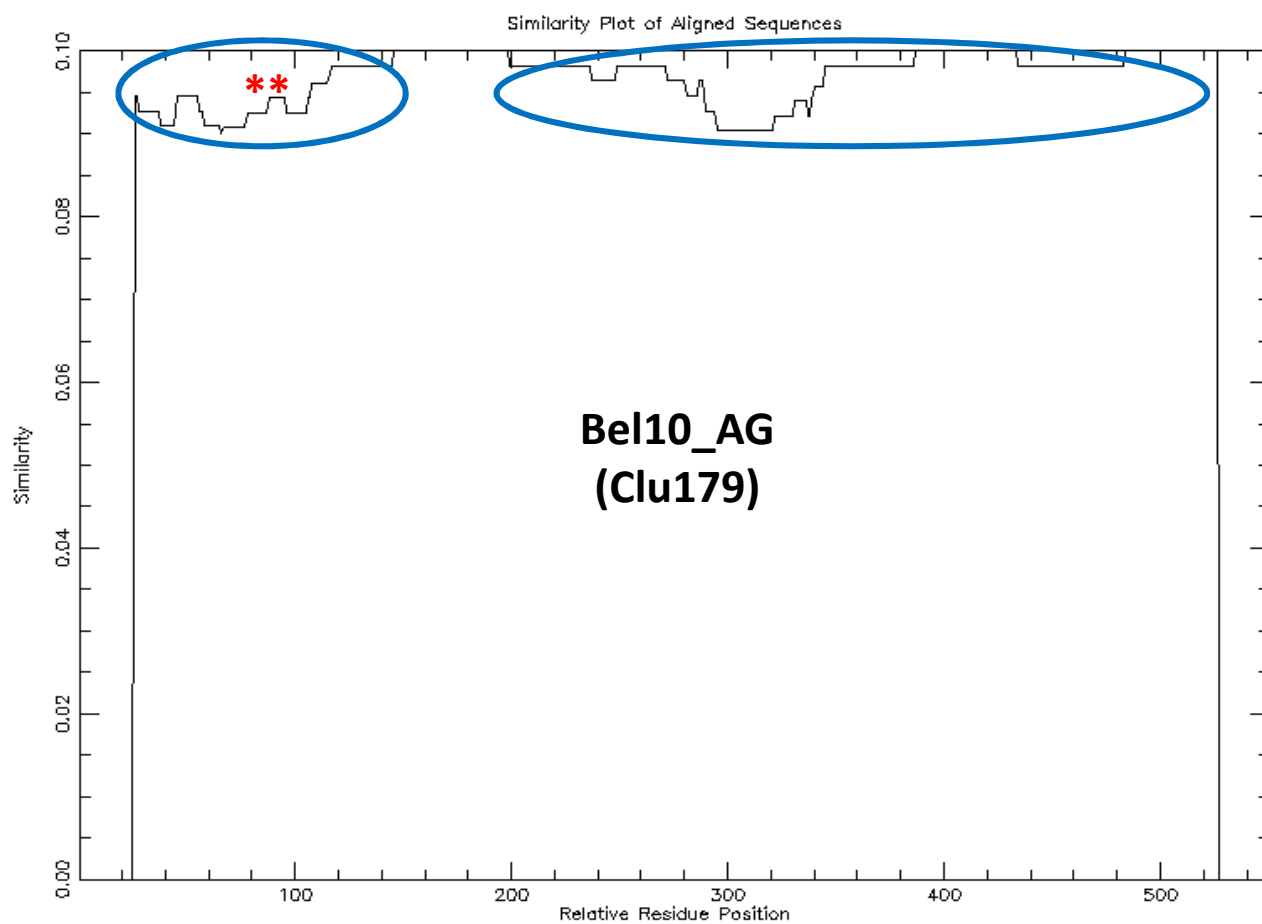

p)

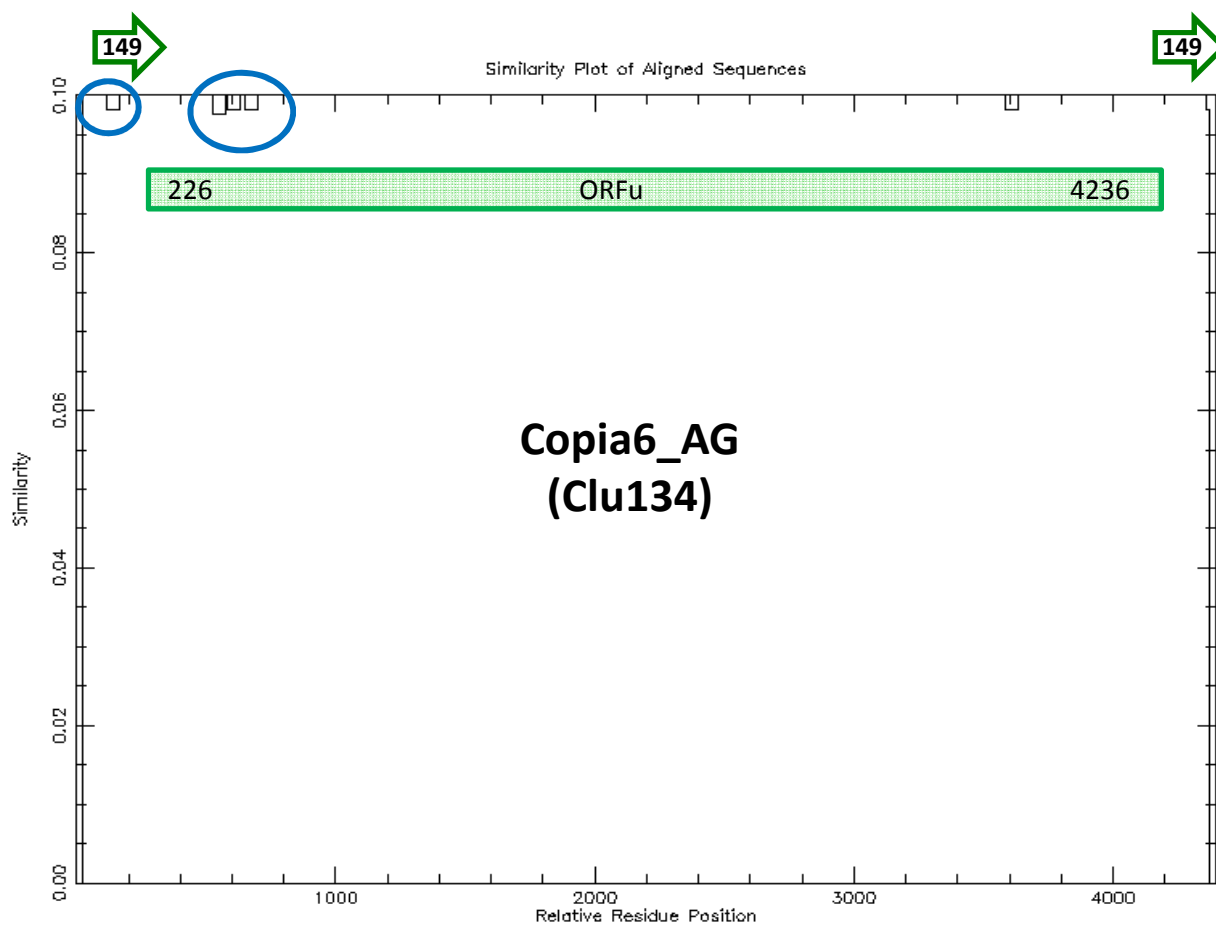

q)

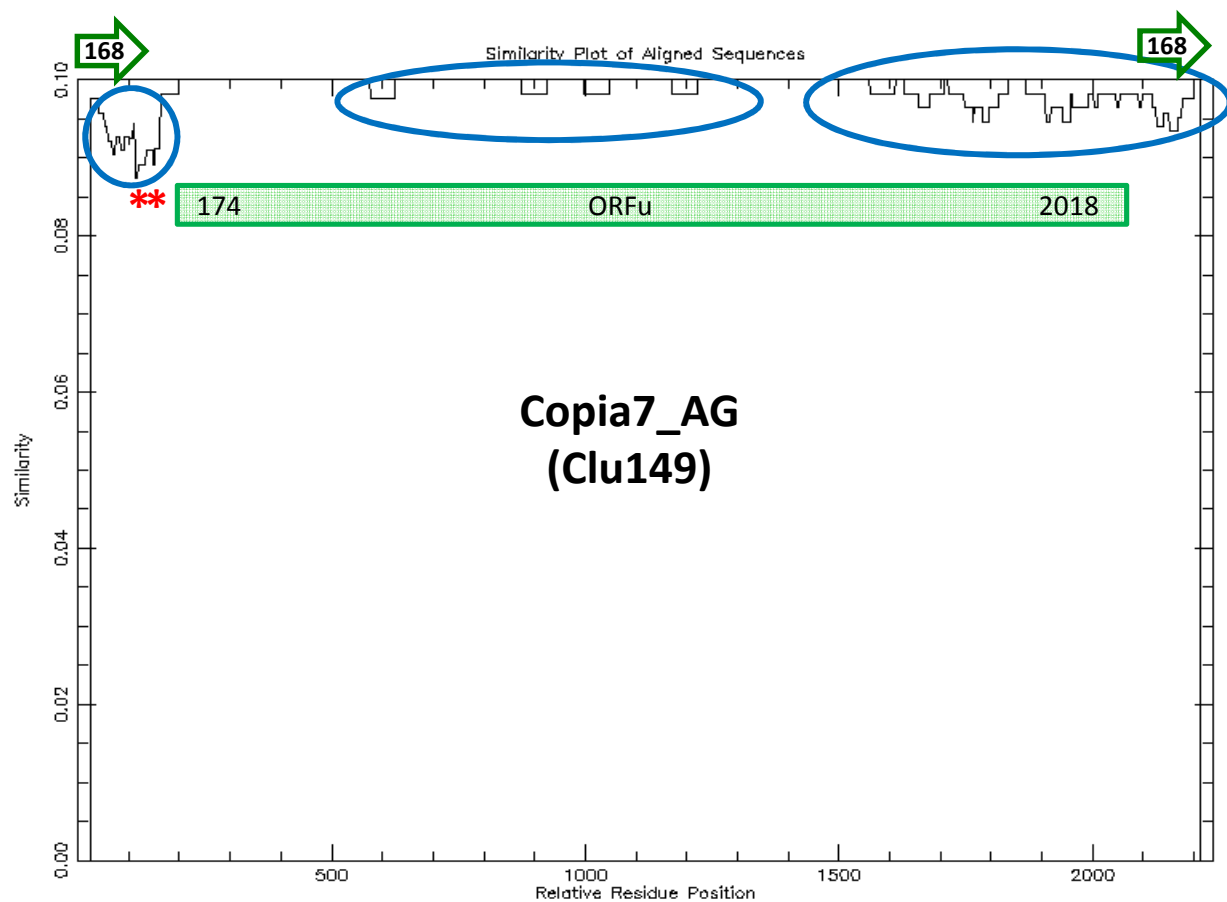

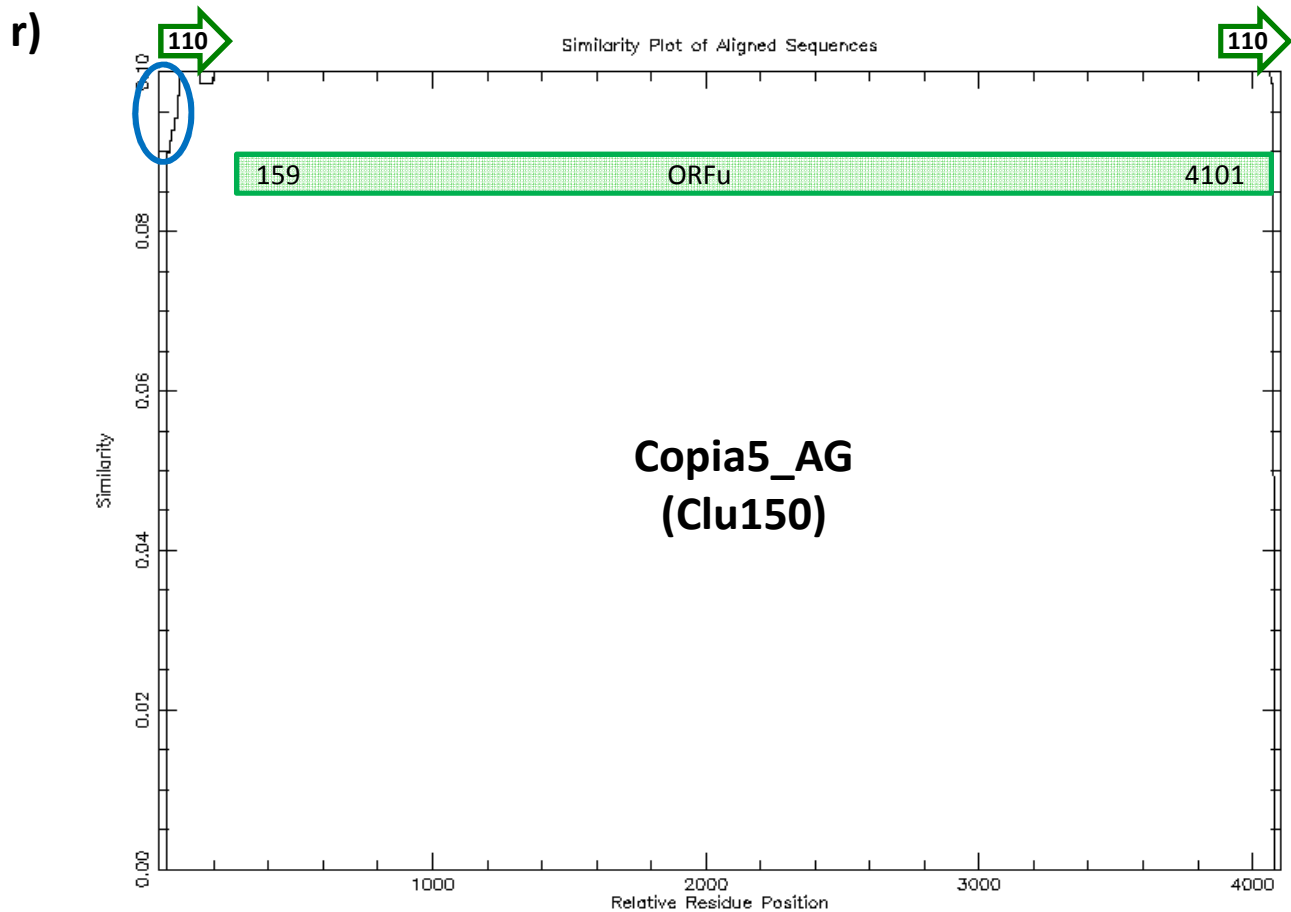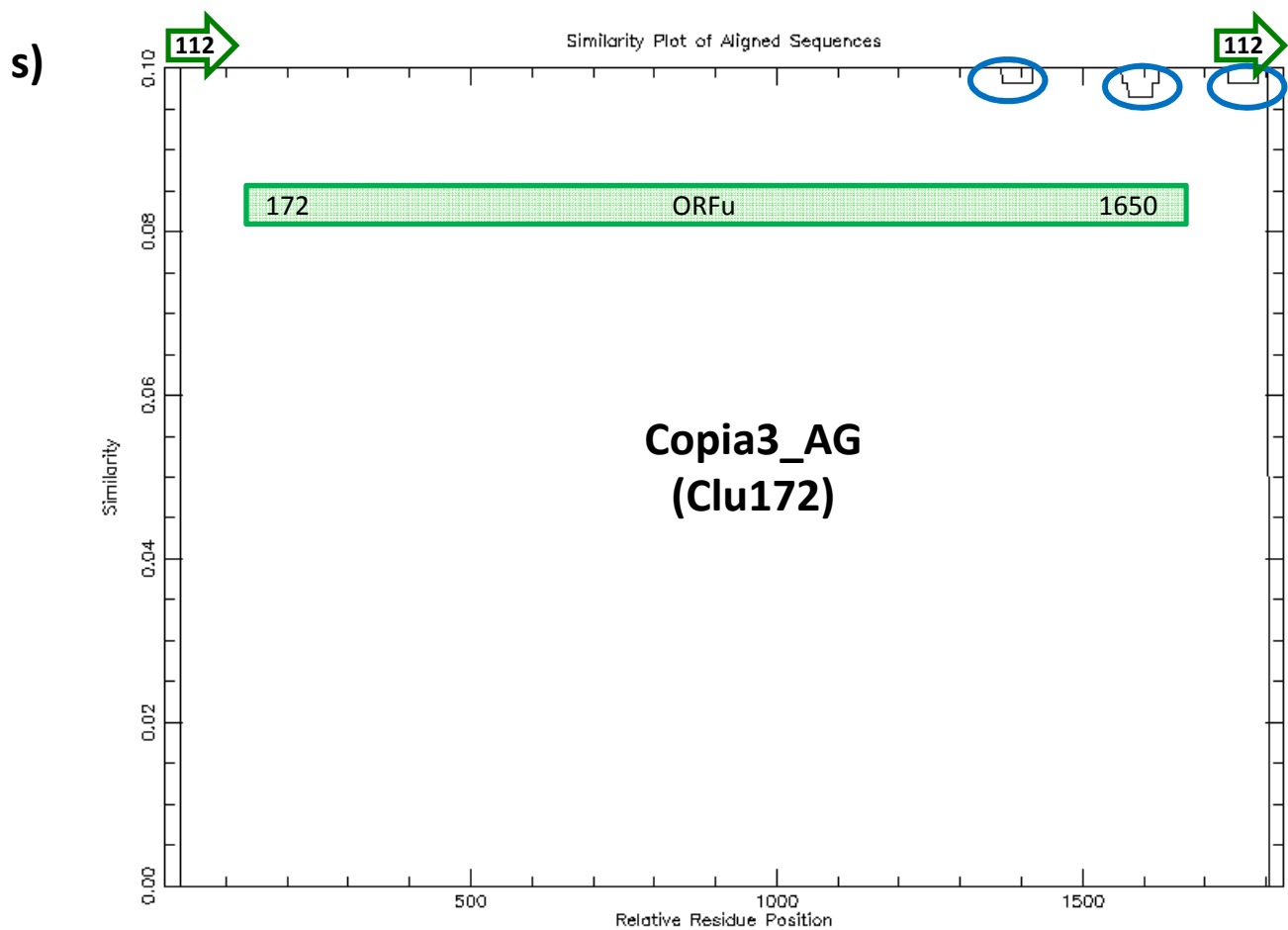

t)

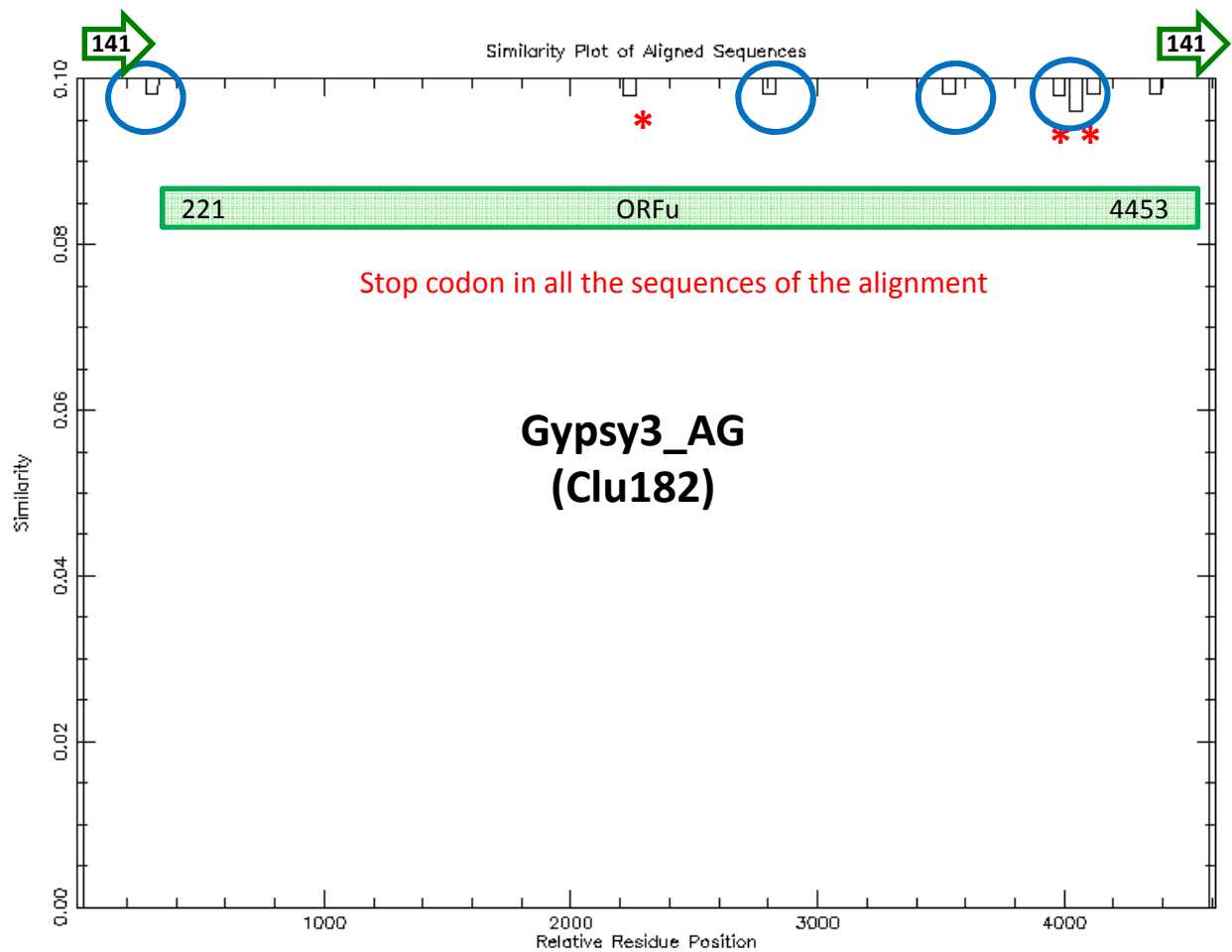

u)

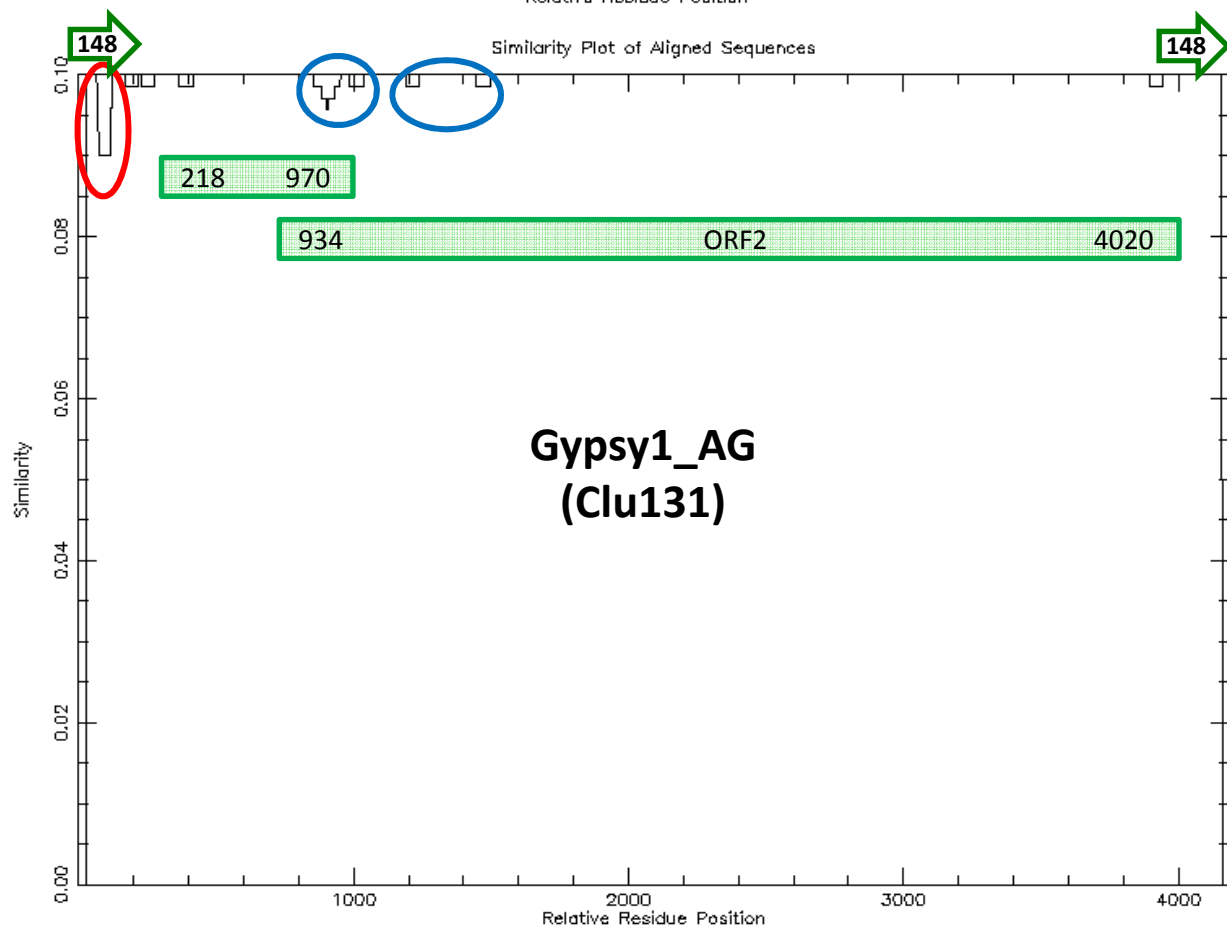

v)

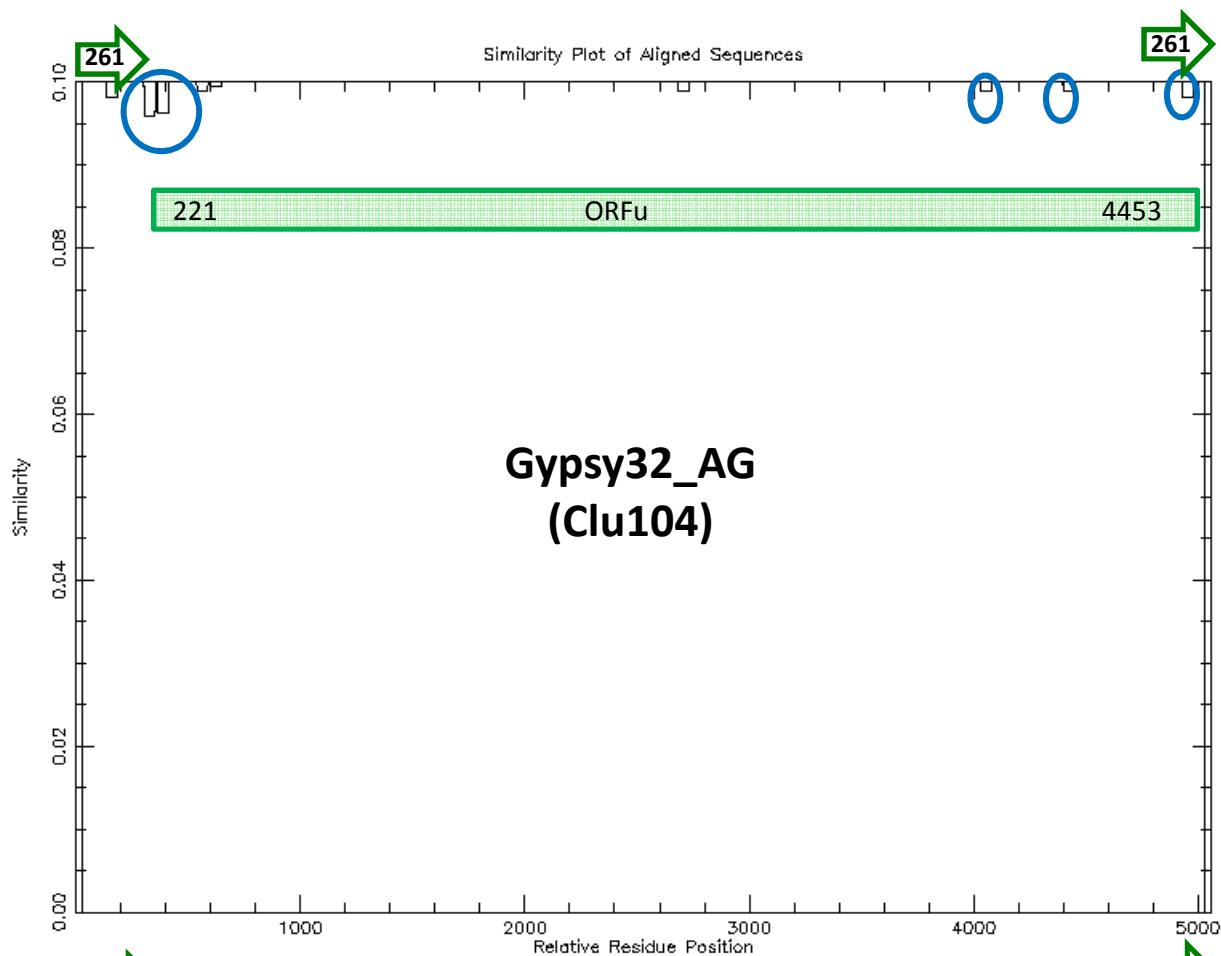

w)

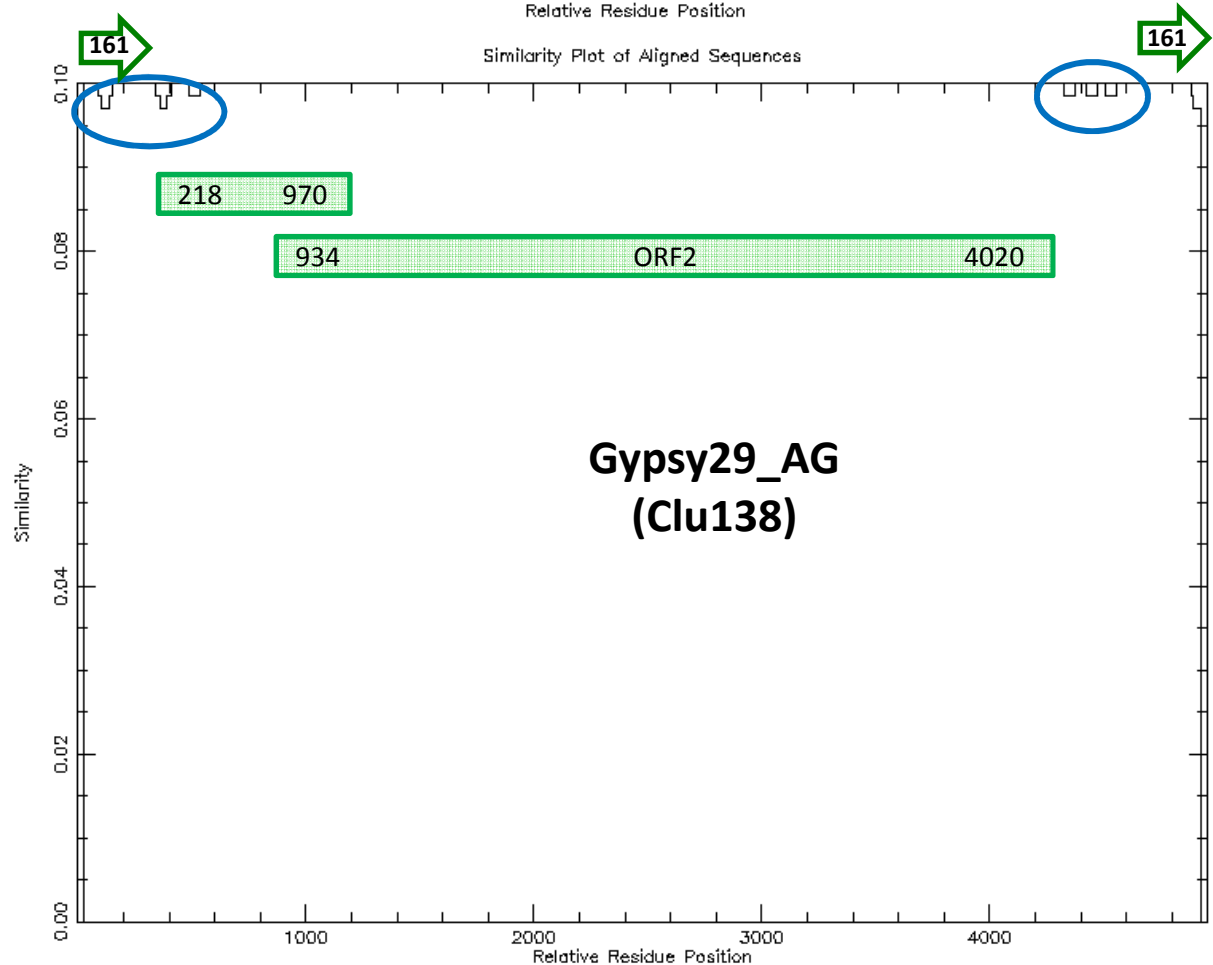

x)

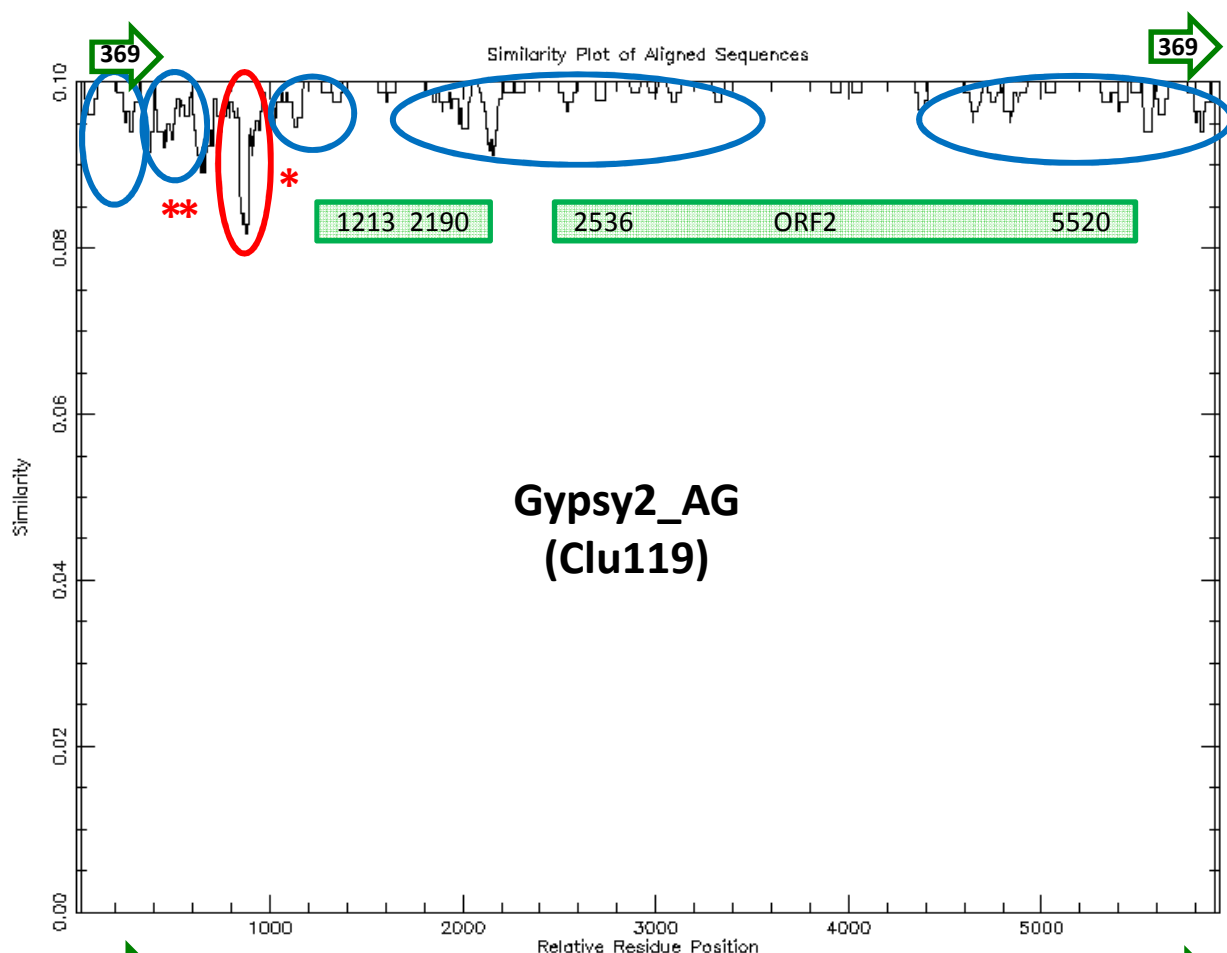

y)

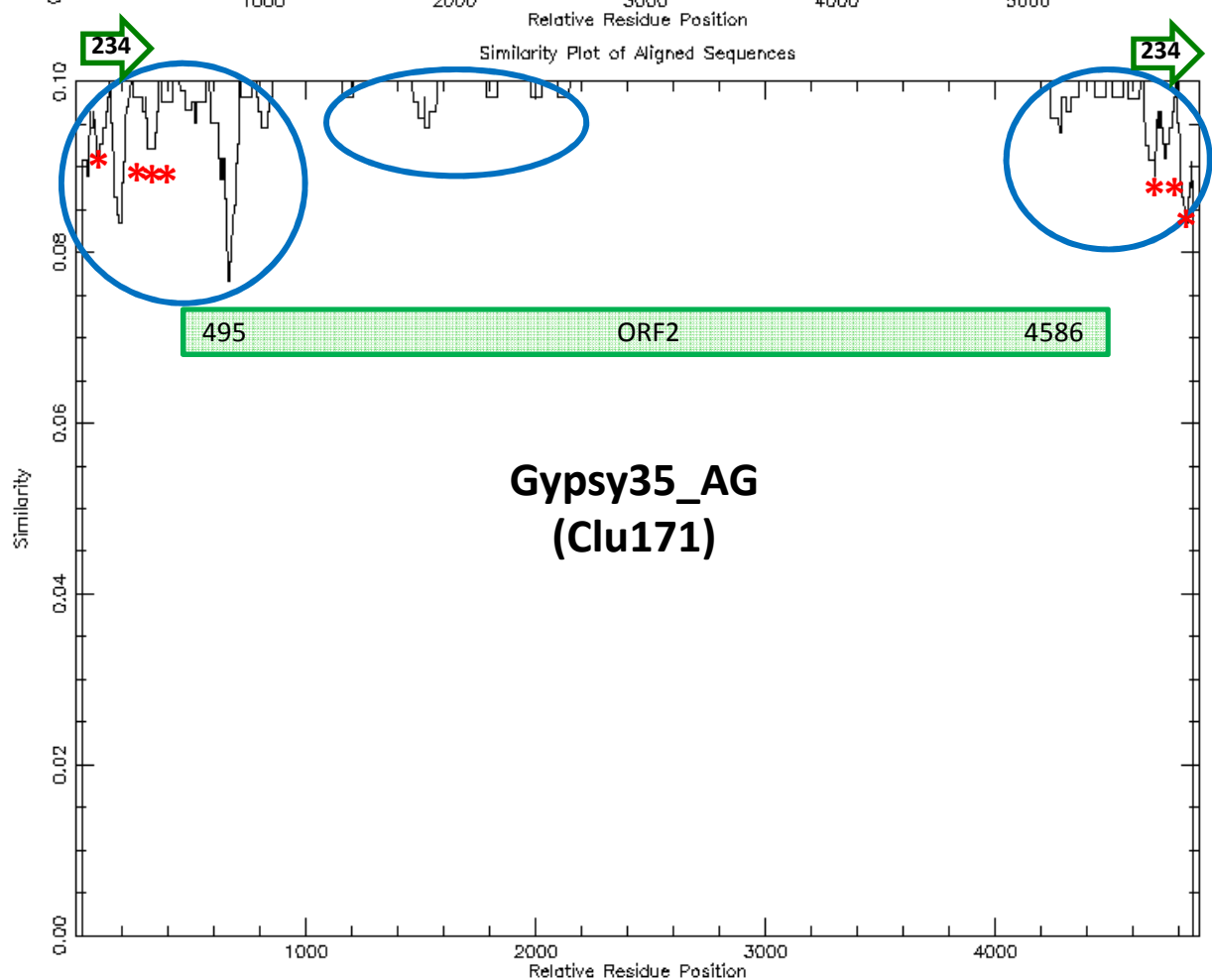

z)

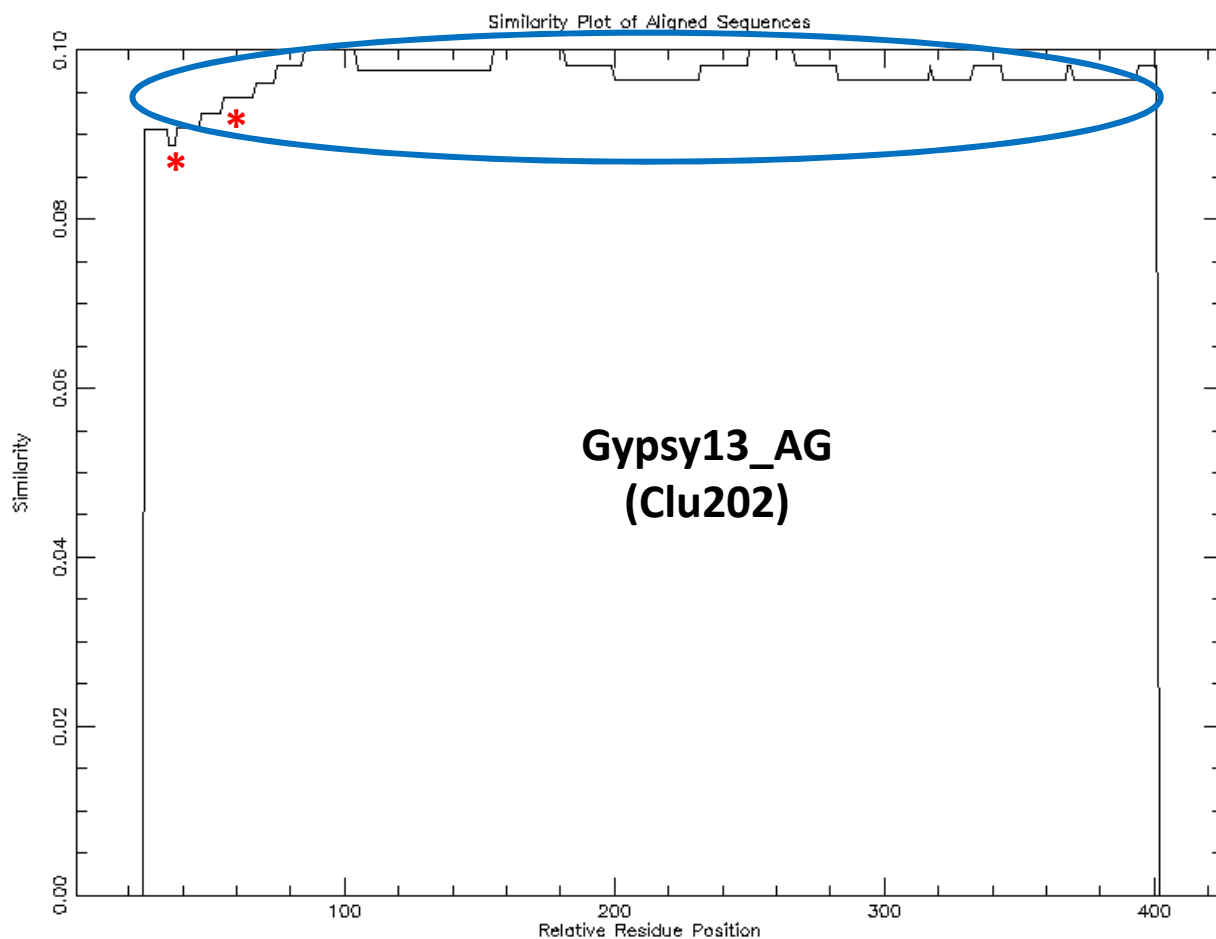

aa)

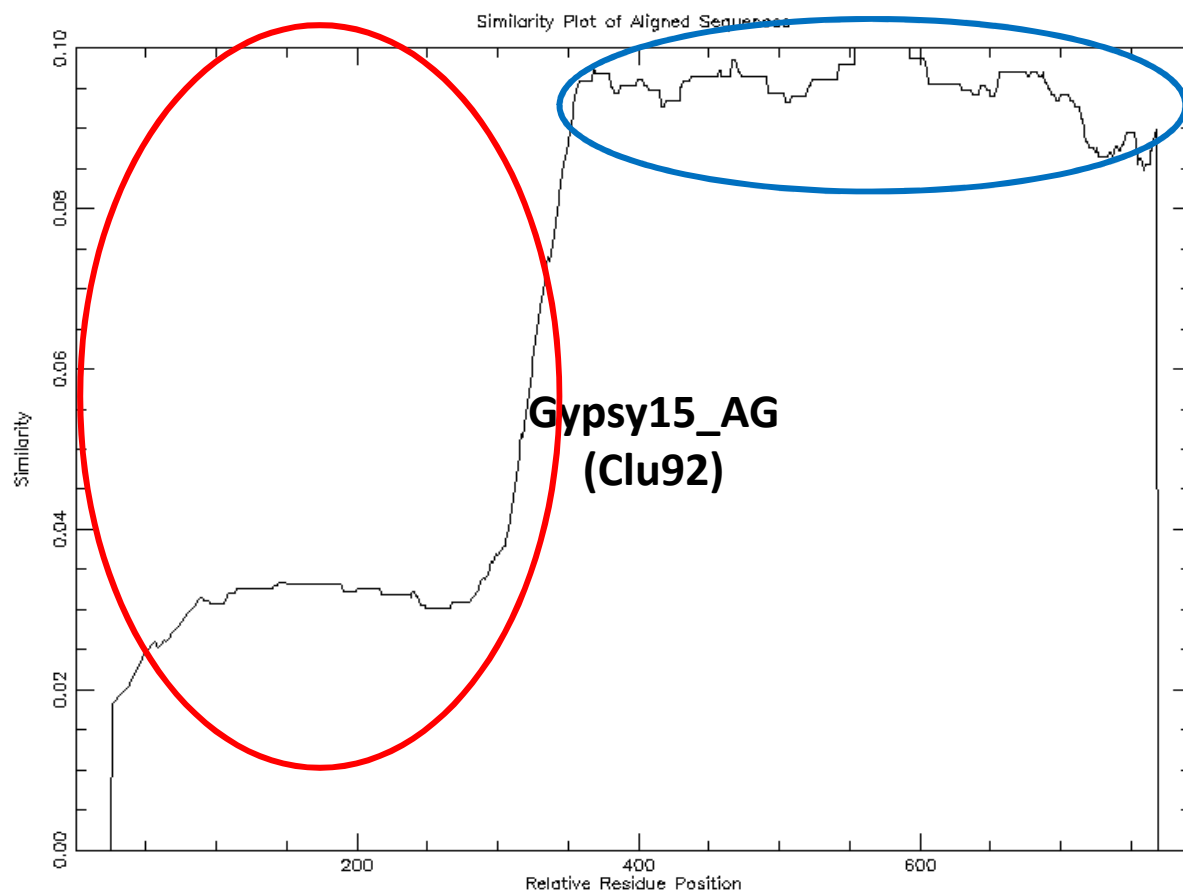

ab)

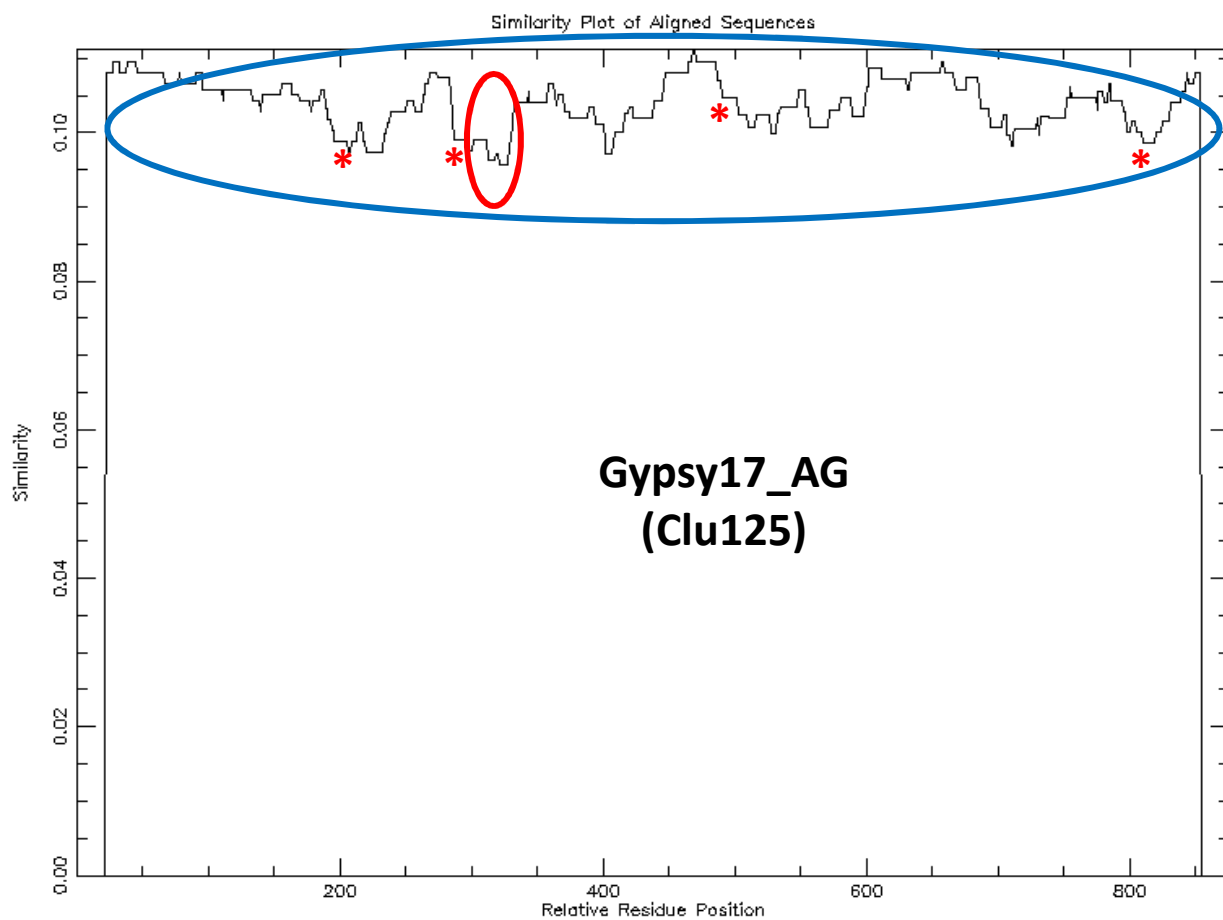

ac)

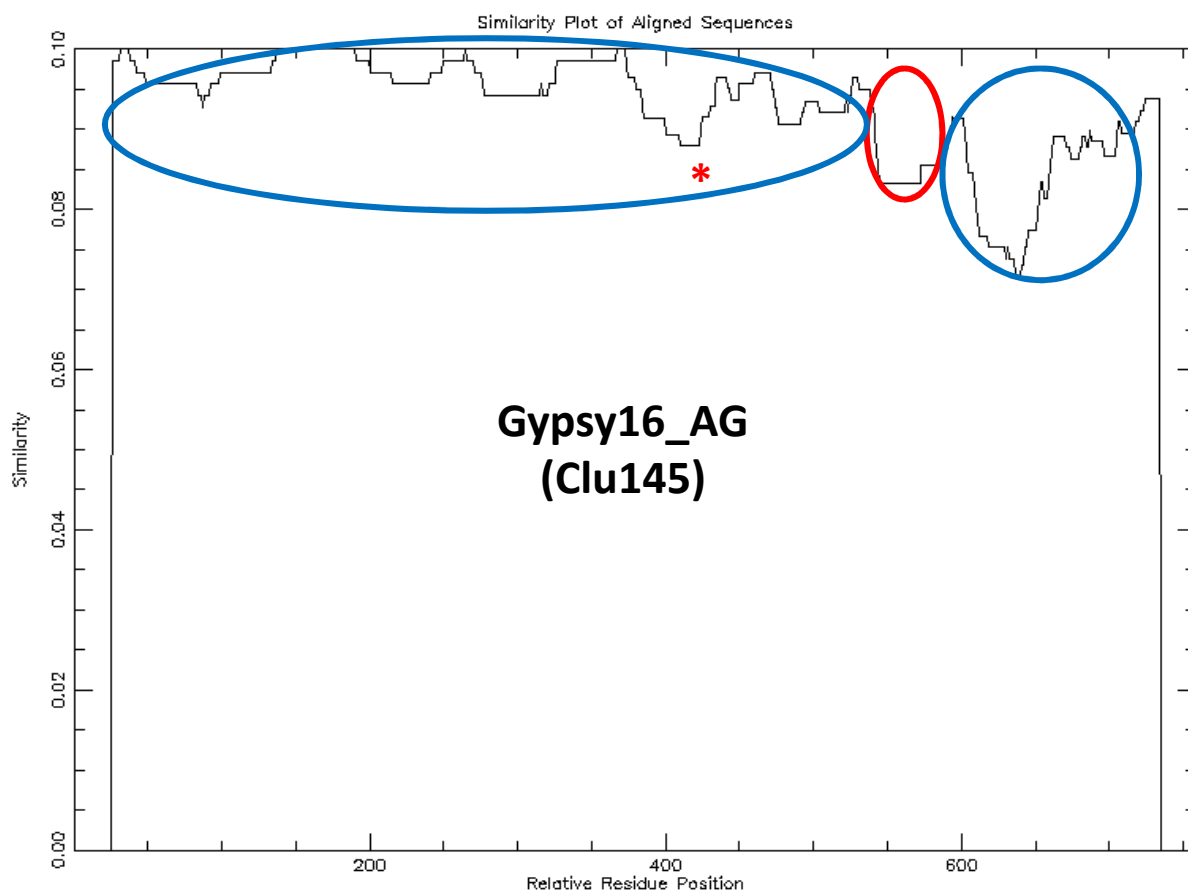

Supplement: Additional file 2 — Figure S1. Graphic representation of the deterioration profiles for the LTR superfamilies and families analyzed in this study. The graphics performed with Plotcon (http://emboss.bioinformatics.nl/cgi-bin/emboss/plotcon) represent the similarity of the sequences along multiple alignments performed for several families belonging to the LTR superfamilies: Pao-Bel: (a–l) correspond to families of full-length sequences, (m–o) correspond to families of Solo-LTR sequences; Copia superfamily (p–s) correspond to families of full-length sequences; and the Gypsy superfamily: (t–y) correspond to families of full-length sequences, (z–ac) correspond to families of Solo-LTRs. Each family was aligned to the respective reference sequence as described in Repbase. The family name and the cluster number according to the AnoTExcel numbering (in parenthesis), are indicated in each graph as. Red circles are included to highlight regions where the differences between sequences are mainly due to indels; blue circles indicate regions where the differences are due to nucleotide substitutions; and green circles indicate regions where segmental deletions are present. Red asterisks are included to indicate single deletions and blue asterisks single insertions. The horizontal green bars represent the ORFs in the alignments, with numbers indicating their relative position to the first nucleotide of the alignment. Green arrows at the top of each graph indicated the relative position of the LTRs; the numbers within the arrows indicate the length of the LTRs. The X axis for all plots refers to the relative residue position in each alignment and the Y axis to their similarity indicated as the pairwise scores that are taken from the specified similarity matrix (see Methods section for detailed information). (PDF 331 kb) [file 1471-2164-13-272-S2.pdf]

a)

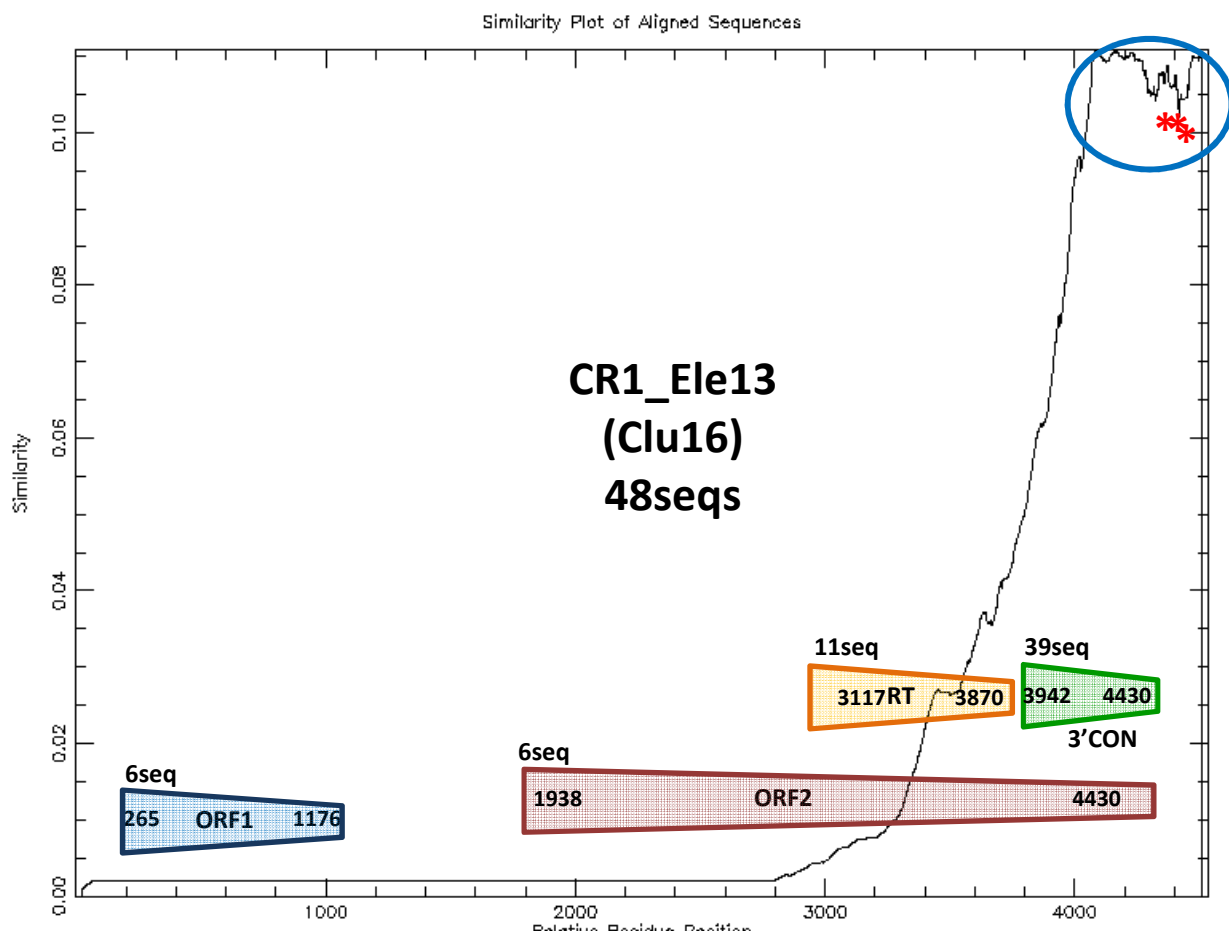

b)

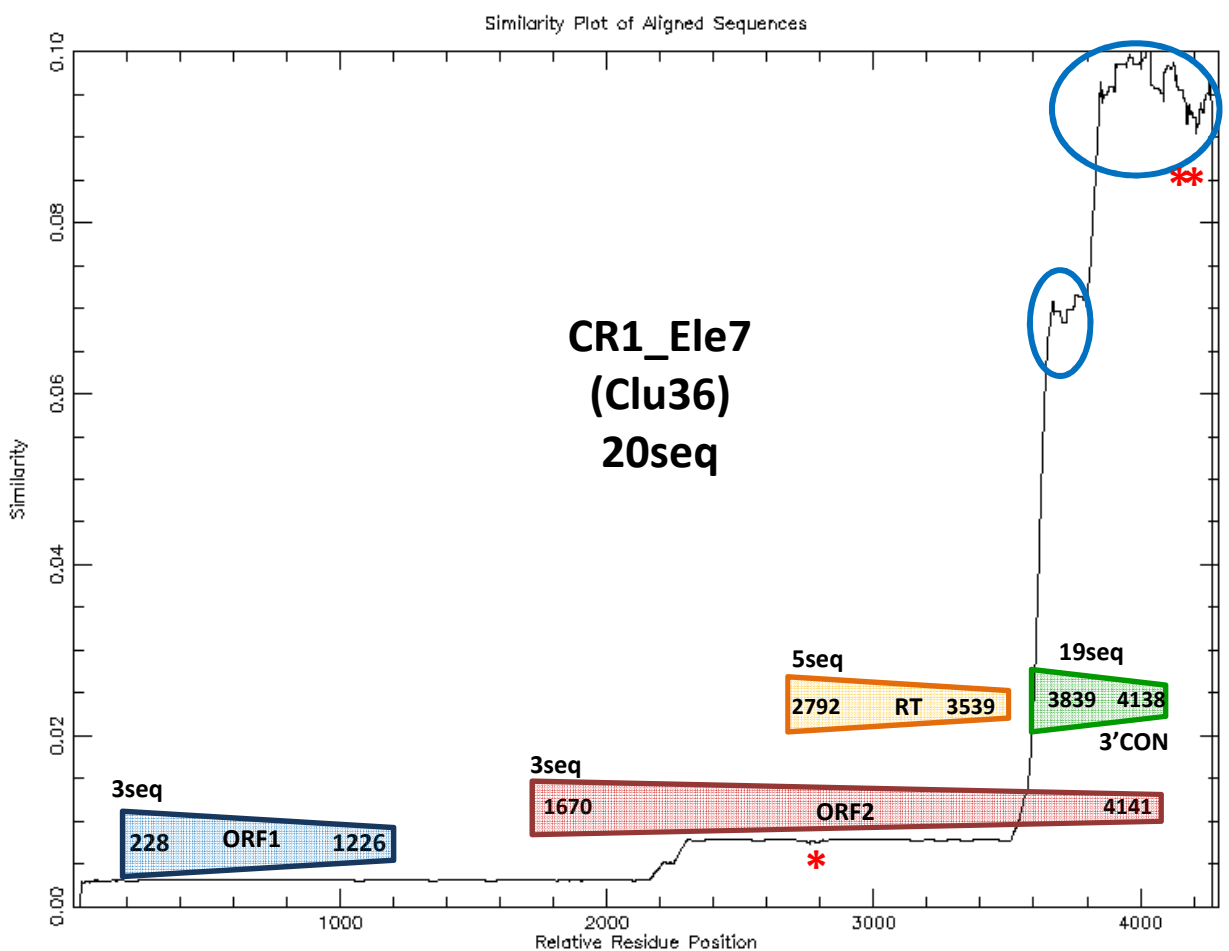

c)

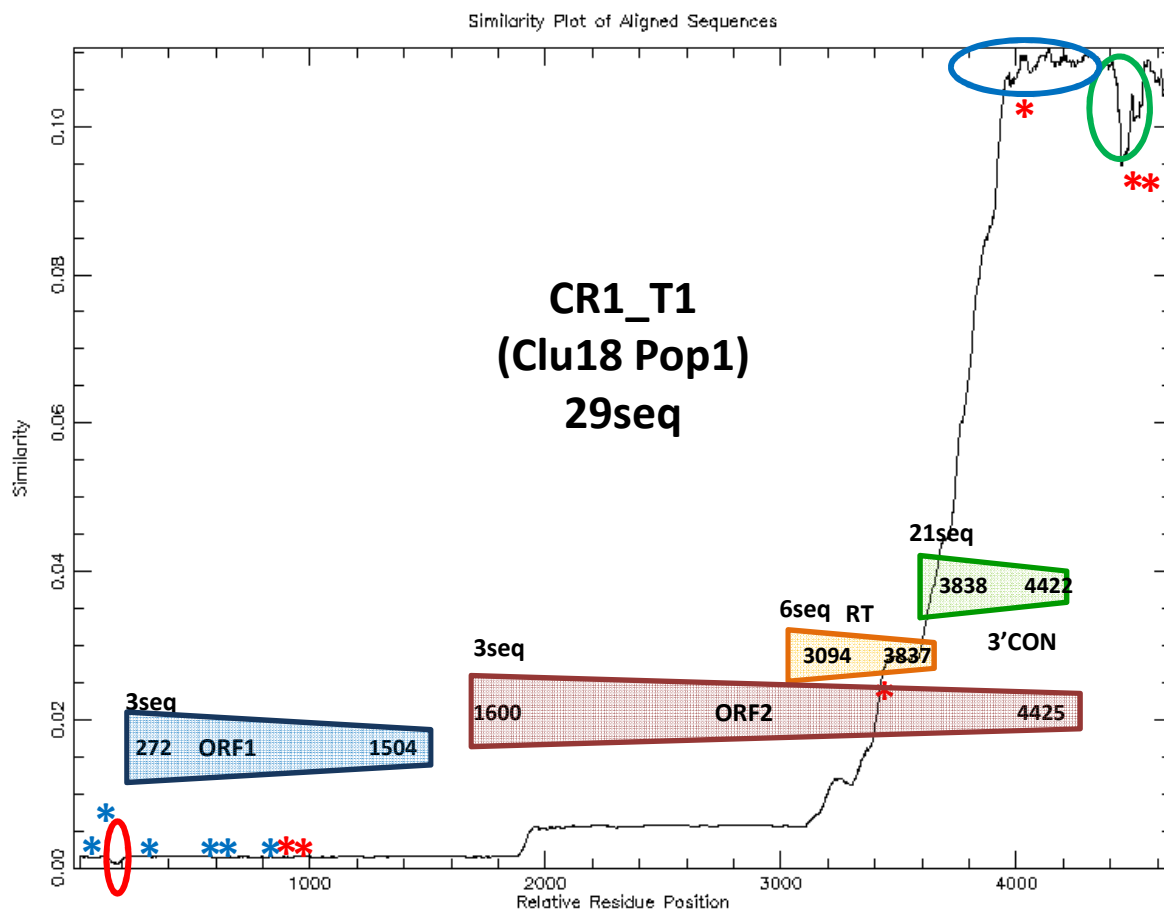

d)

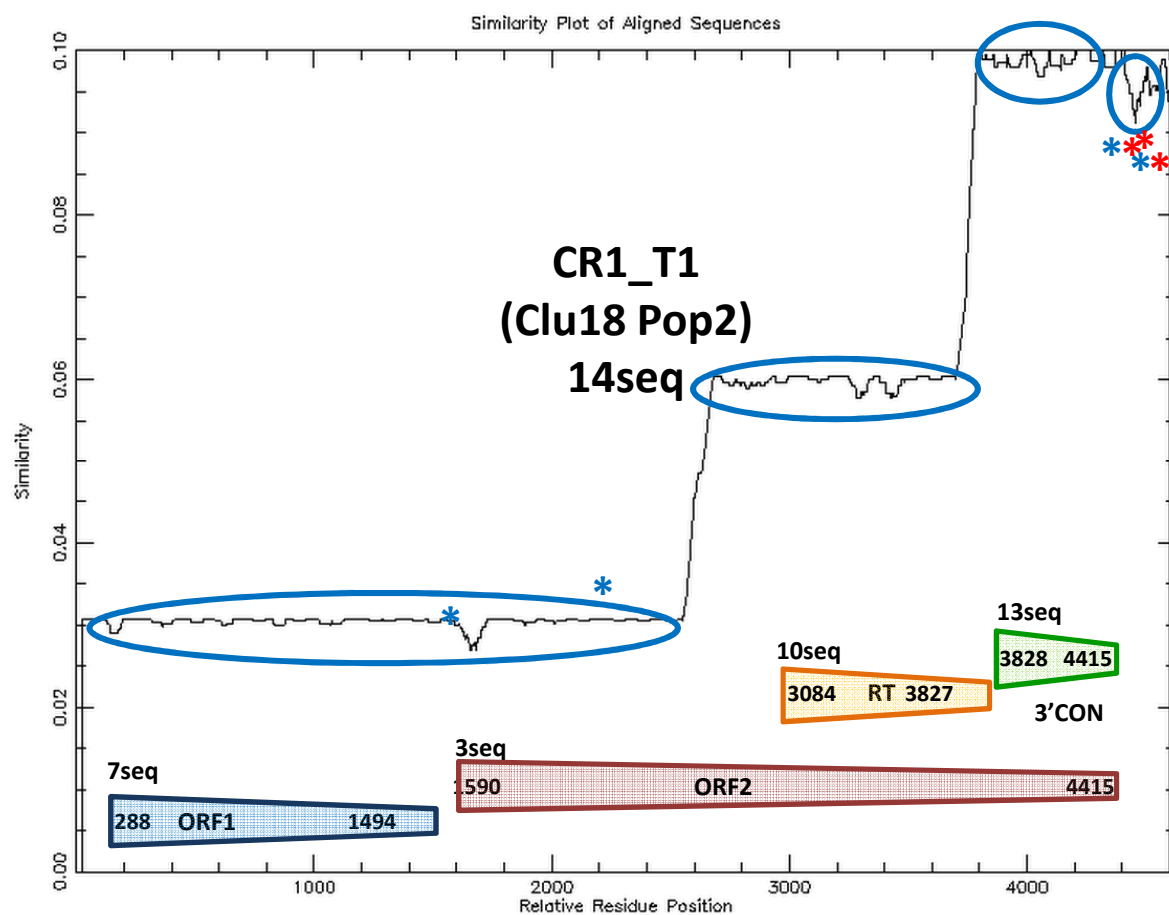

e)

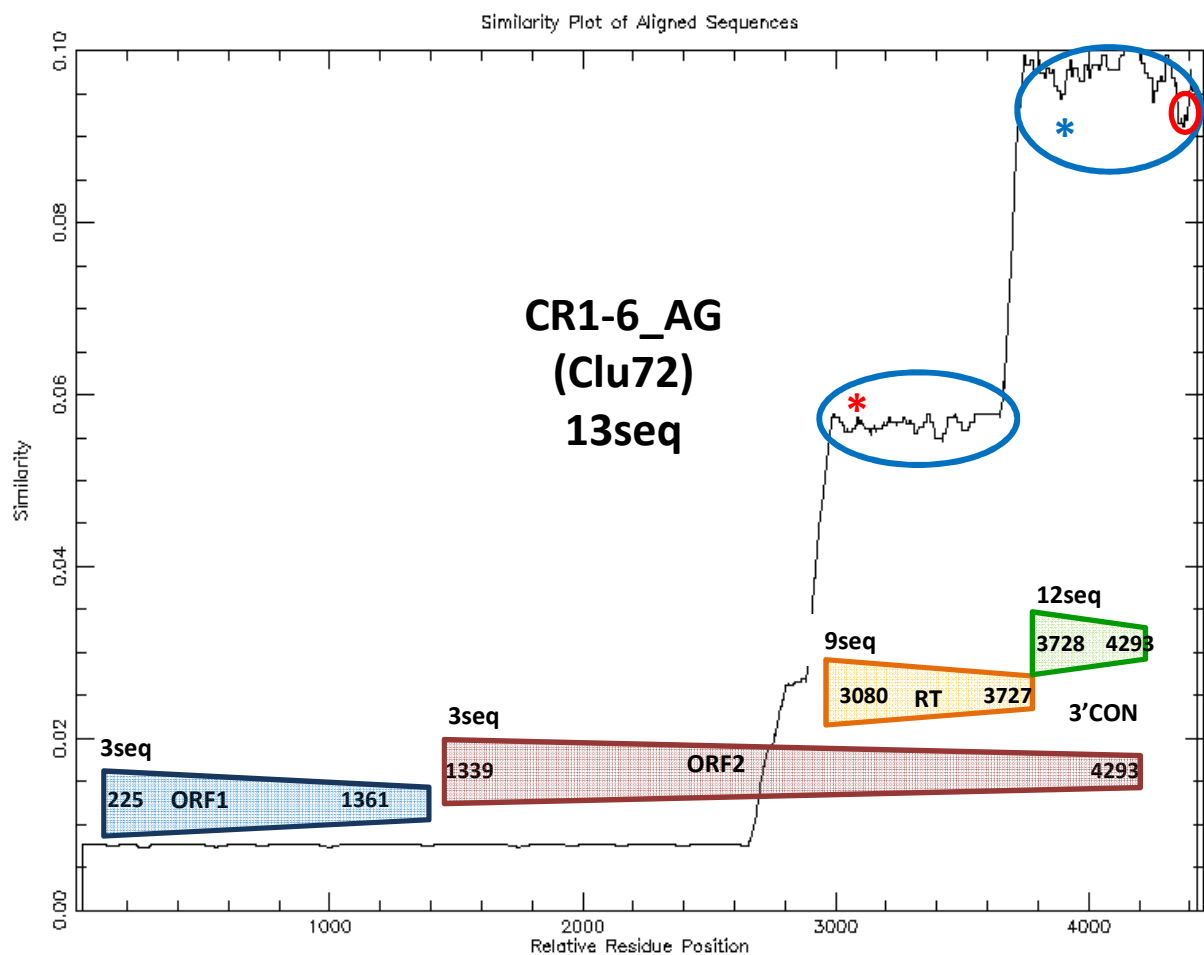

f)

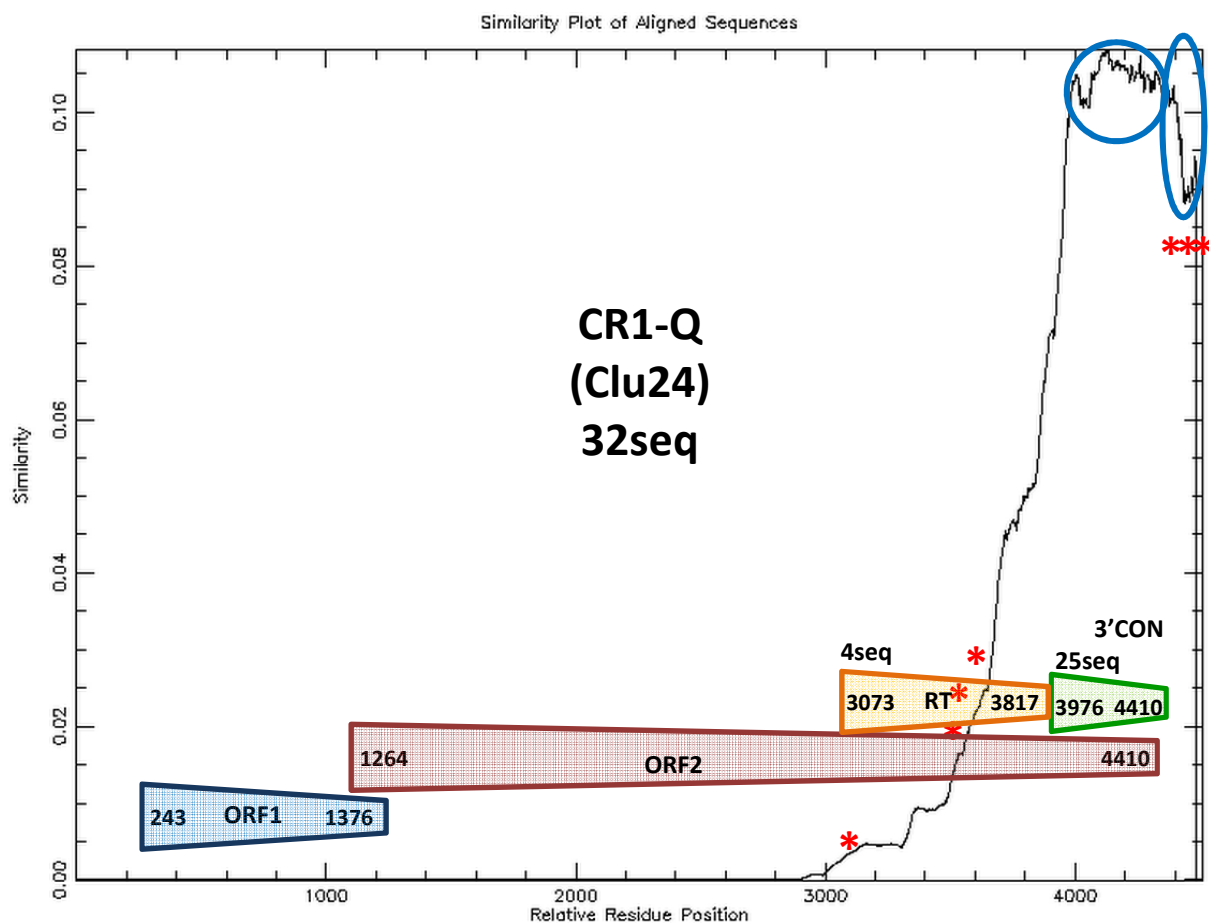

g)

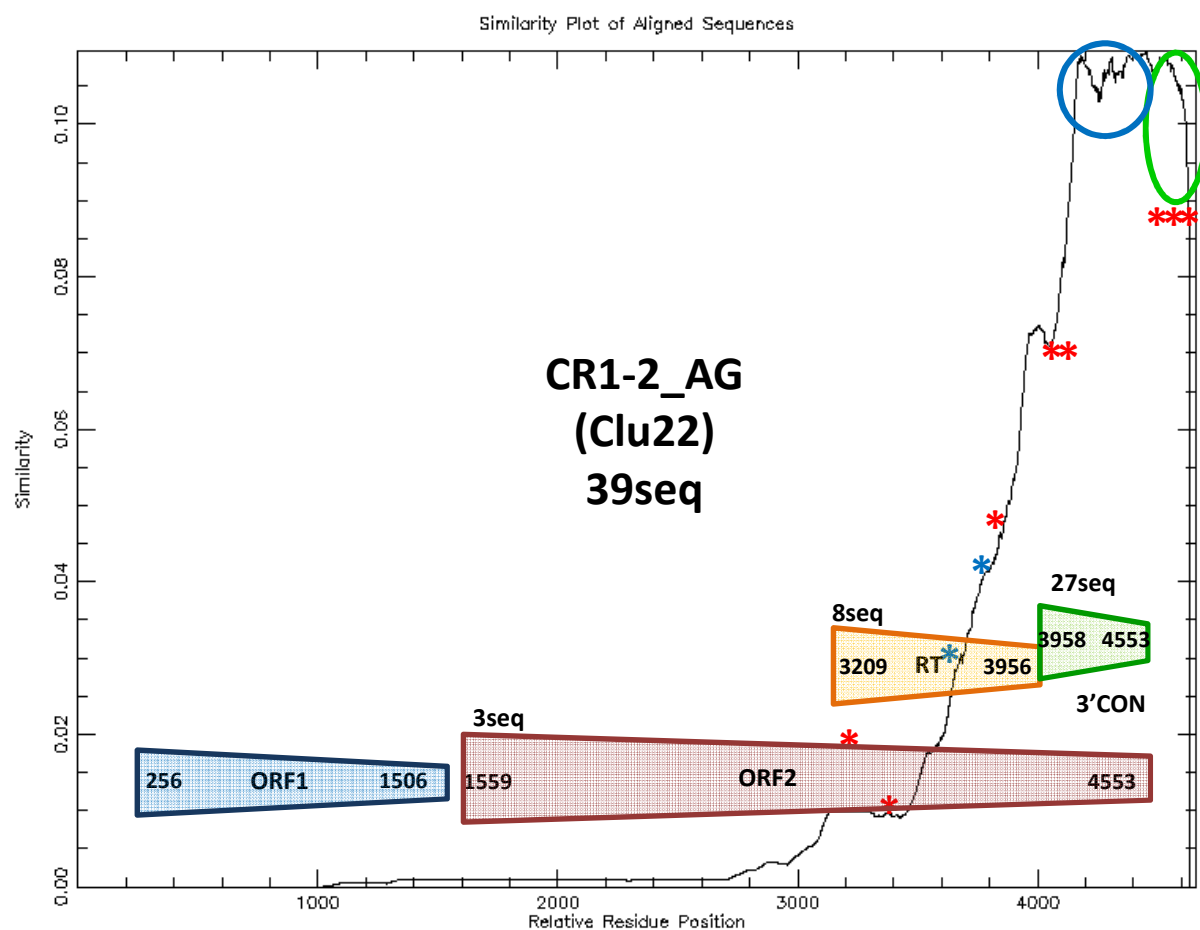

h)

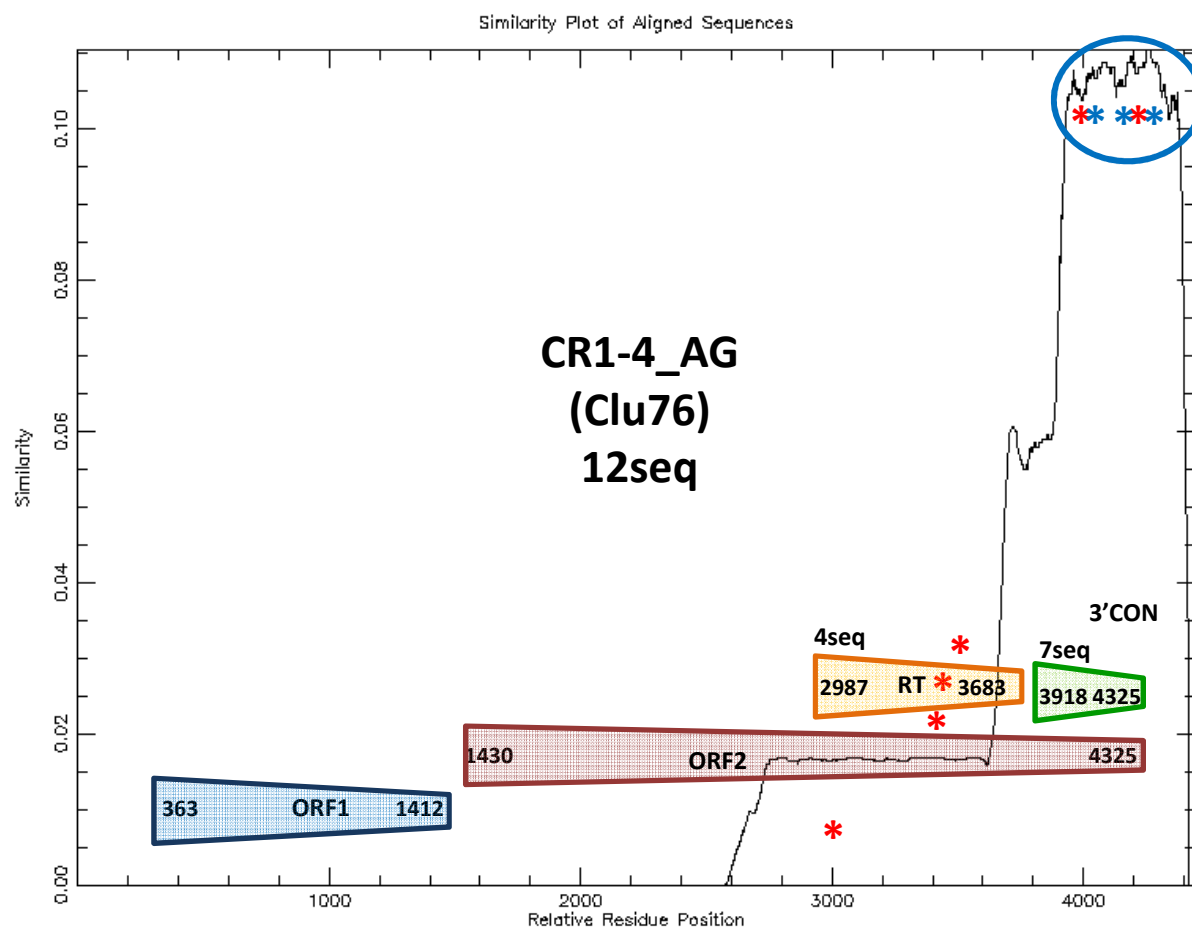

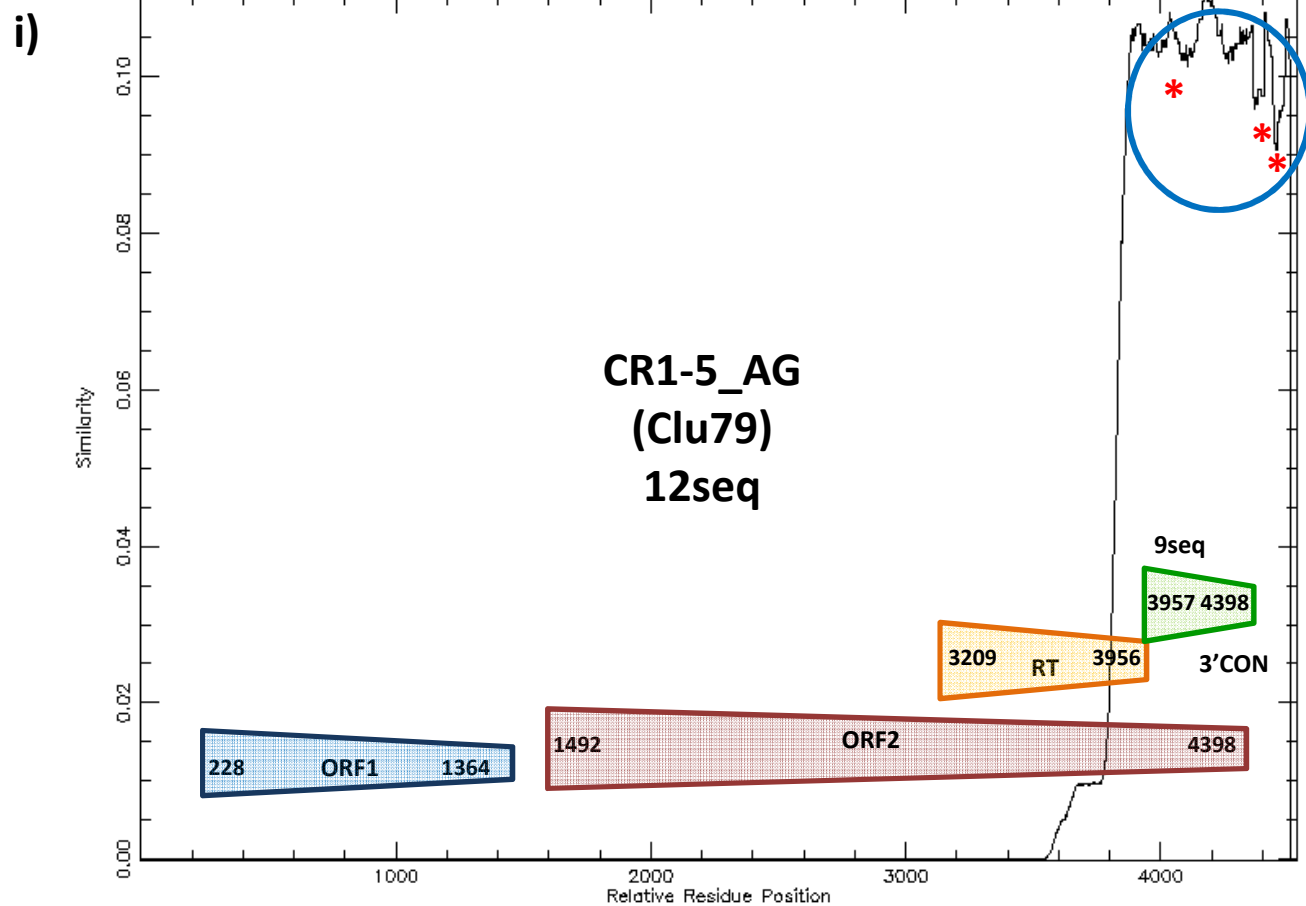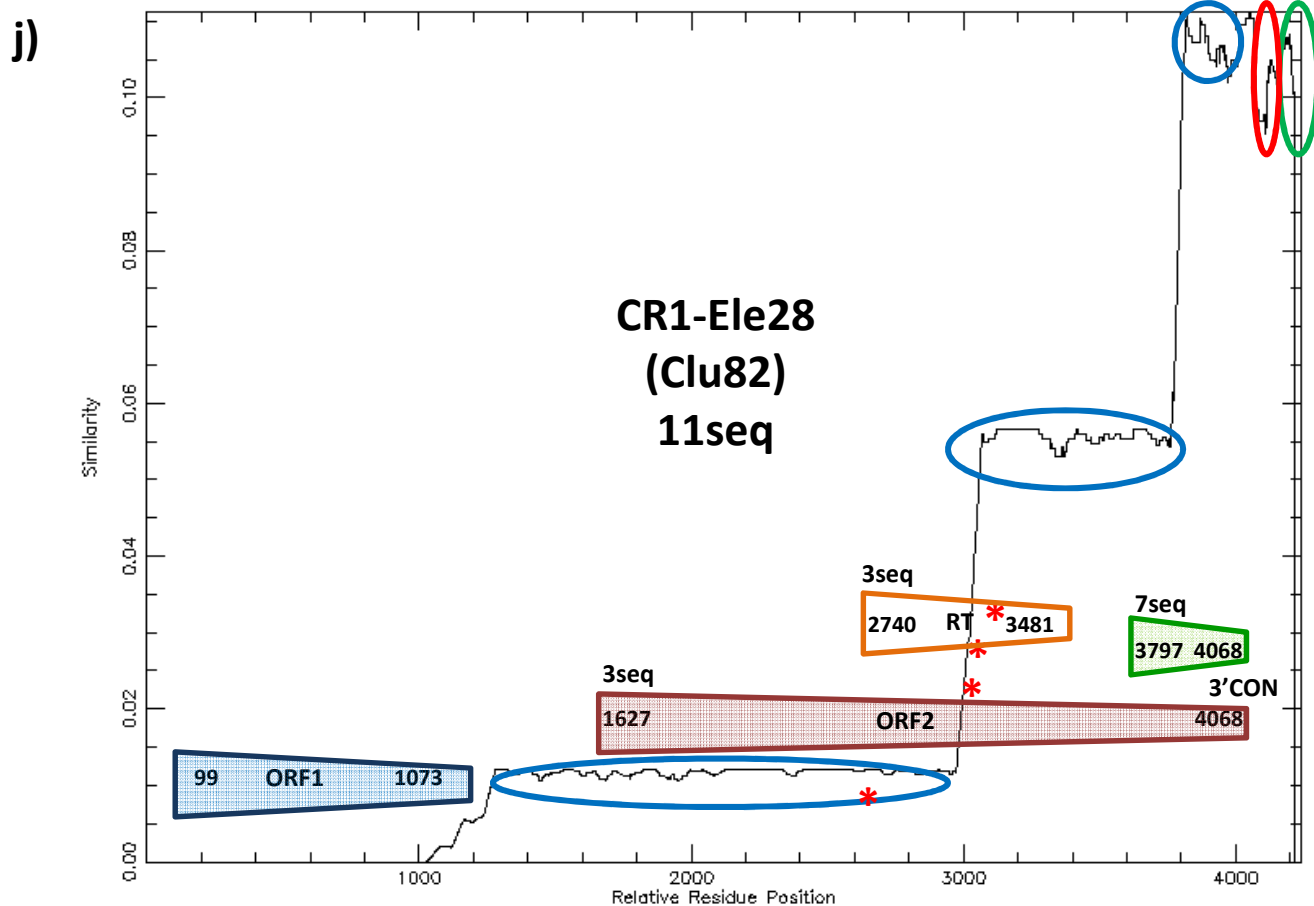

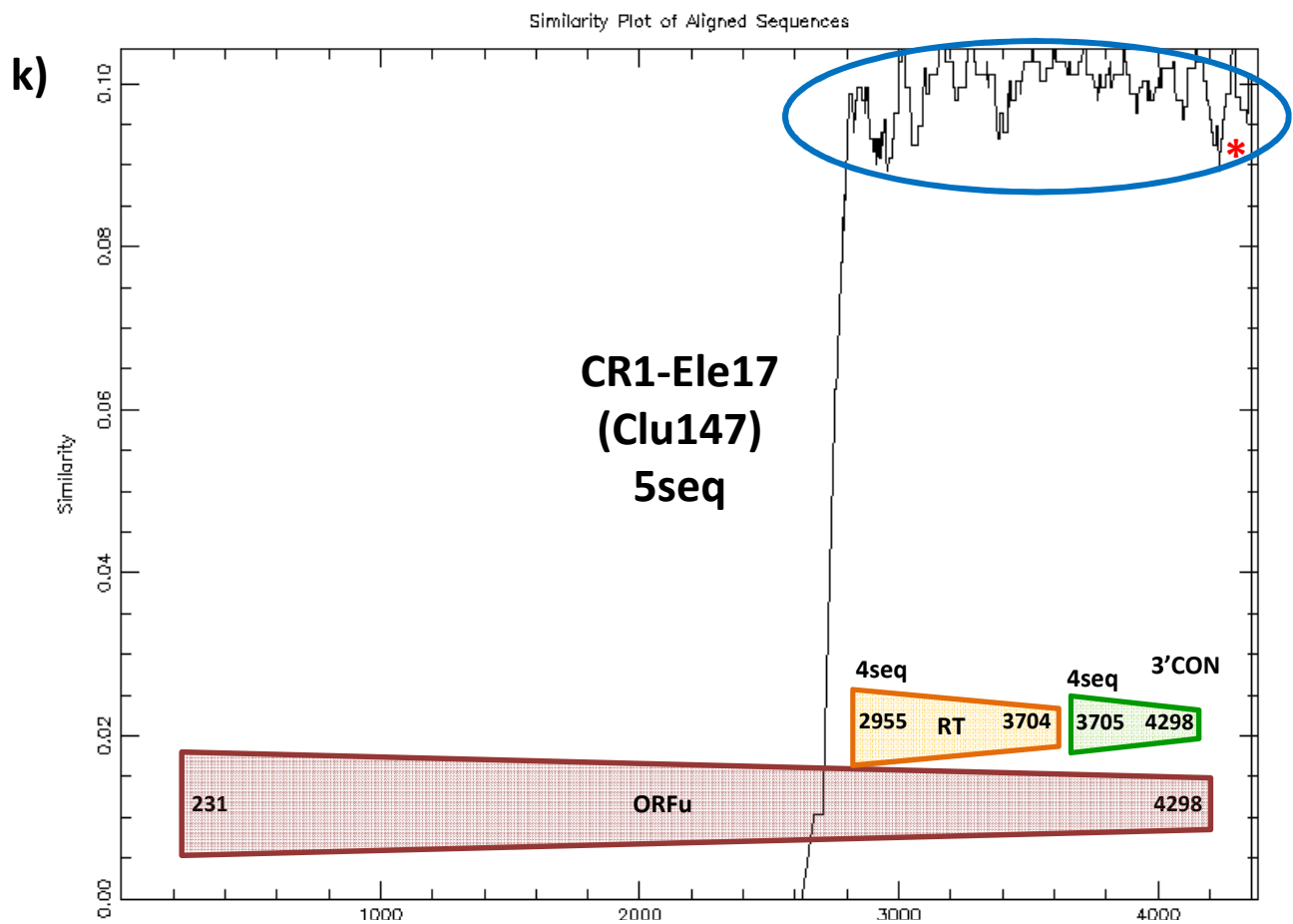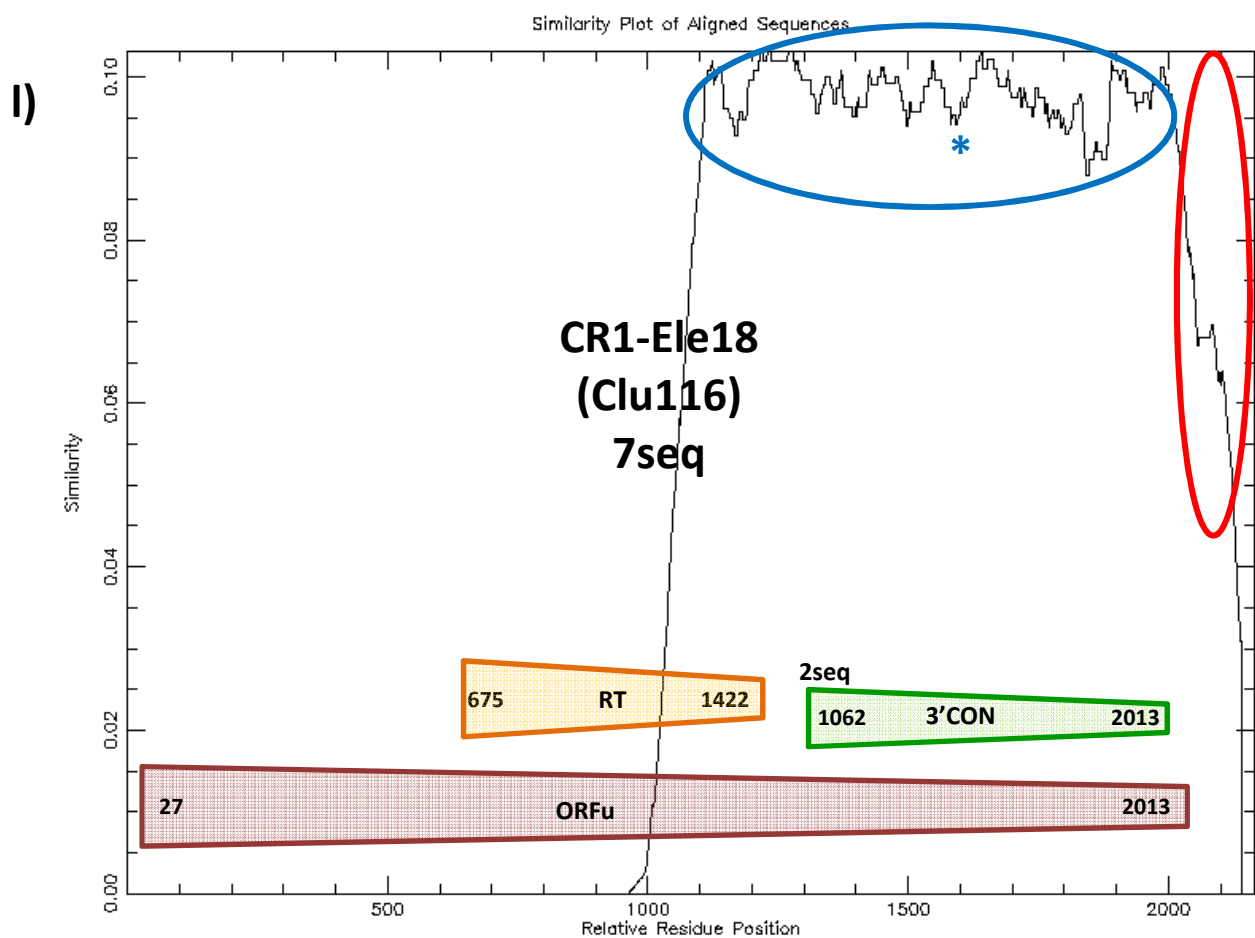

m)

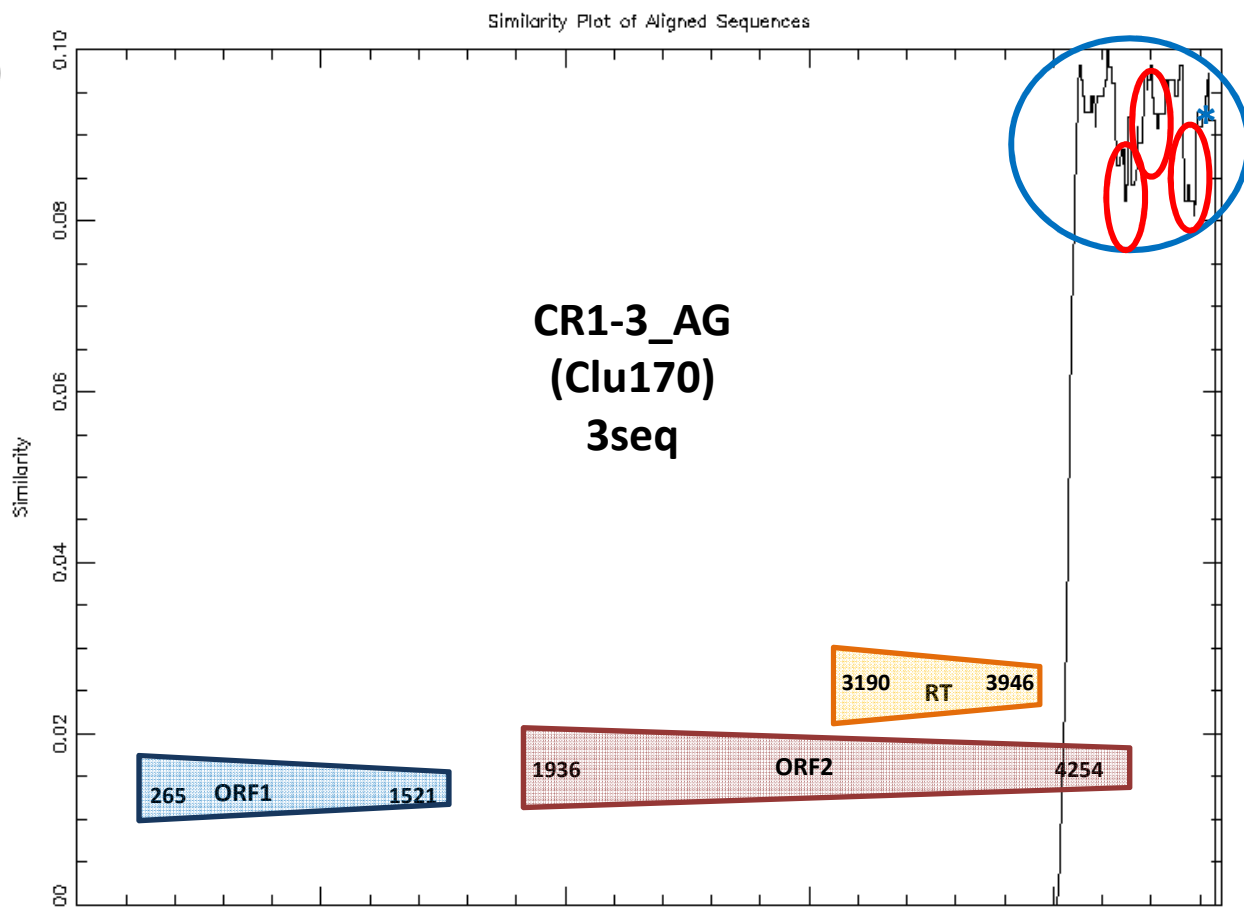

n)

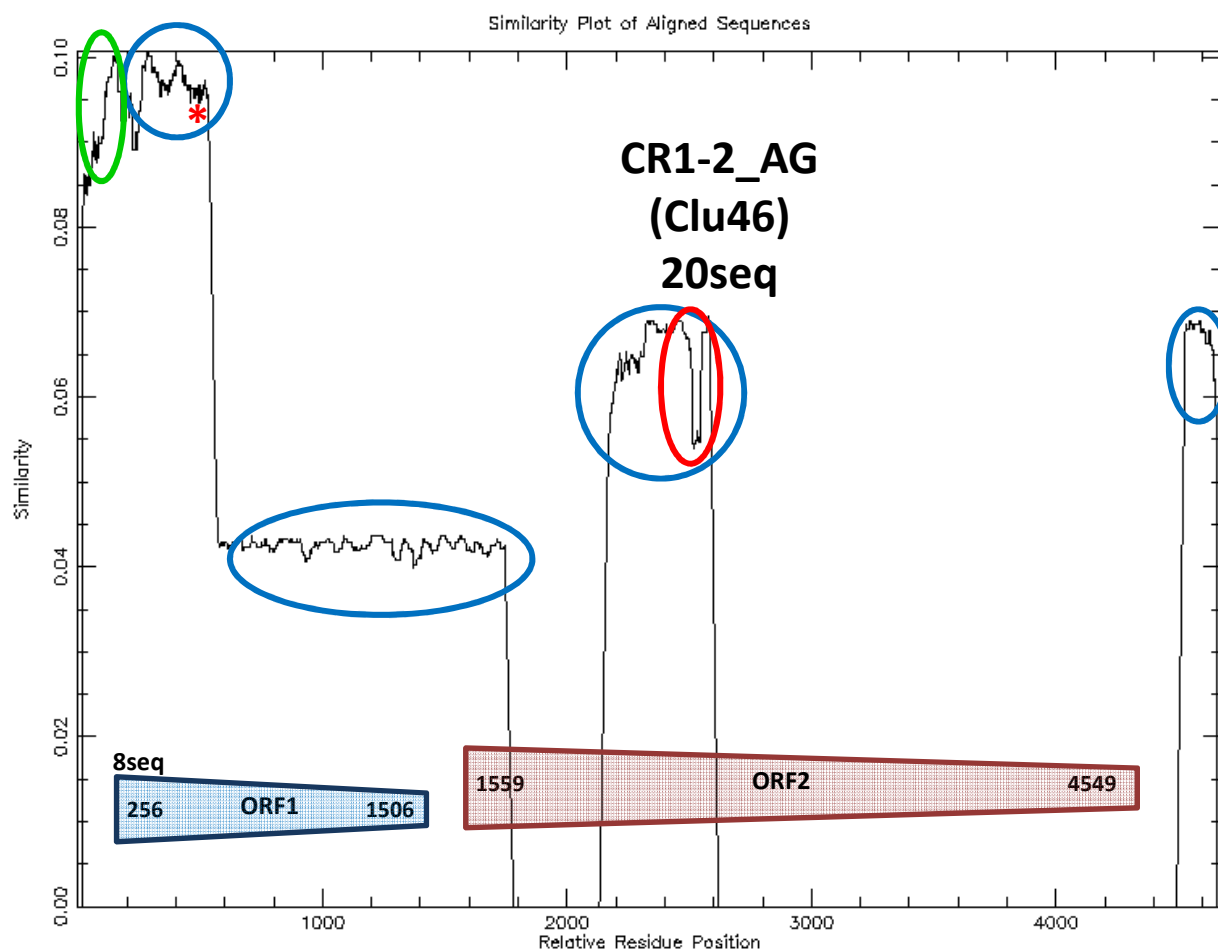

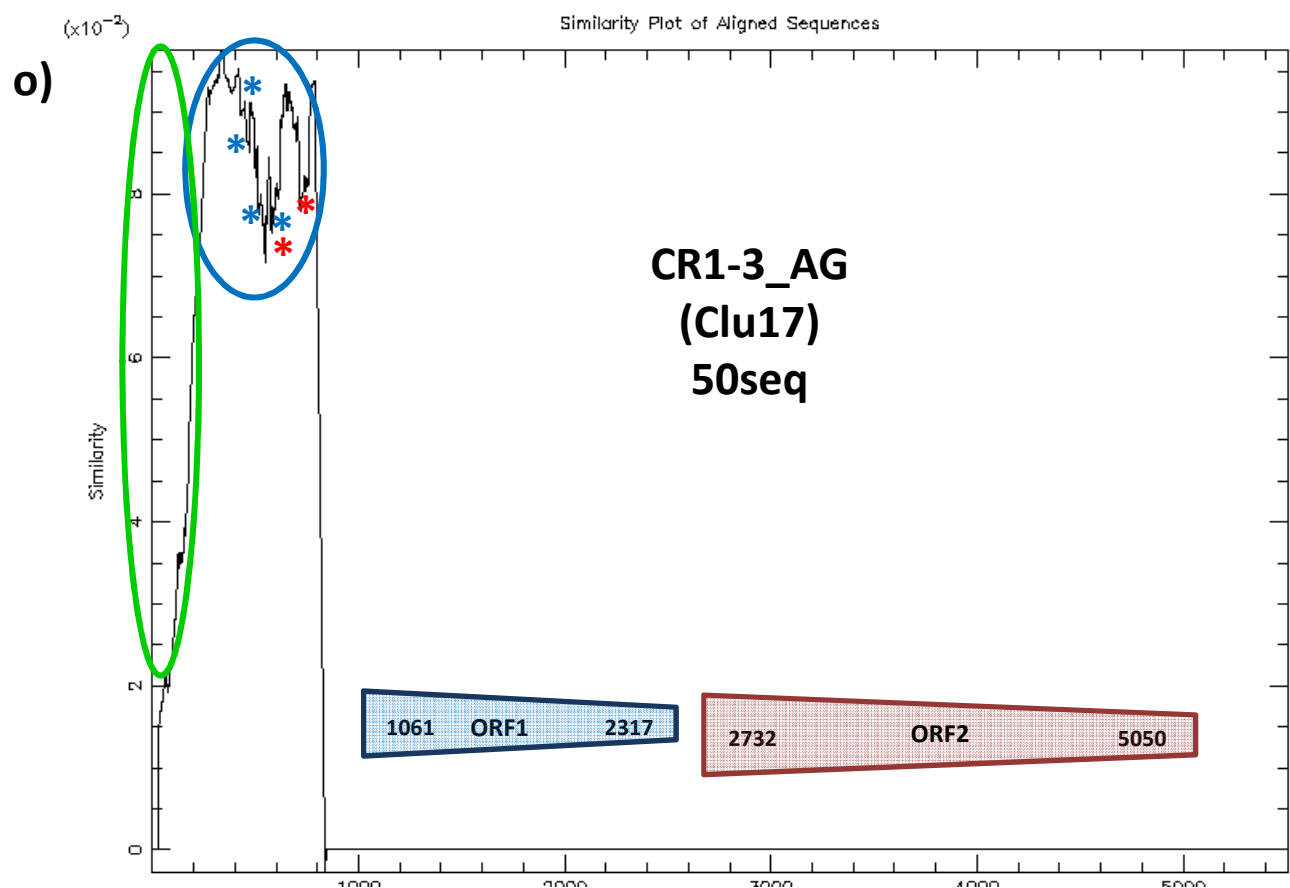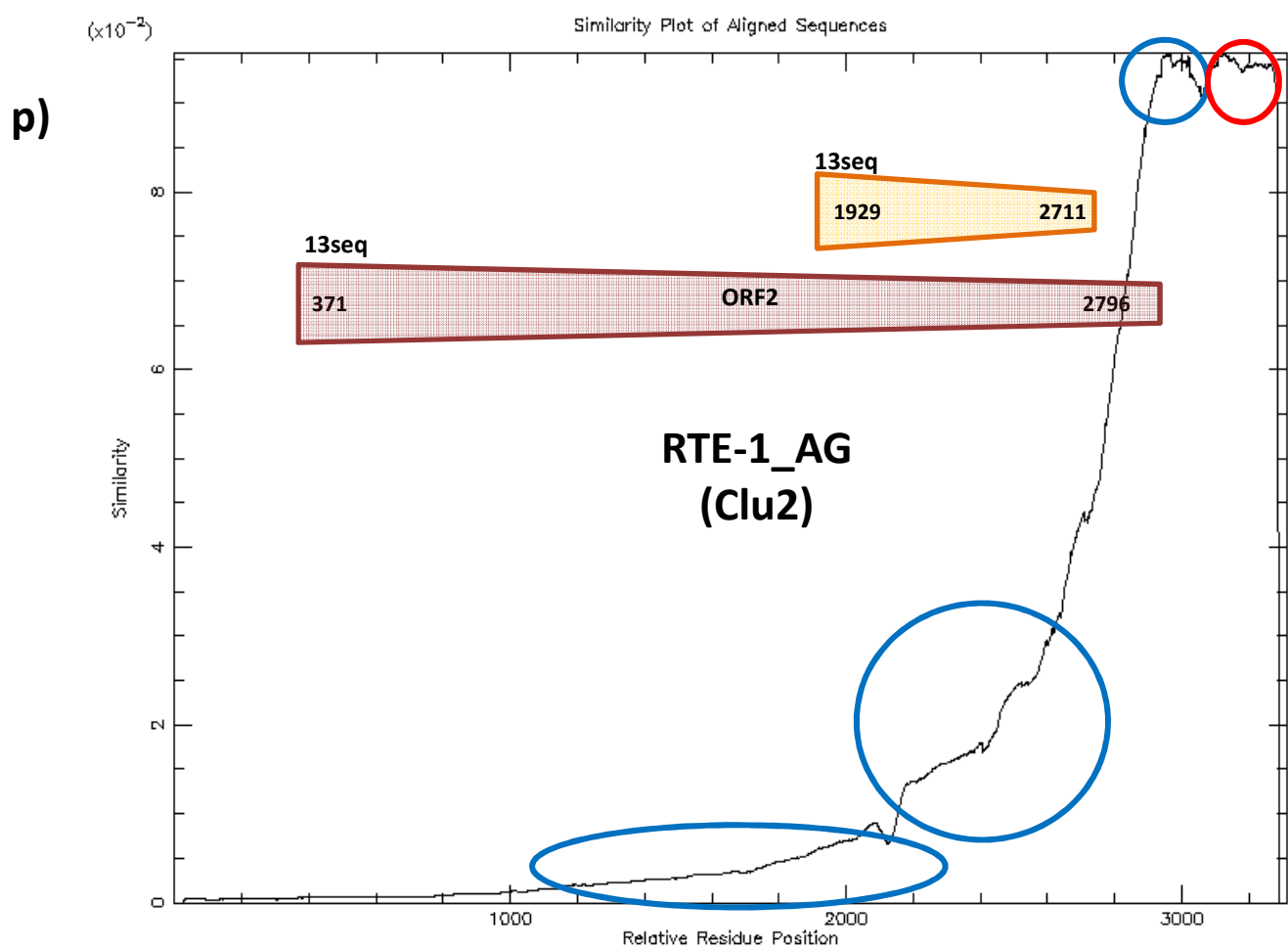

q)

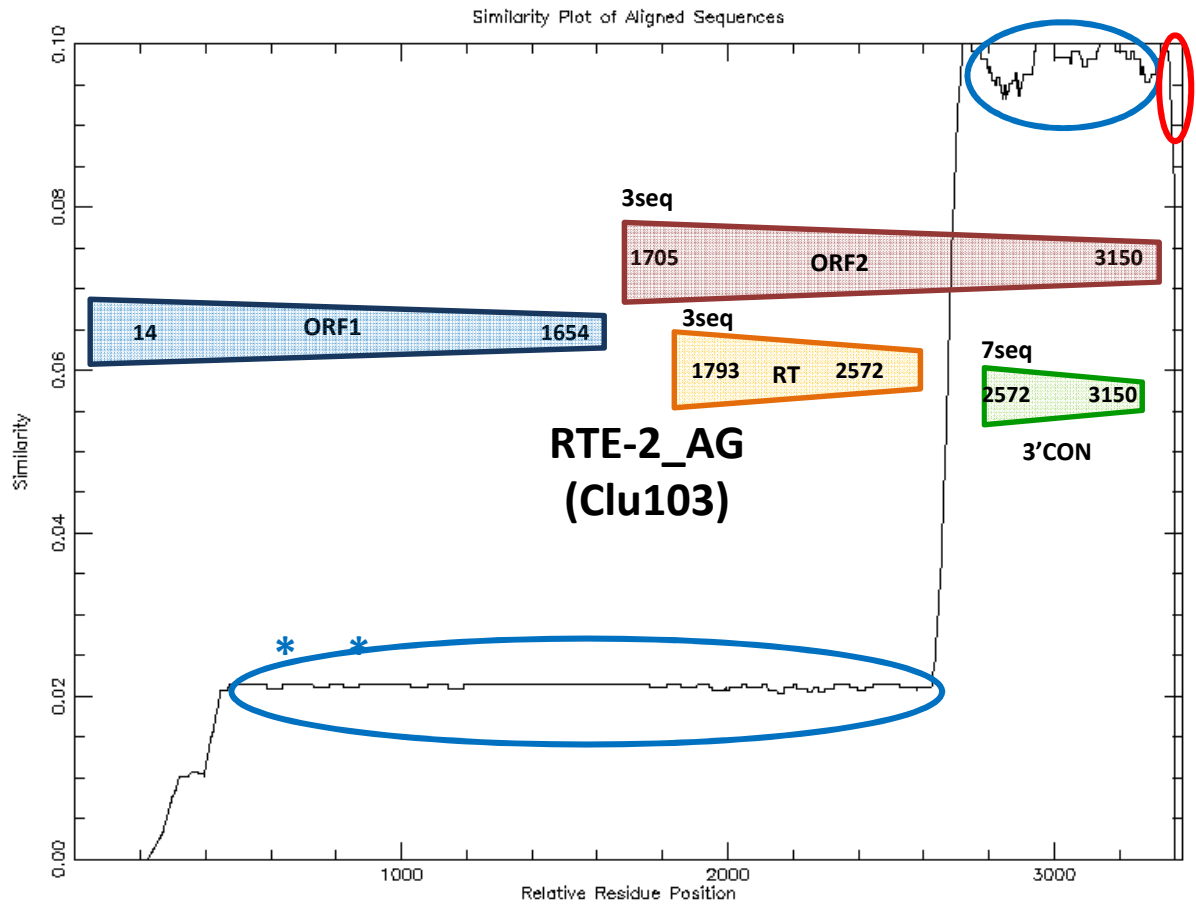

r)

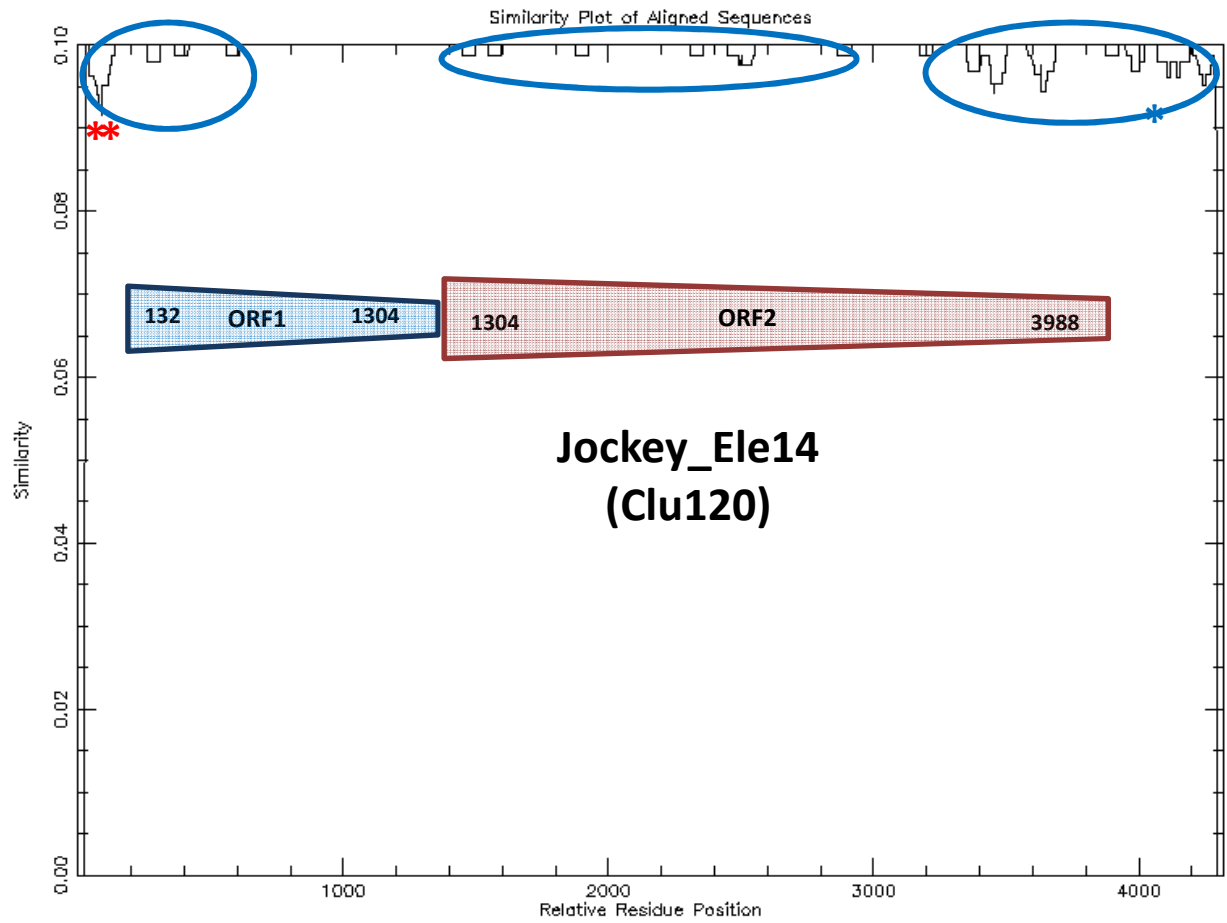

s)

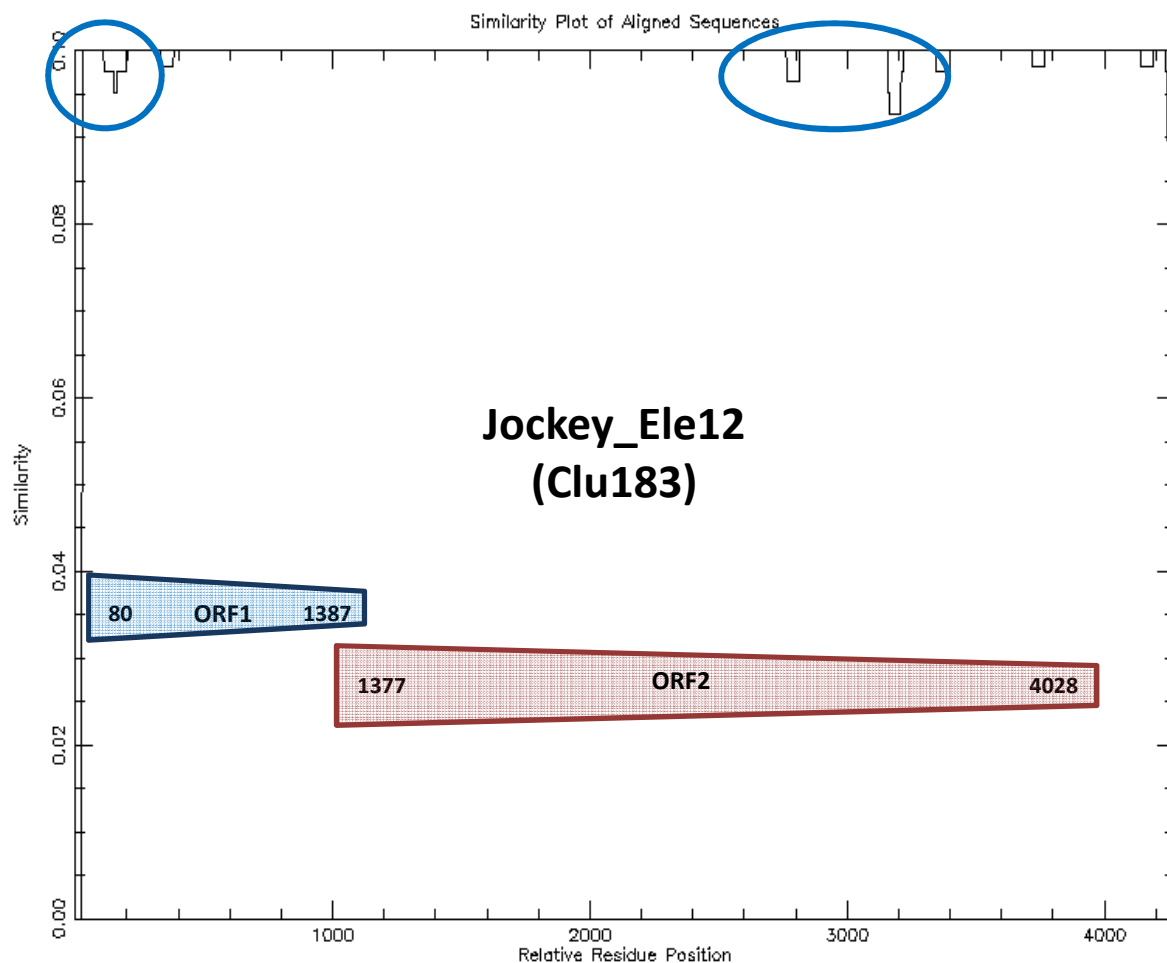

t)

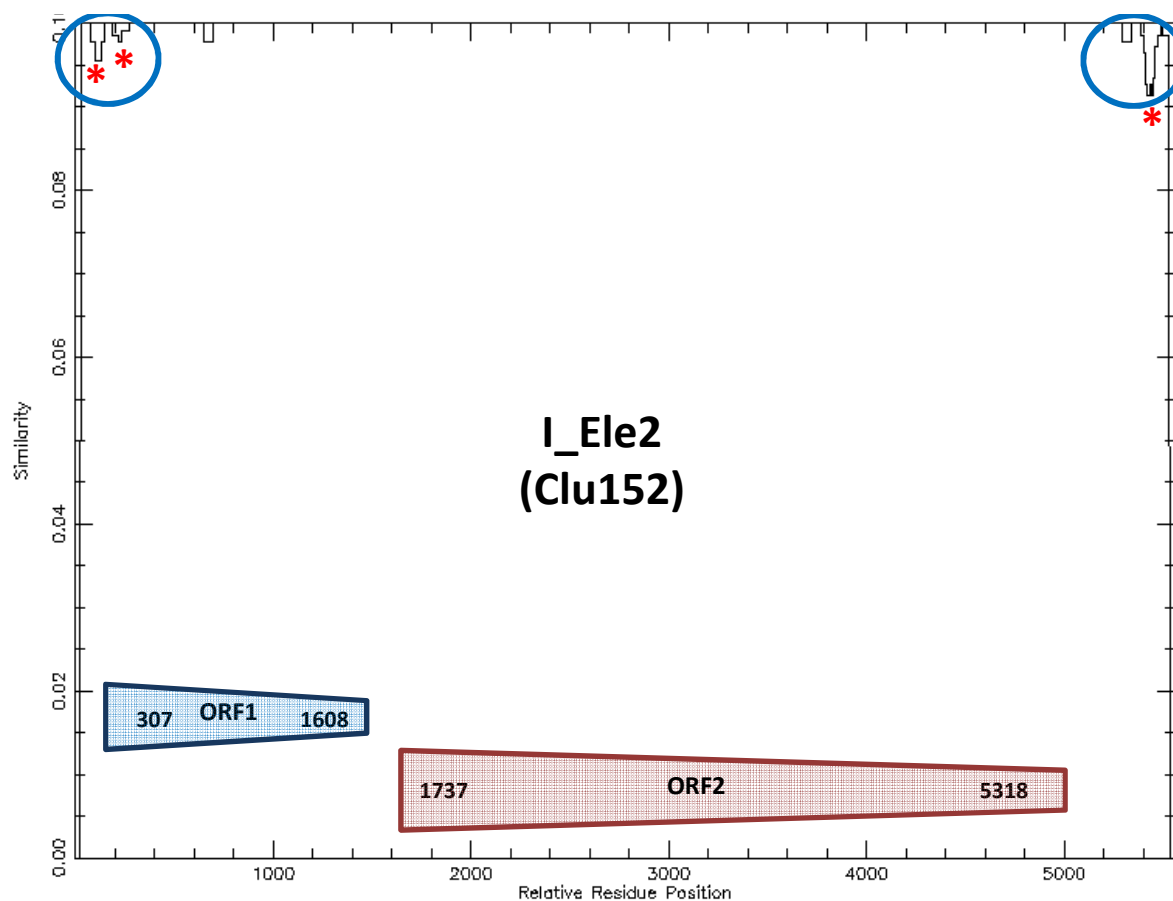

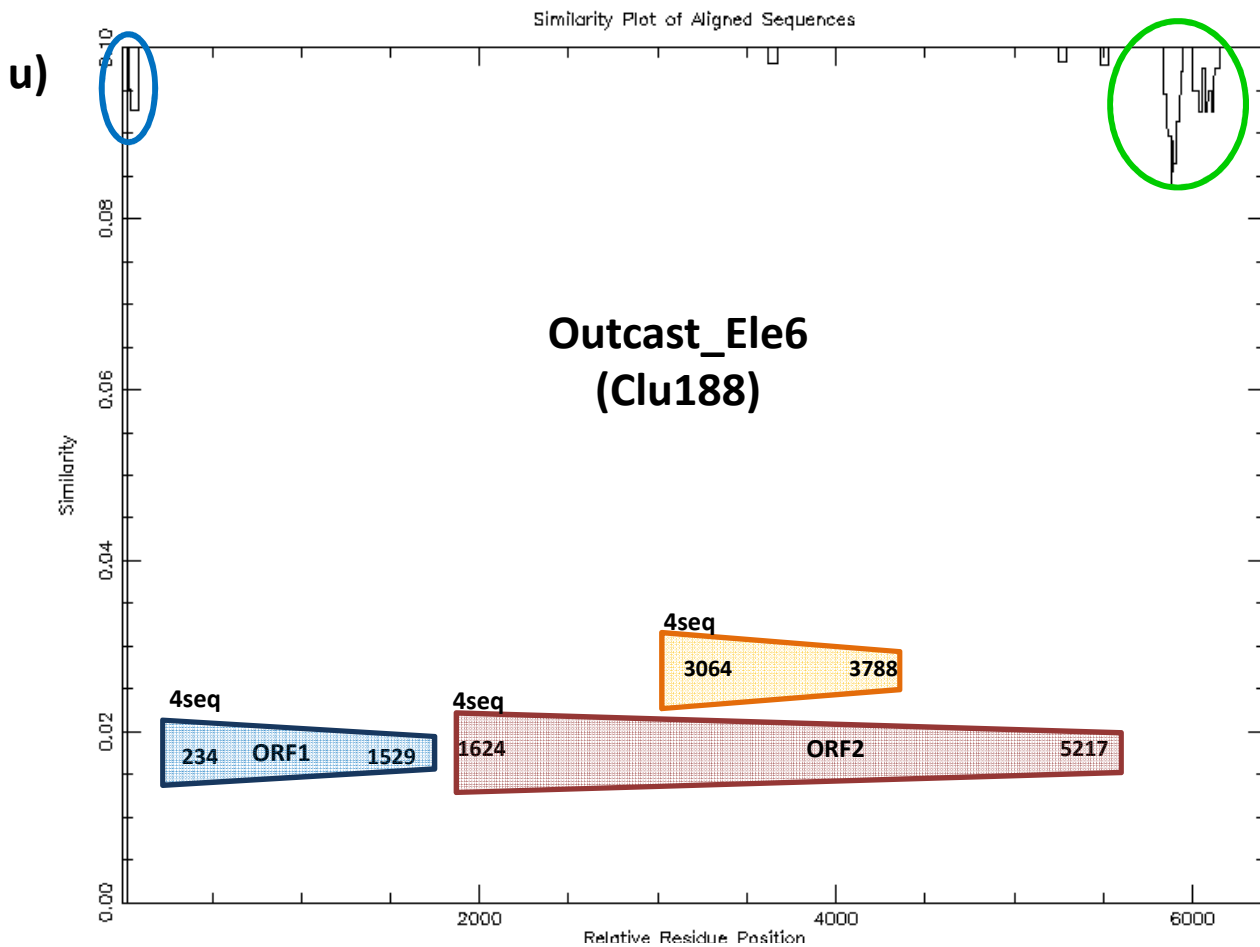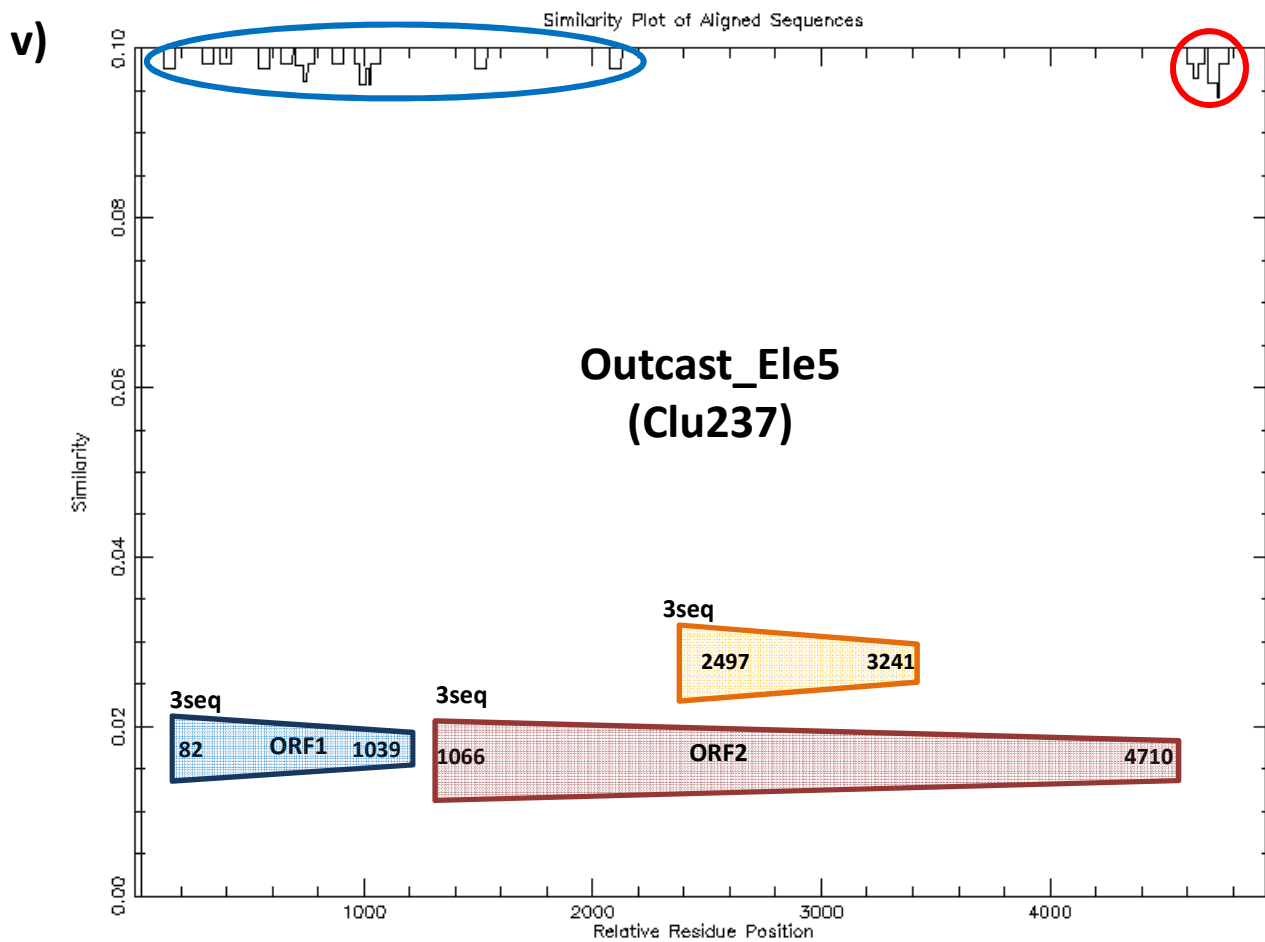

Supplement: Additional file 3 — Figure S2. Graphic representation of the deterioration profiles for the non-LTR families analyzed in this study. See legend for Additional file 1: Figure S1 for detailed information. (a-e) CR1 families composed of full-length and 5′ truncated sequenced; (f–m) CR1 families composed of 5′ truncated sequences; (n,o) CR1 families containing 3′ truncated sequences; (p,q) families of RTE elements composed of full-length and 5′ truncated sequences, (r,s) Jockey families composed of full-length sequences, (t) family of I full-length sequences, (u,v) families of Outcast elements composed of full-length sequences. Colored arrows indicate the relative positions of ORF1 (blue), ORF2 (red), RT domain (yellow), and 3′ conserved region (green). The relative positions according to multiple alignments of the sequences to canonical element are indicated in each region. The number of sequences represented in each region is indicated above the arrows. (PDF 350 kb) [file 1471-2164-13-272-S3.pdf]

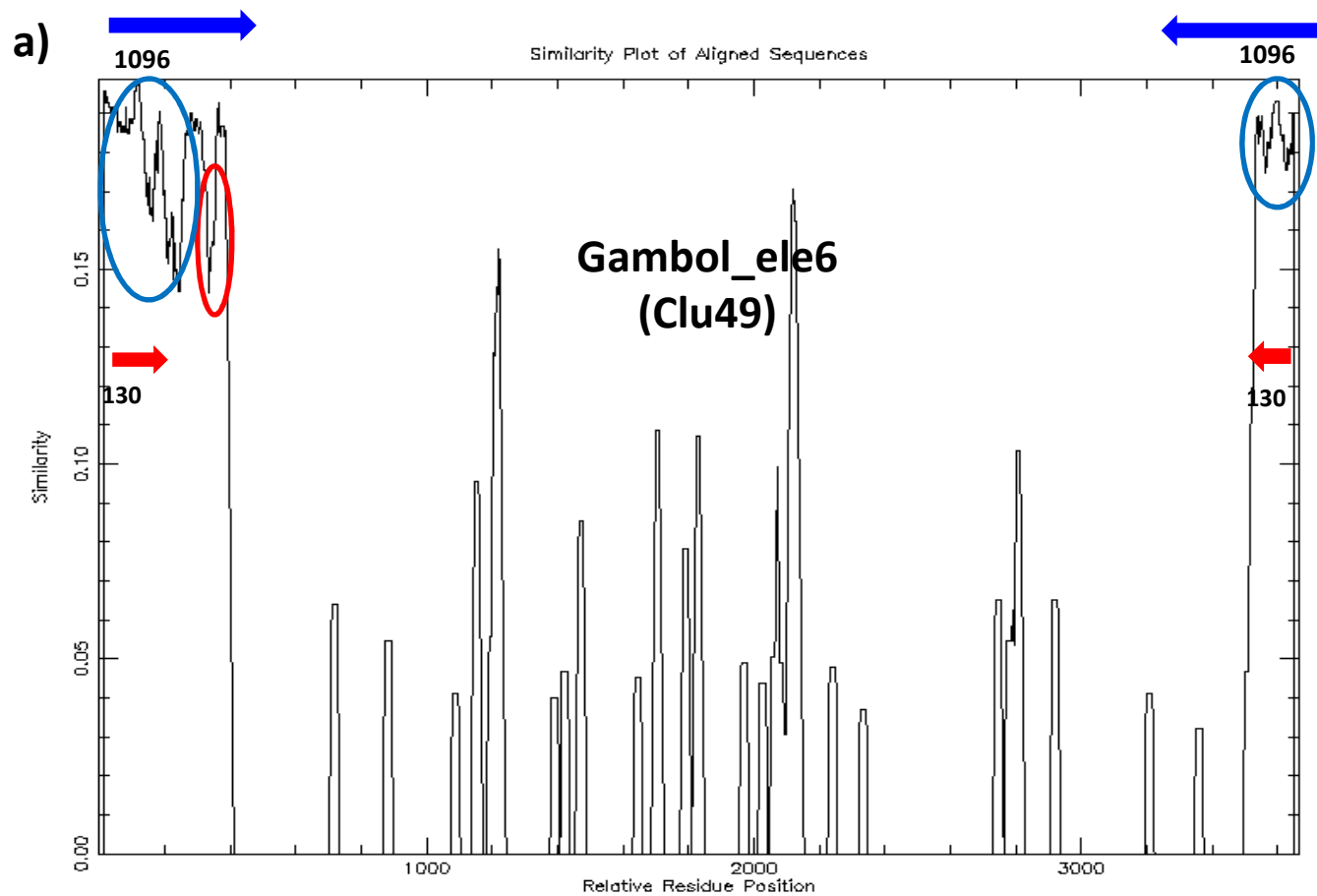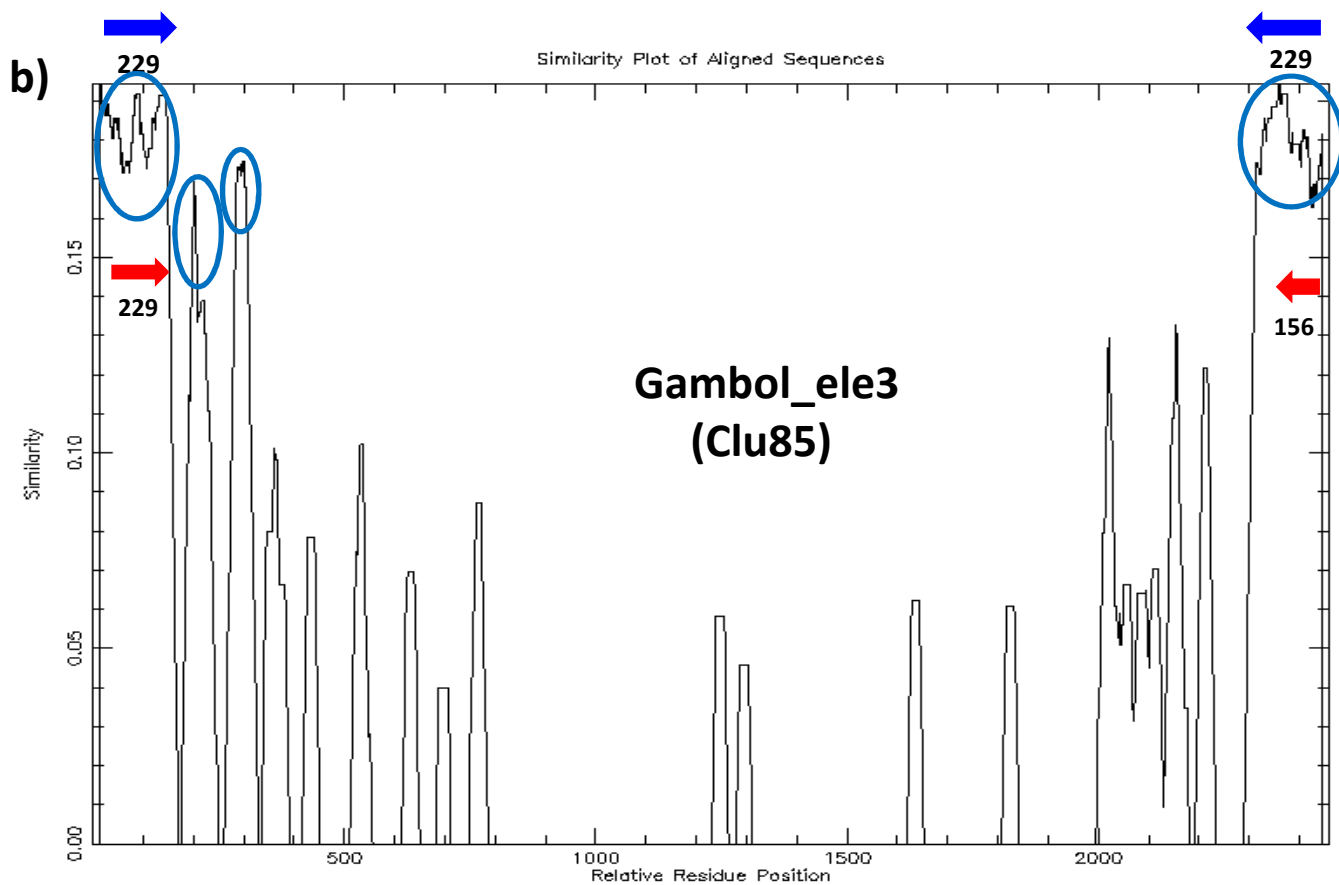

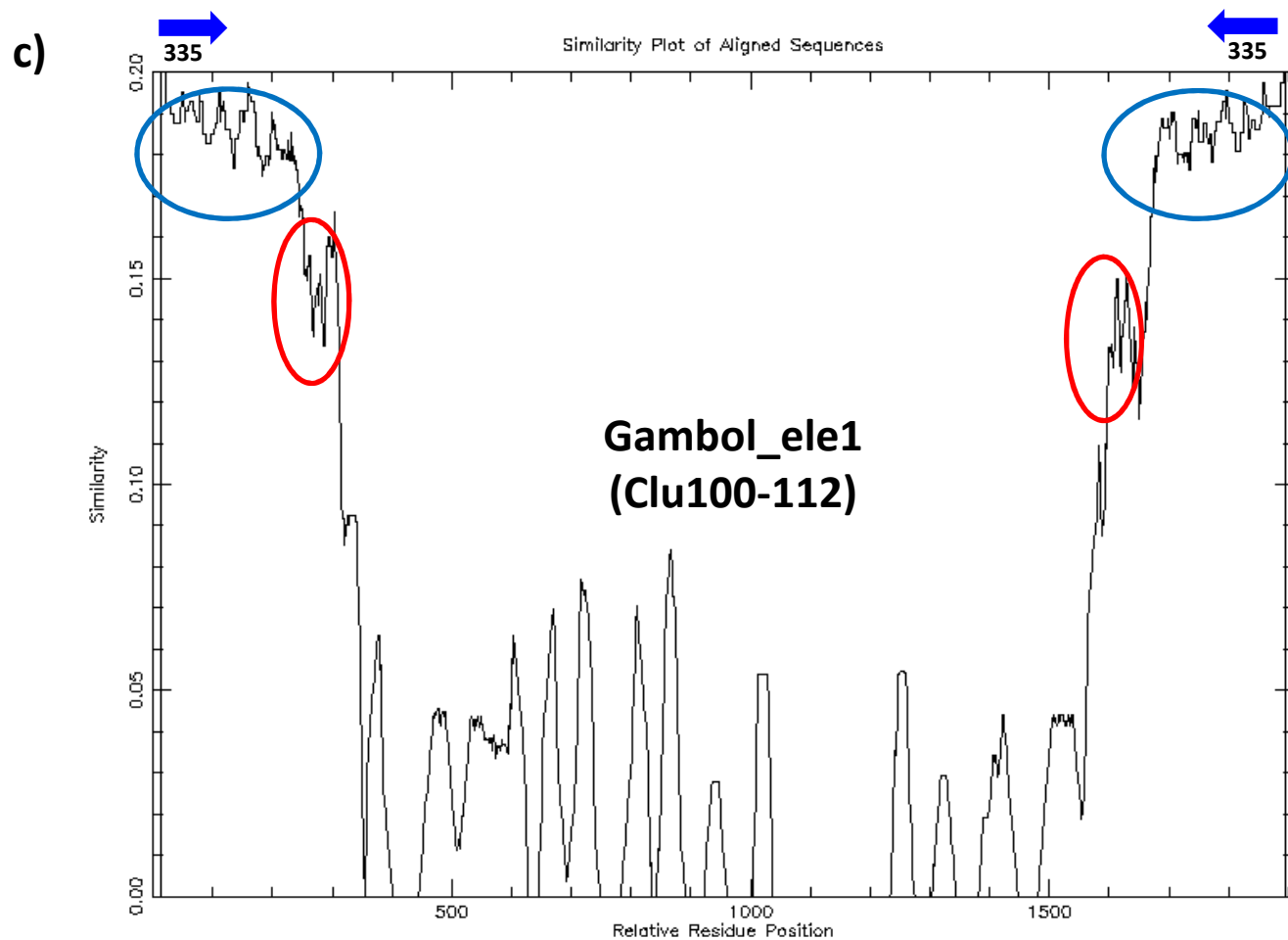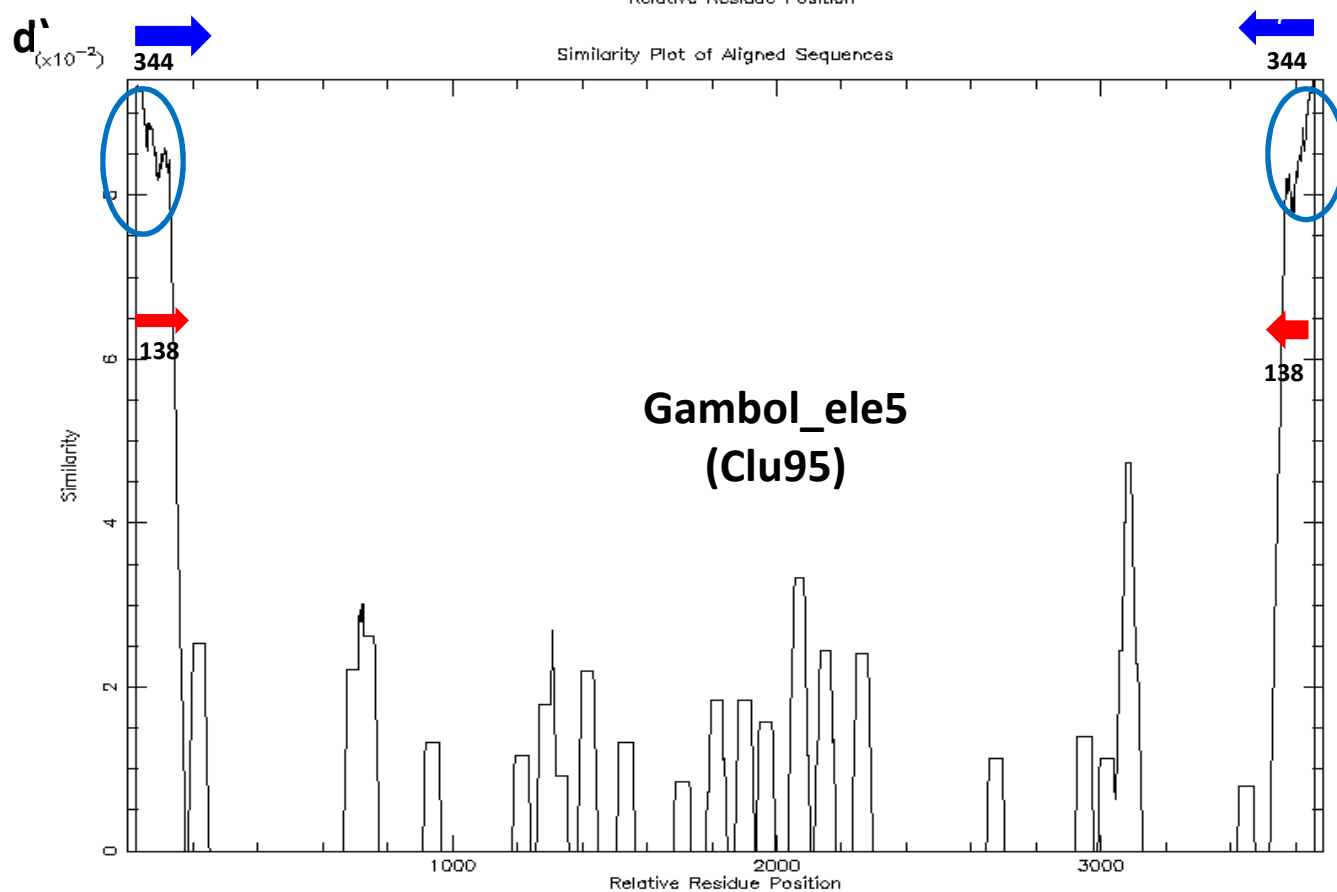

e)

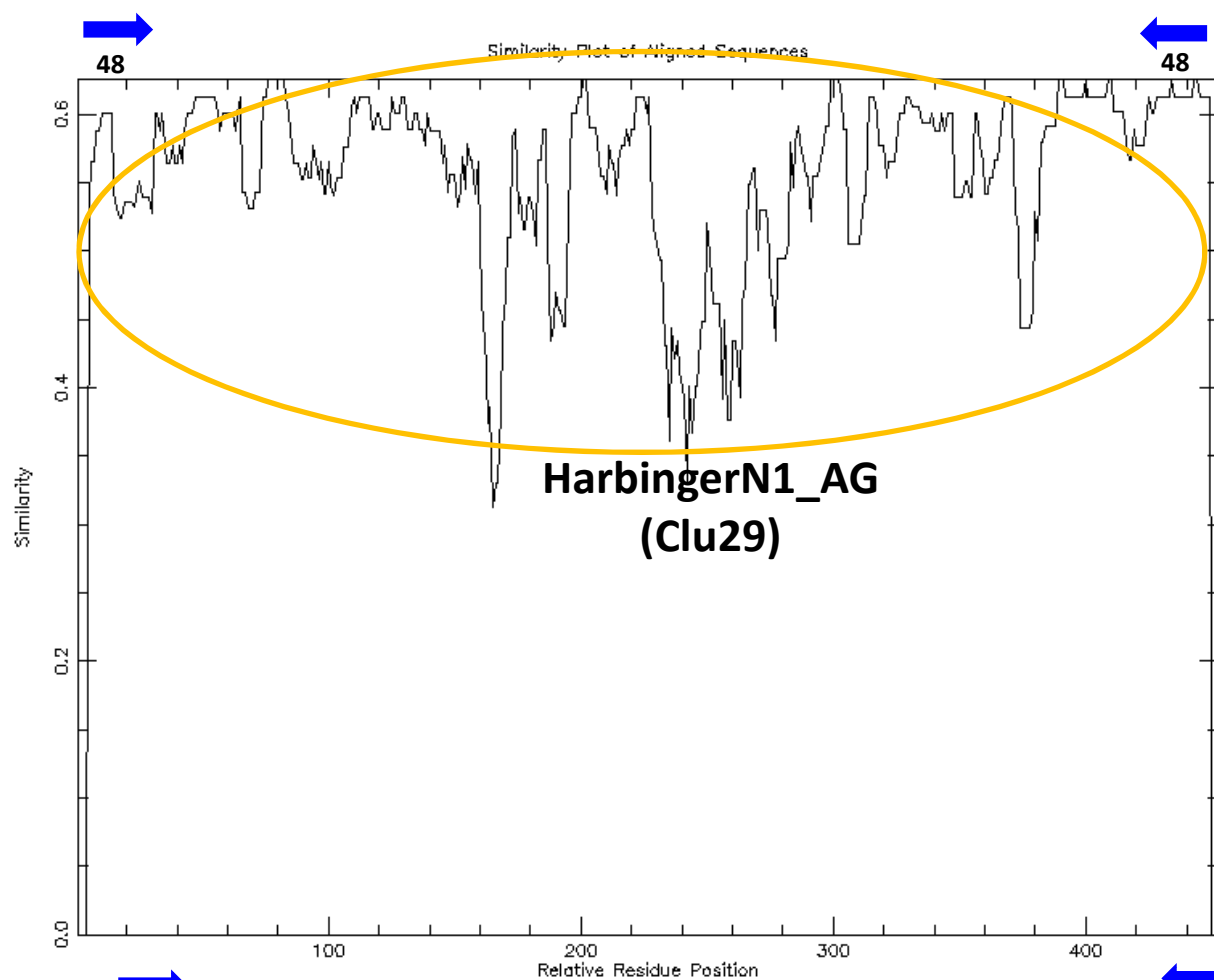

f)

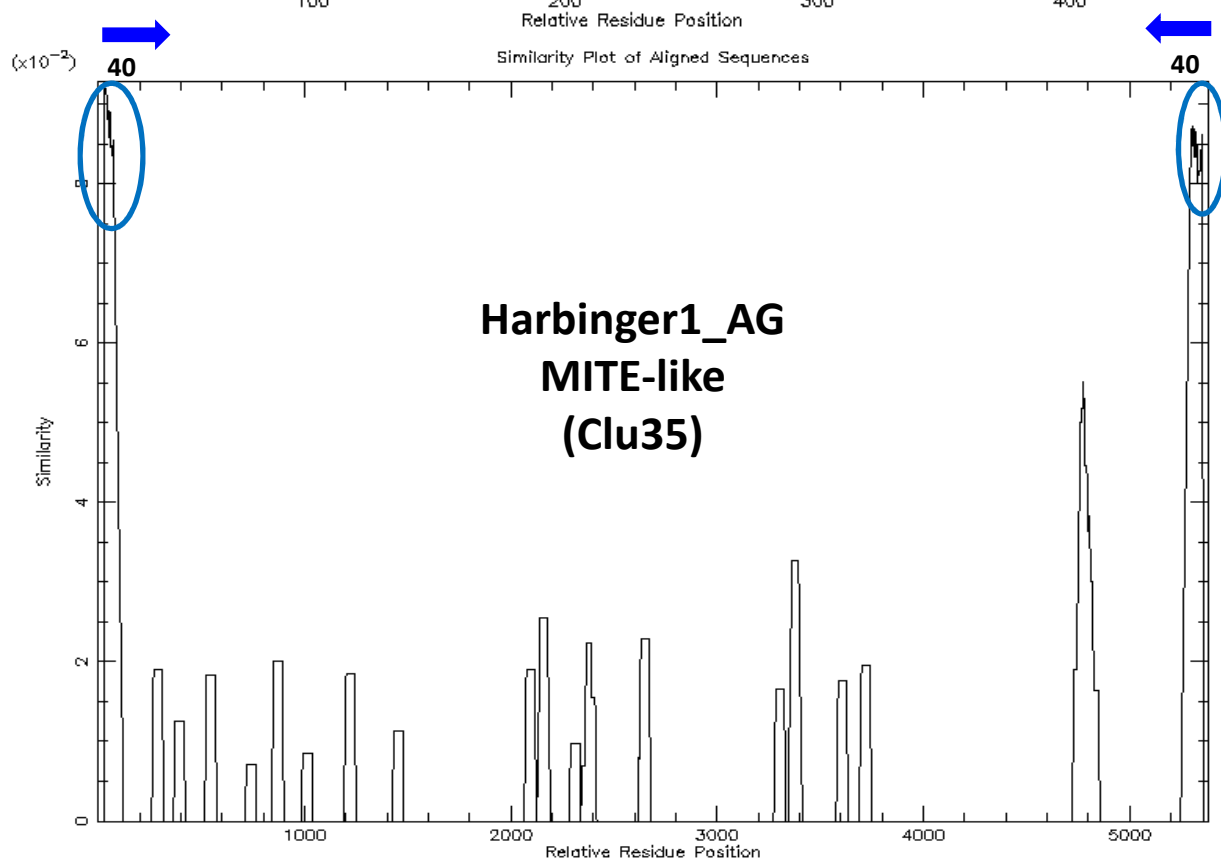

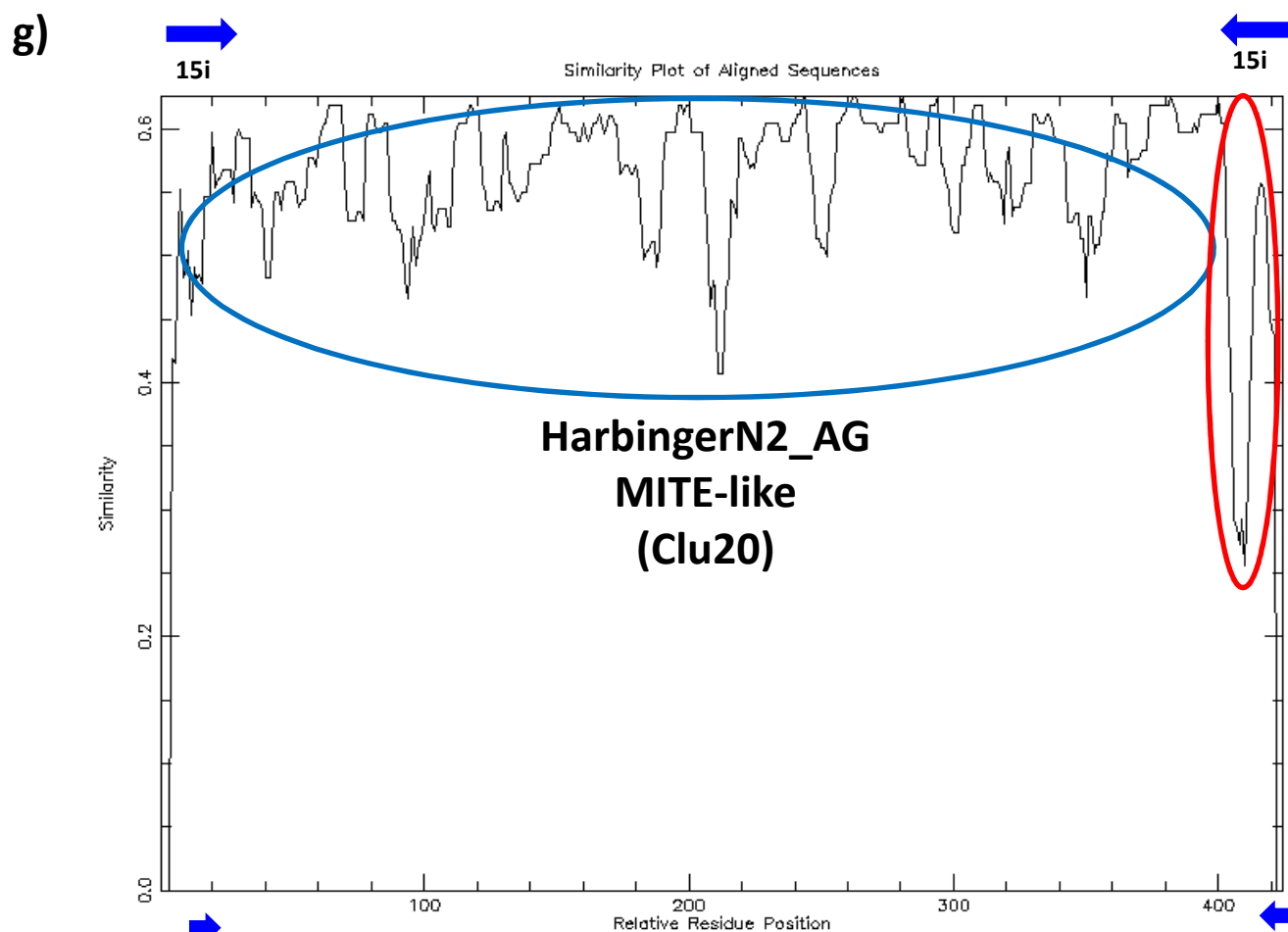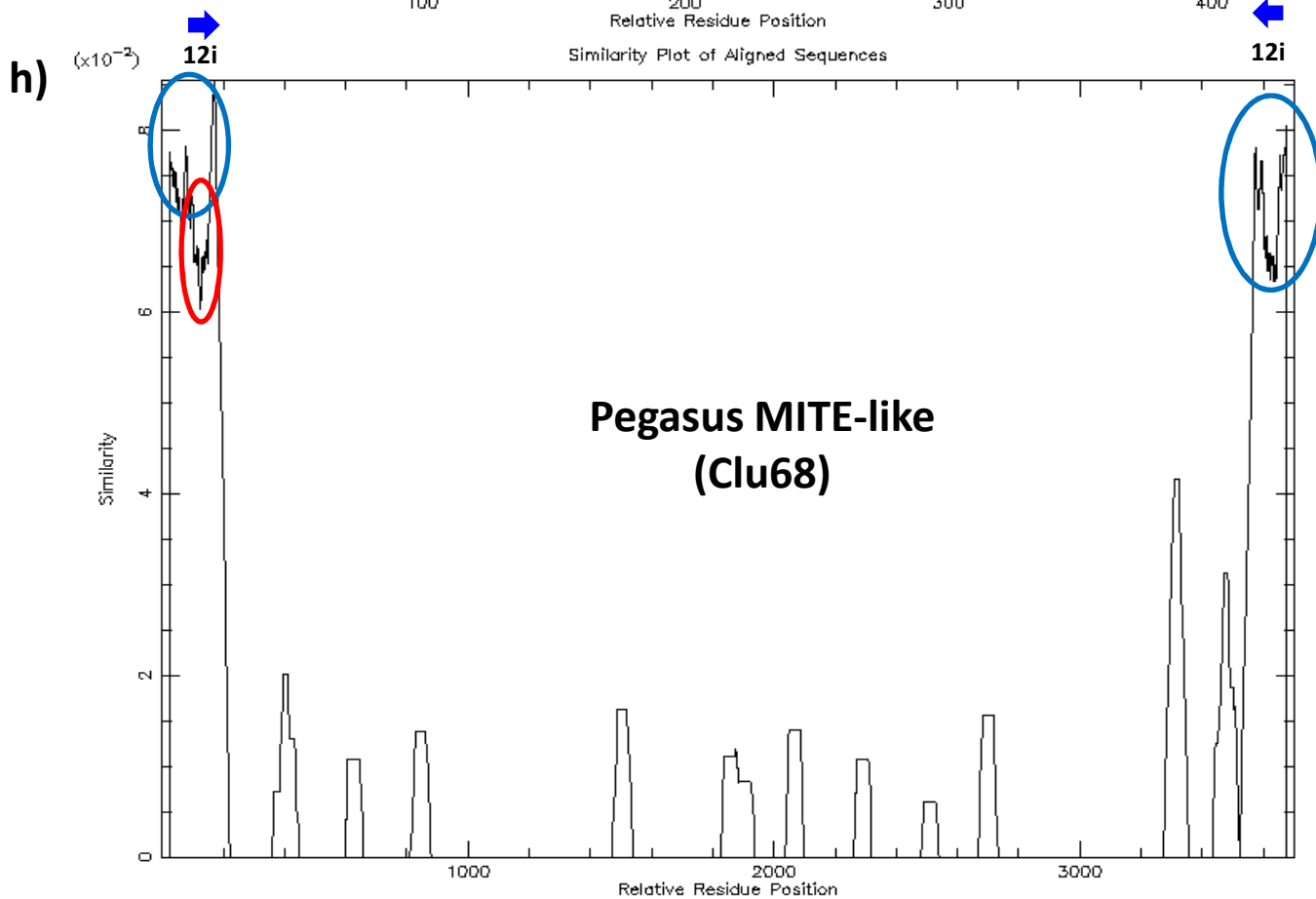

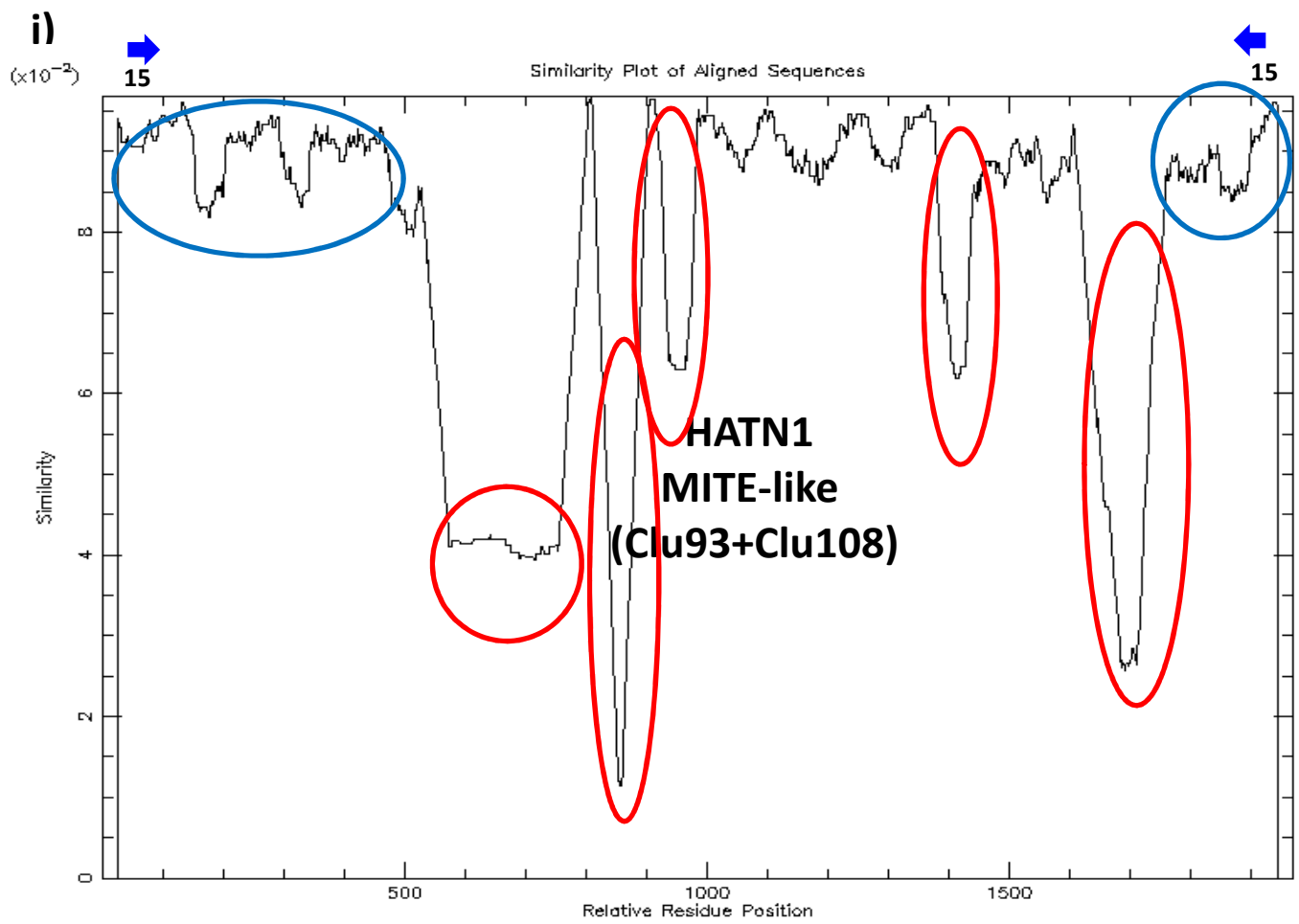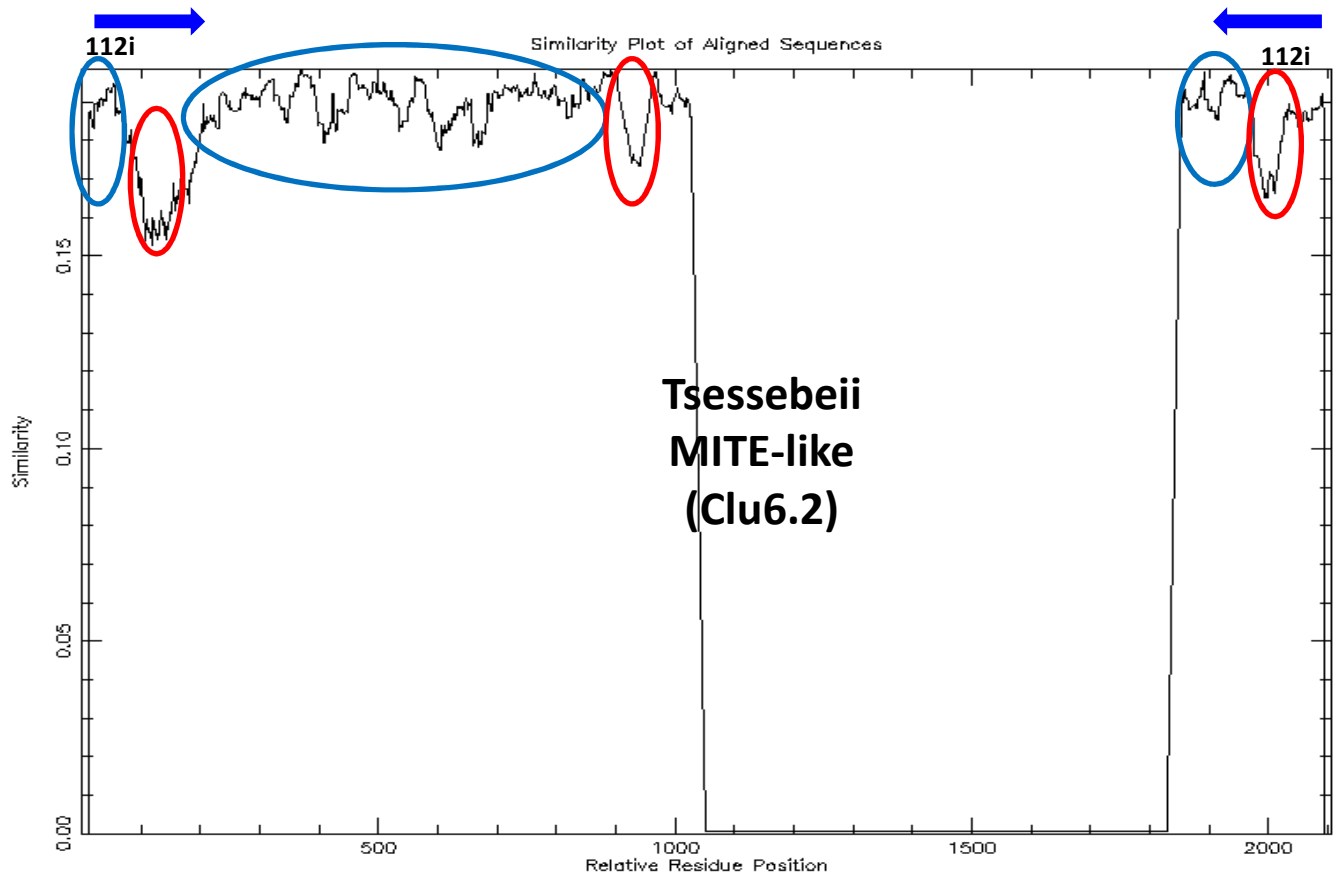

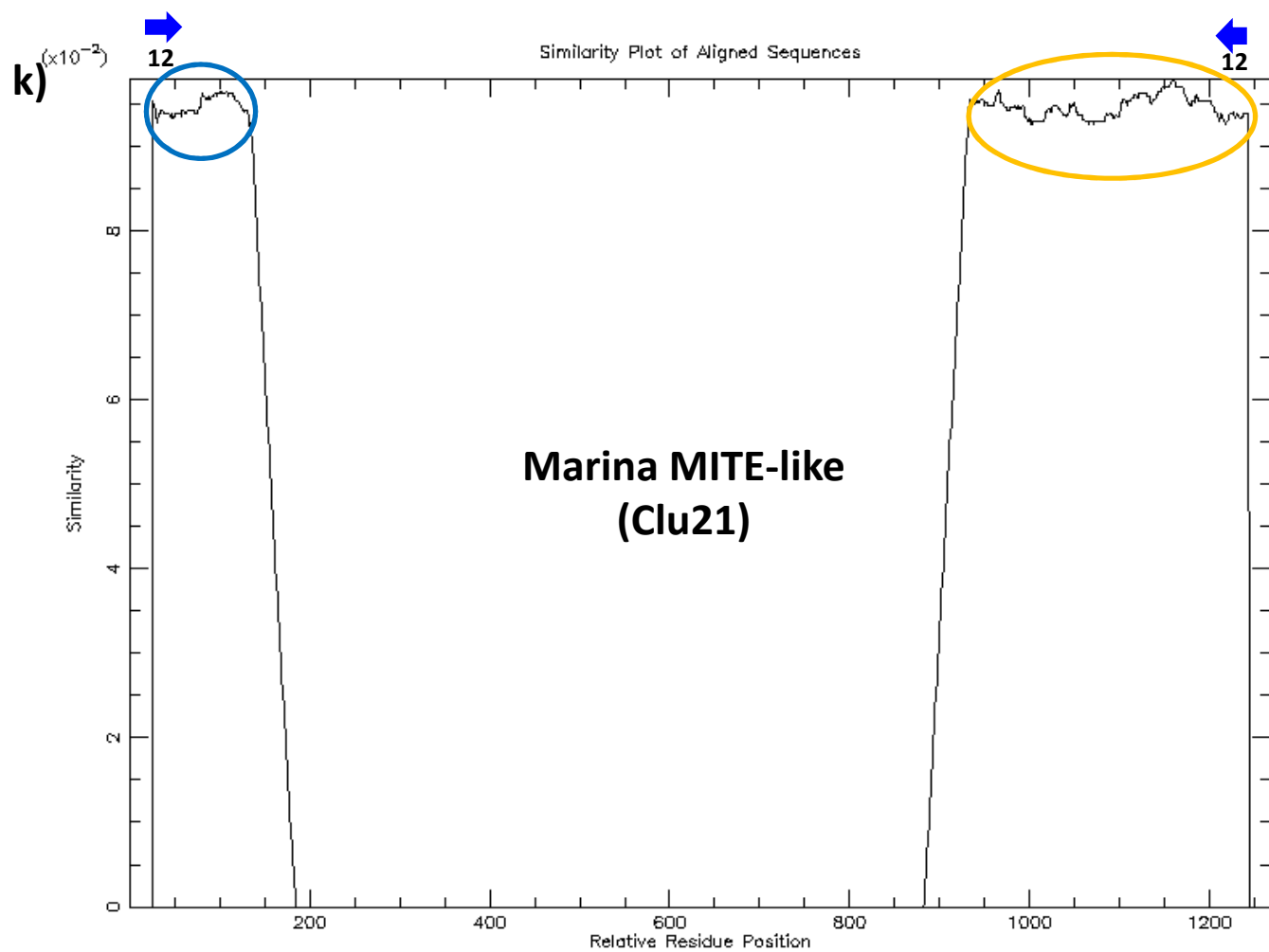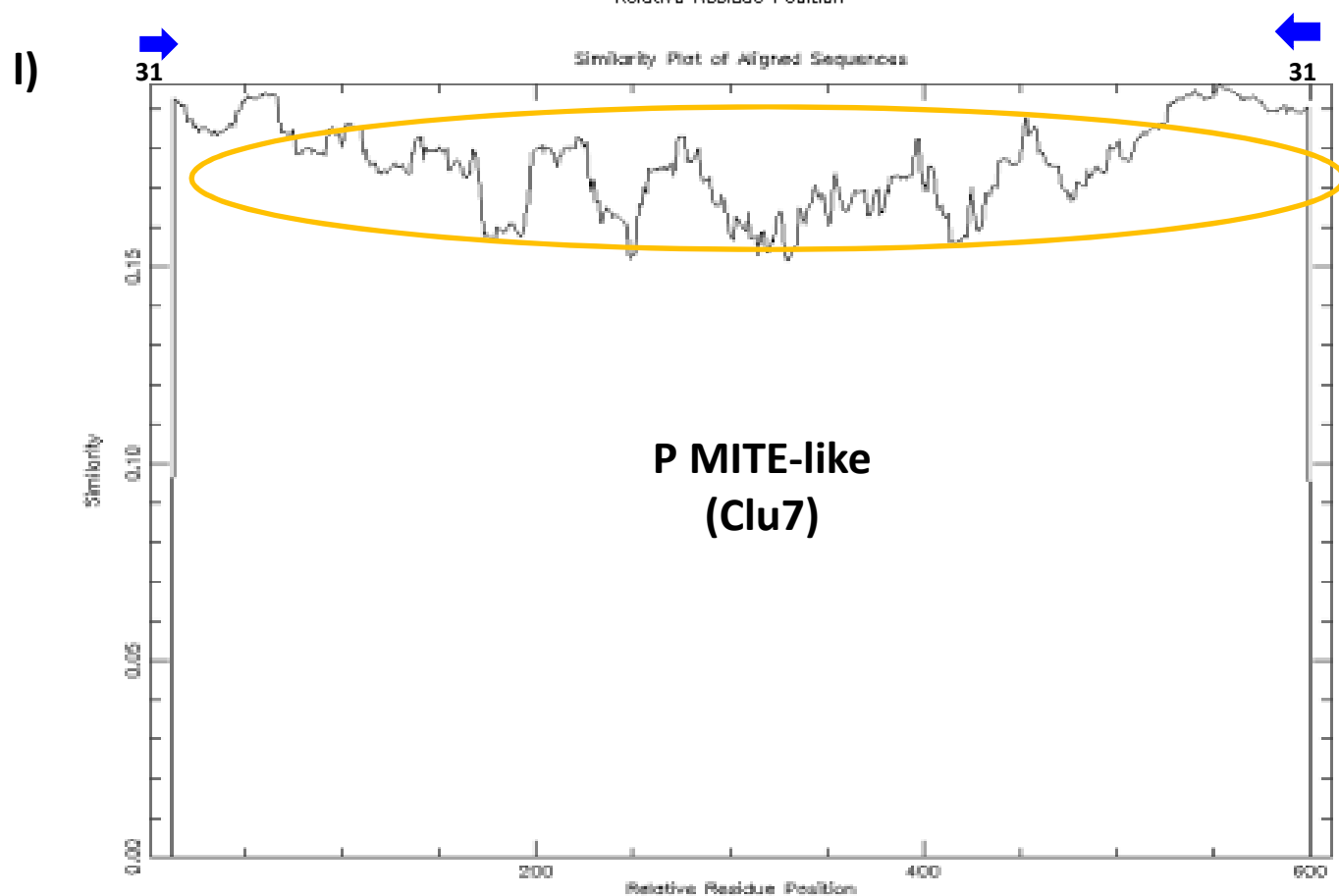

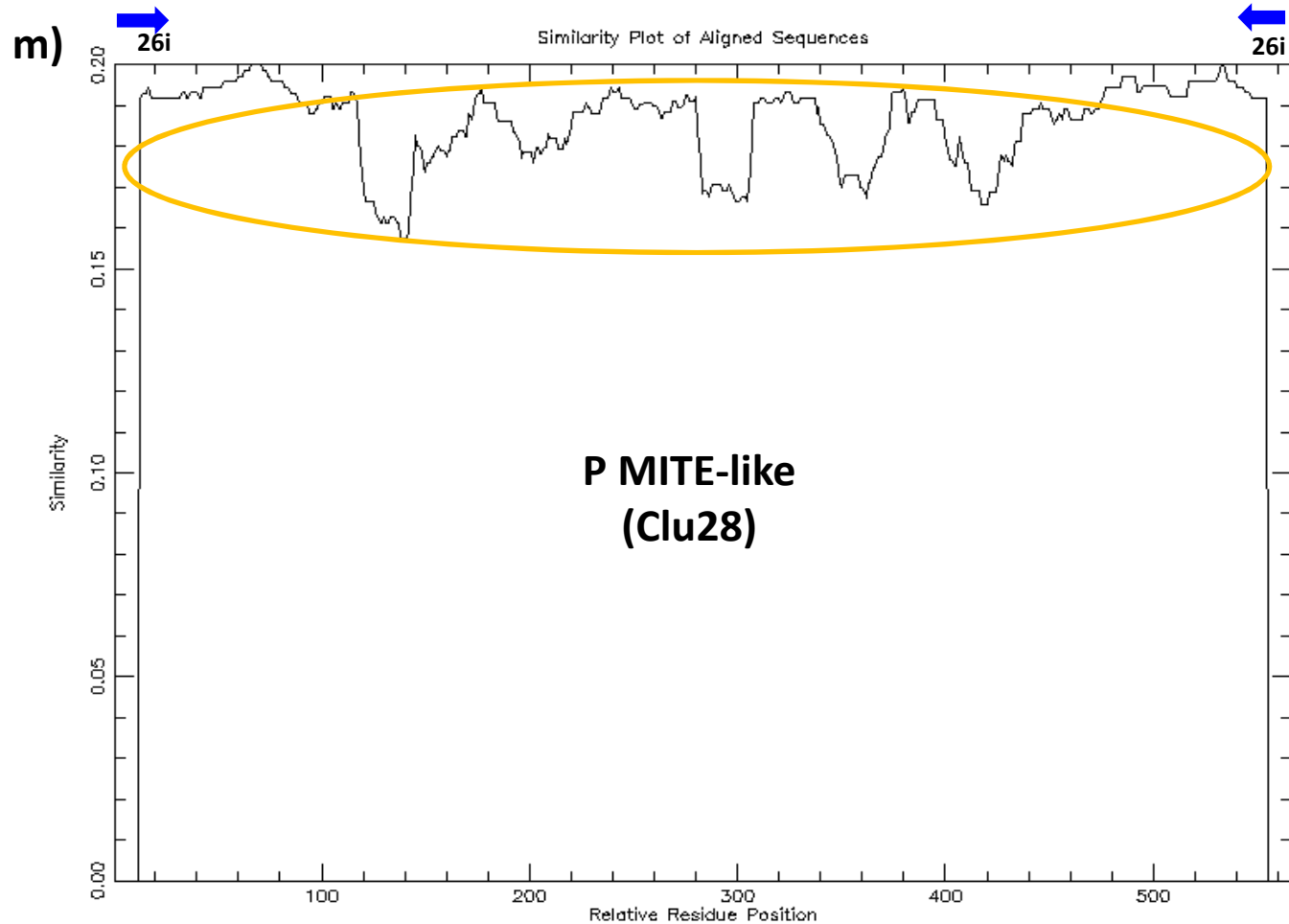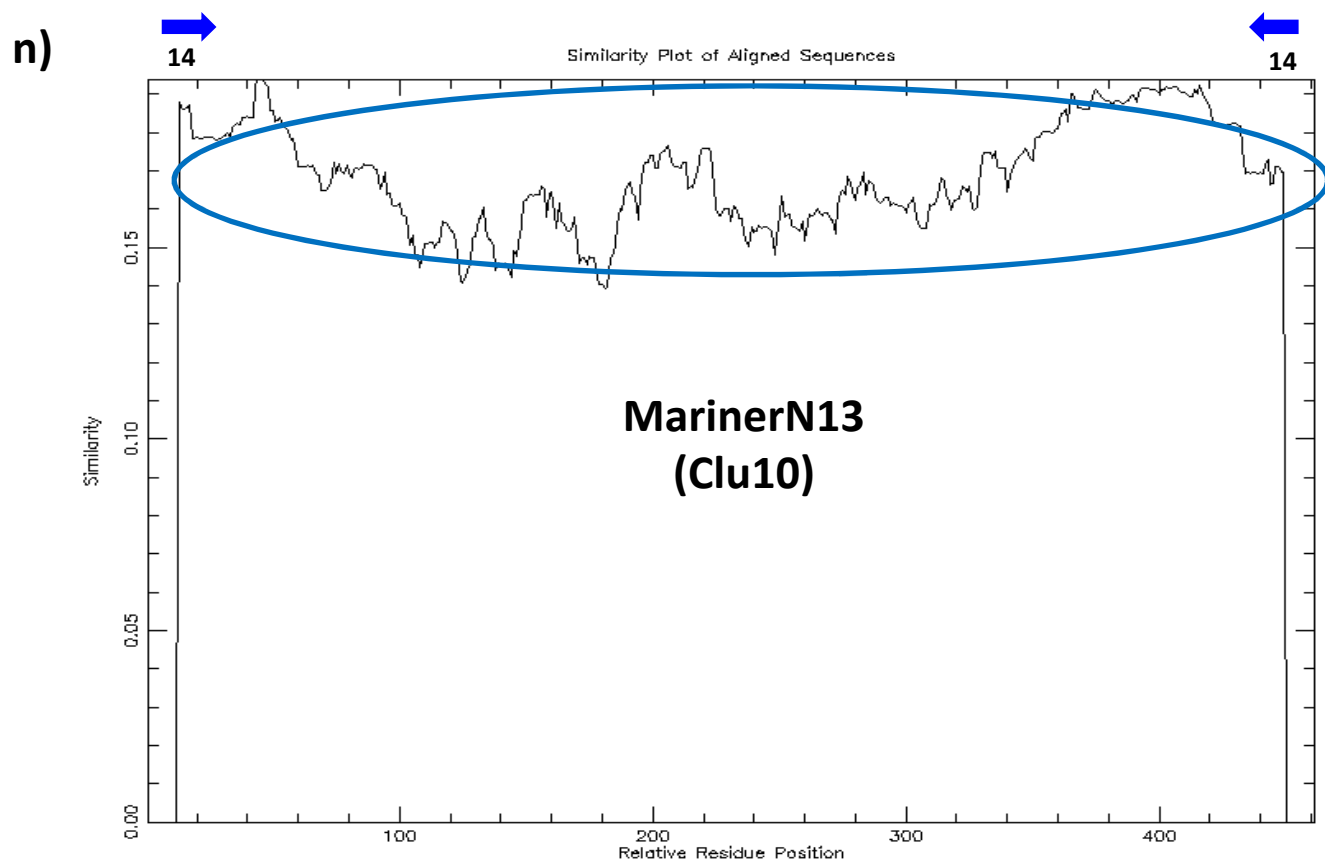

o)

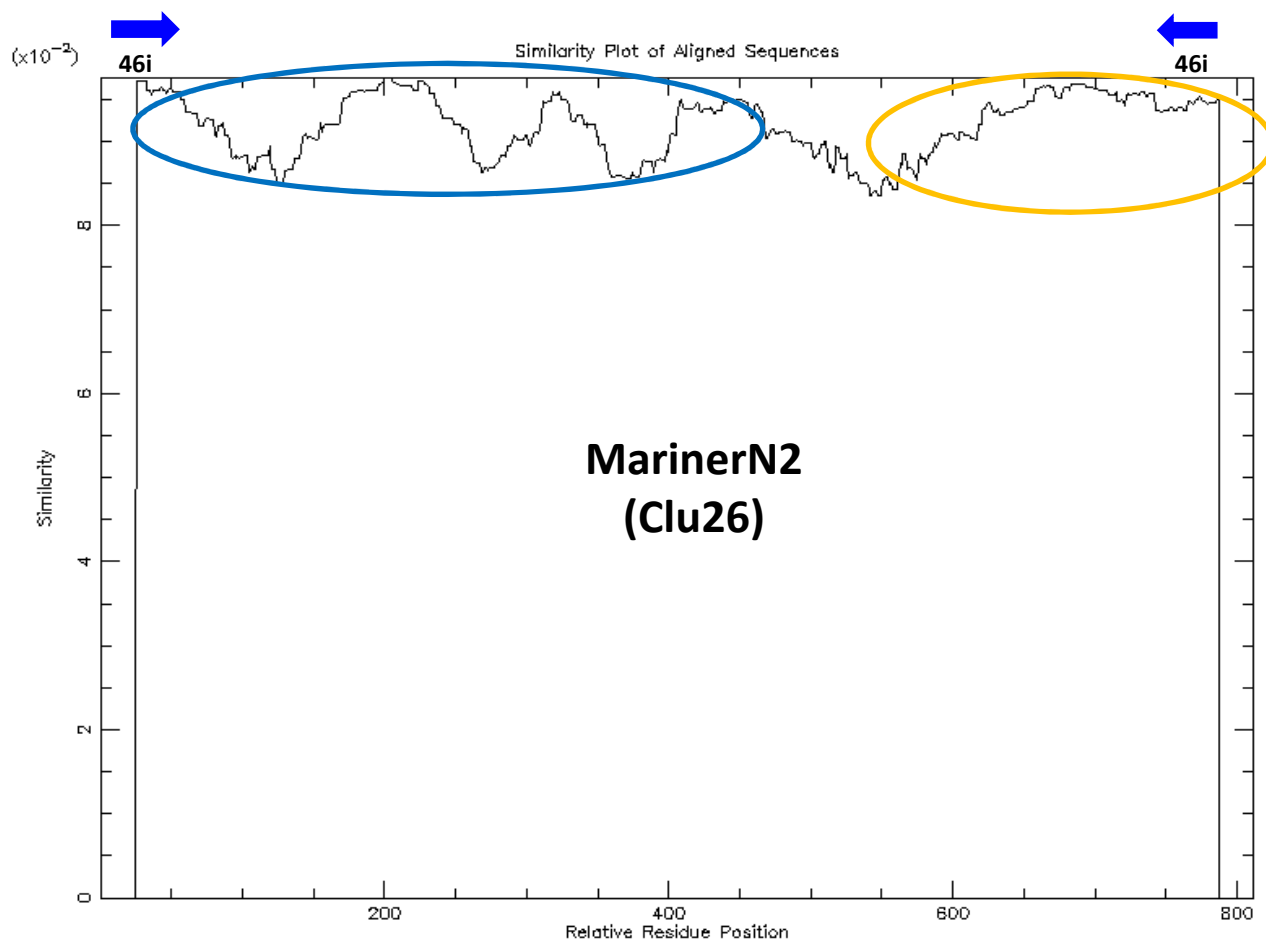

p)

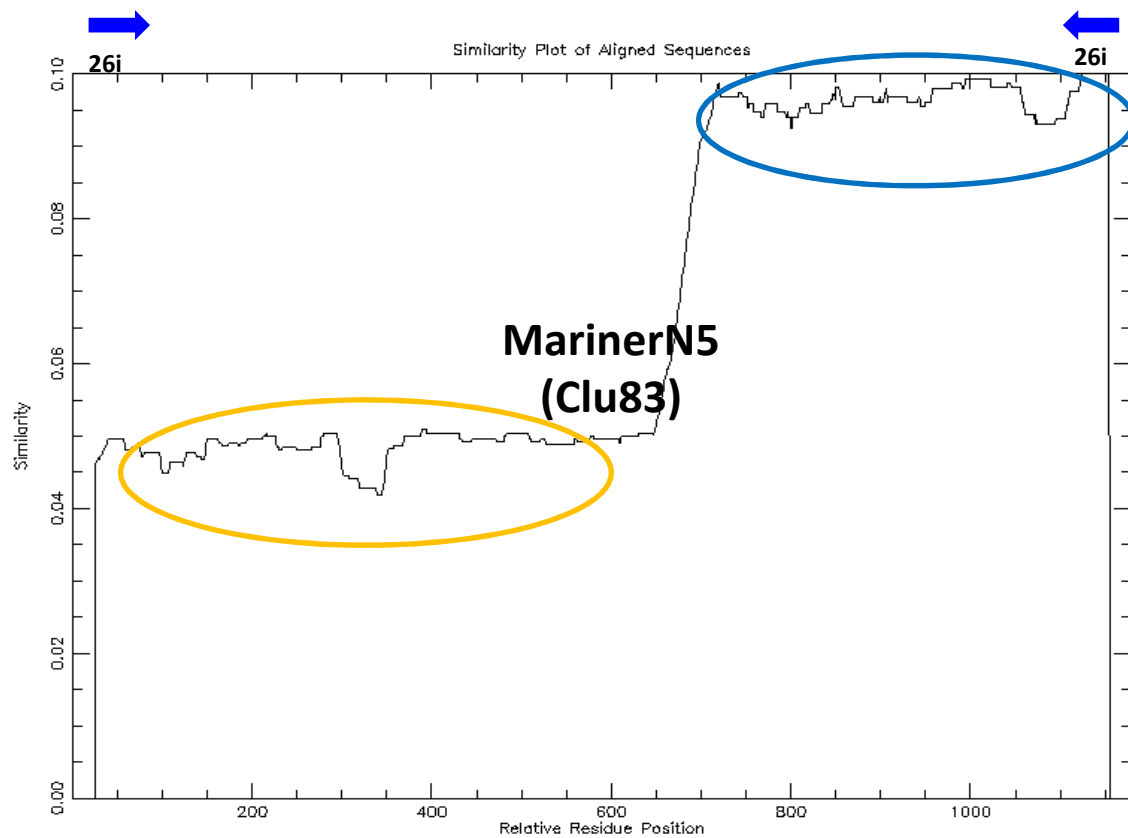

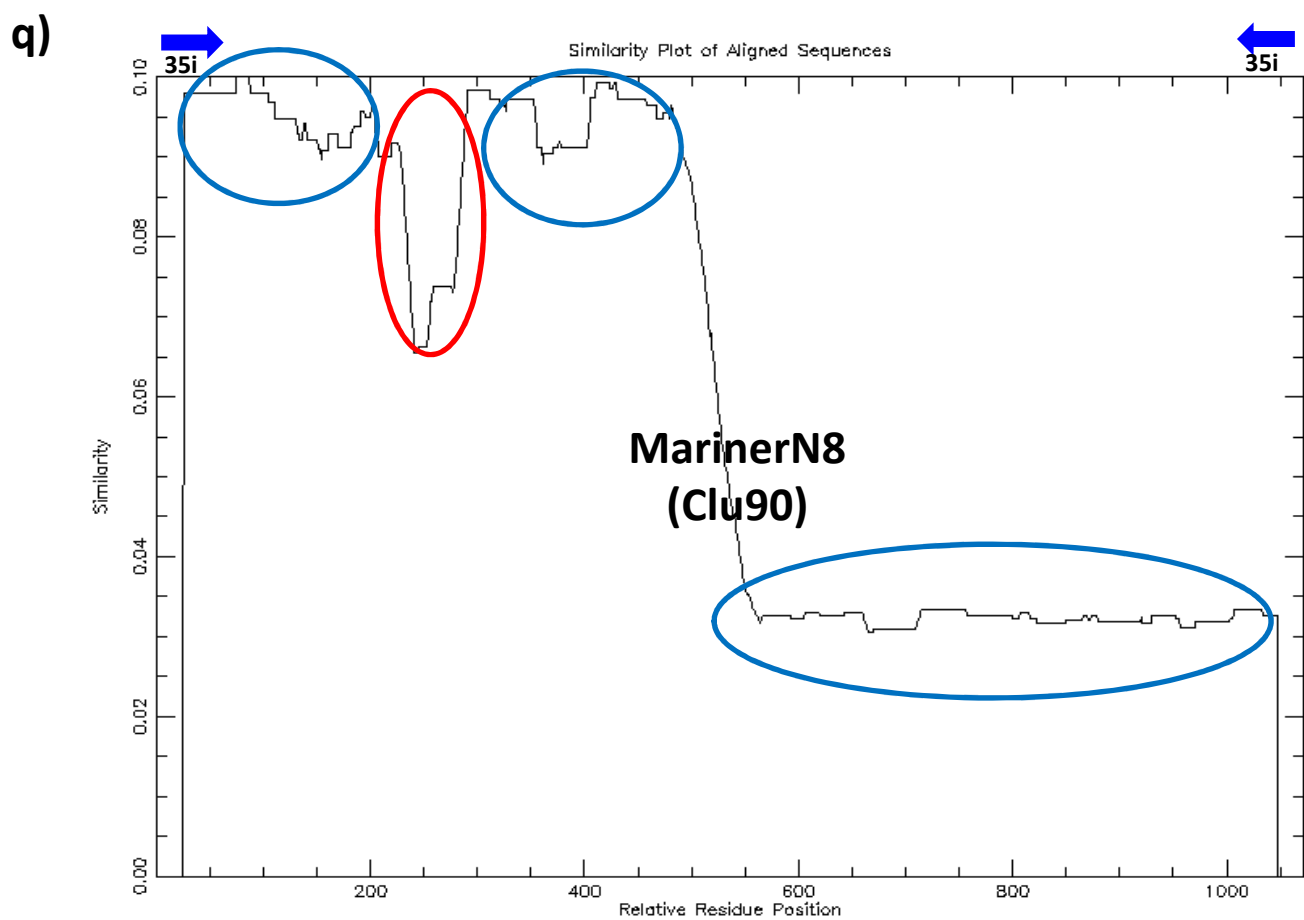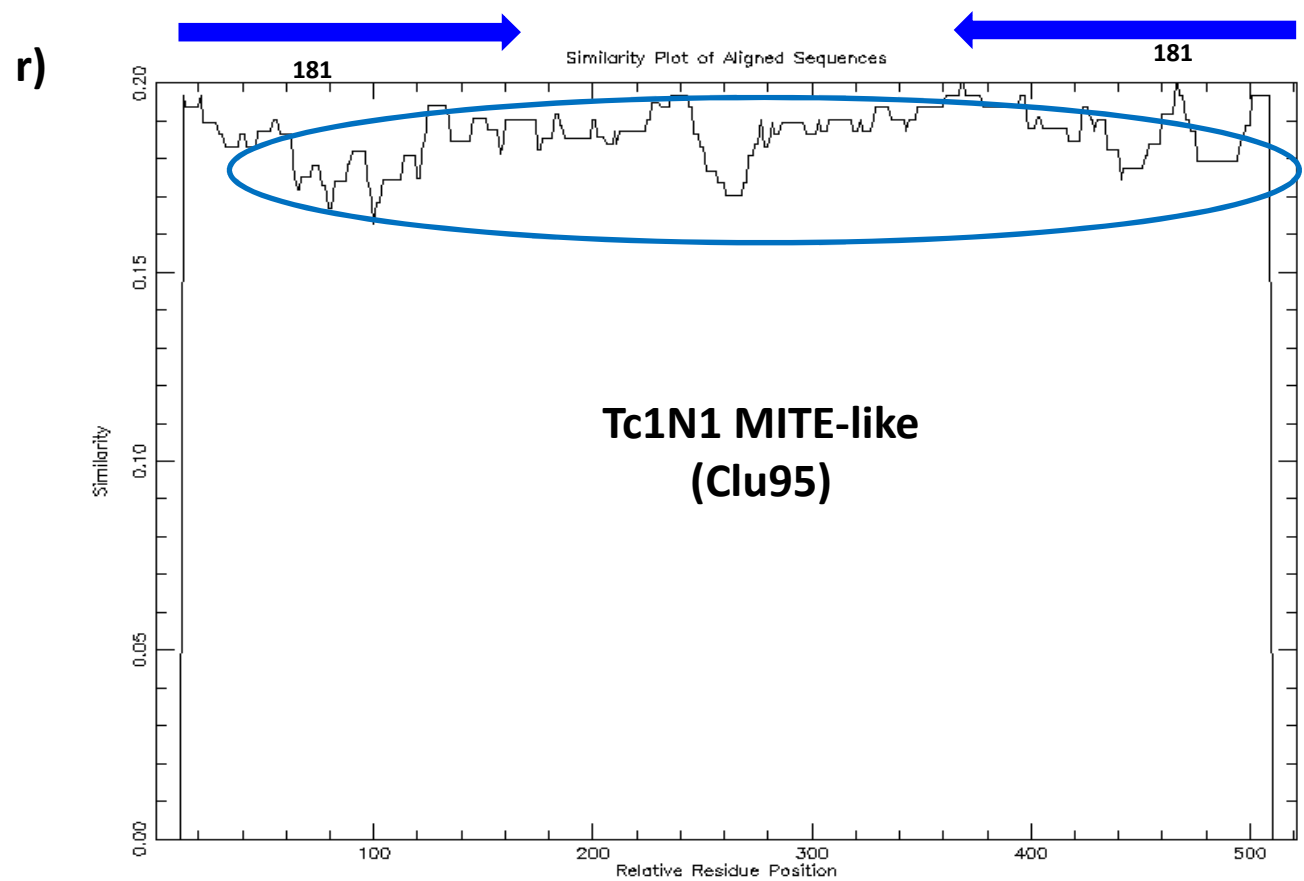

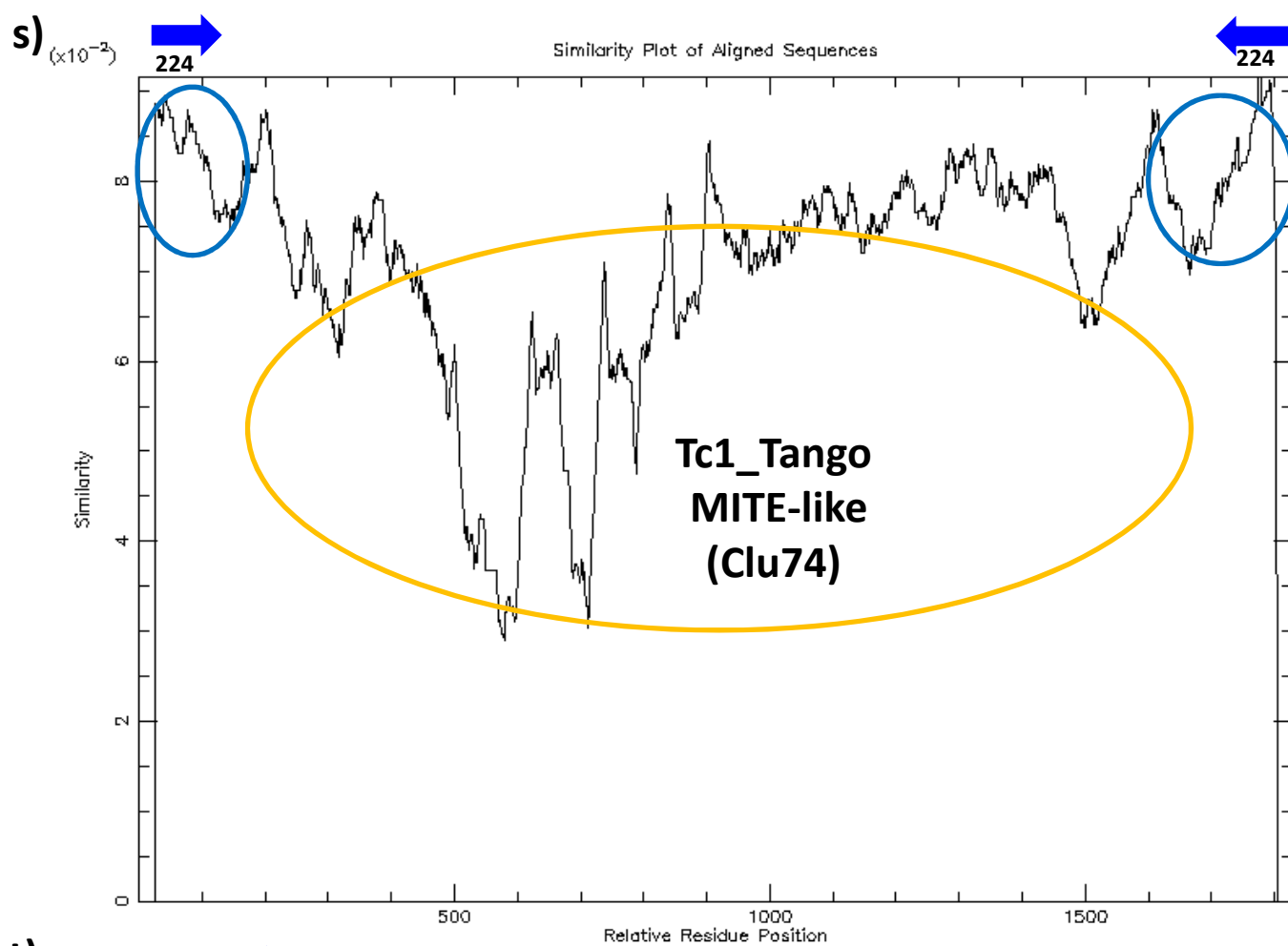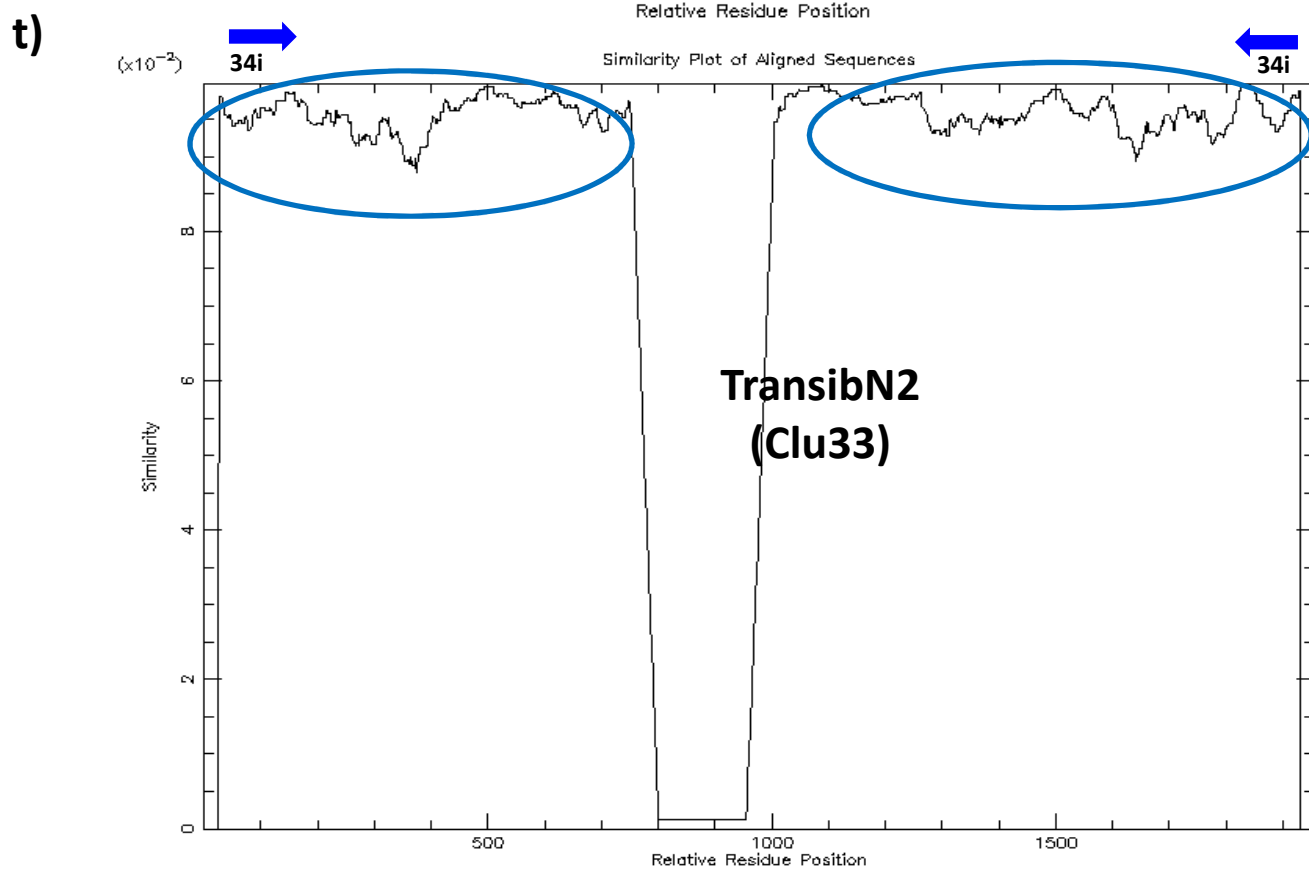

u)

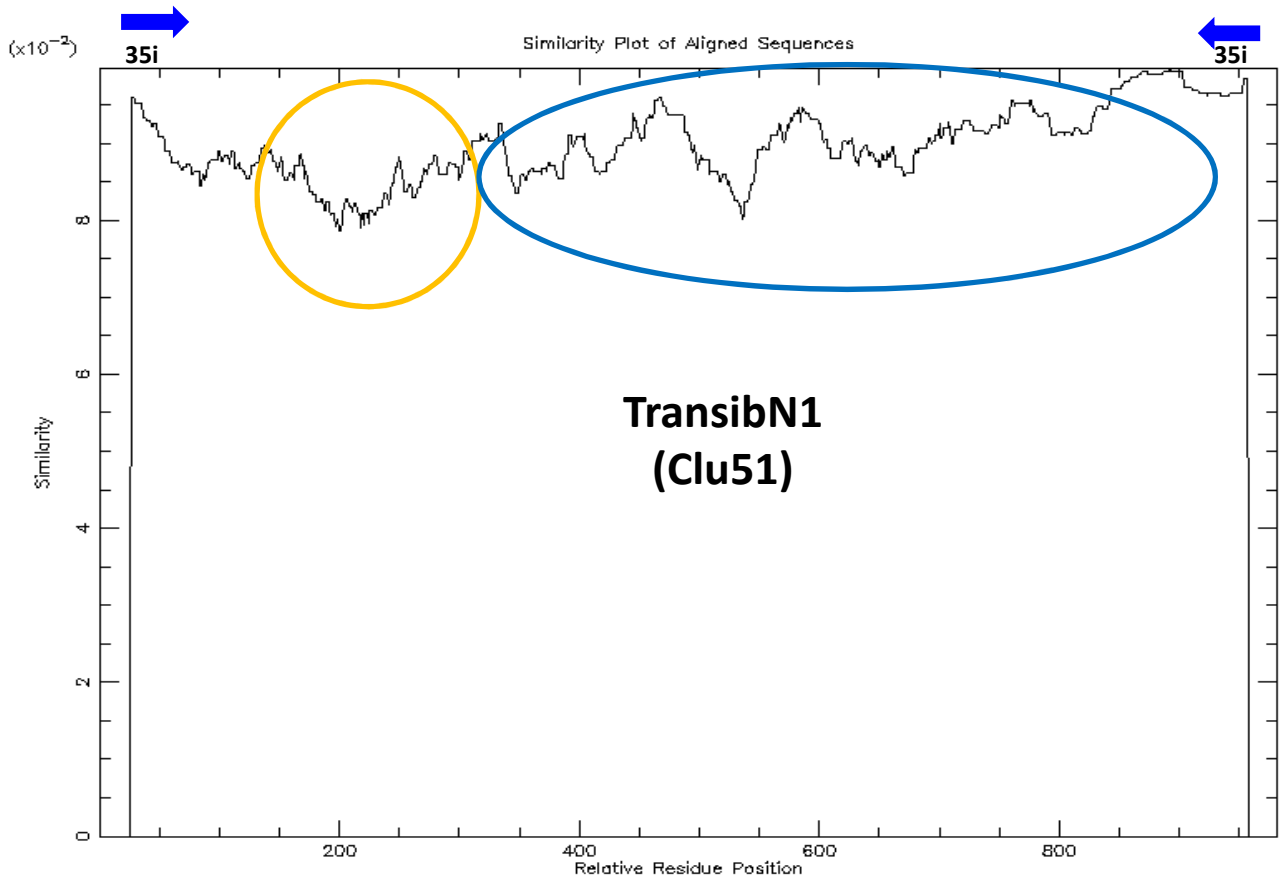

Supplement: Additional file 4 — Figure S3. Graphic representation of the deterioration profiles for the class II families analyzed in this study. See legend for Additional file 1: Figure S1 for detailed information. The blue arrows at the top of each graph represent the position and length of the TIRs in the canonical full-length element. The red arrows indicate the actual TIRs in the MITE-like elements. An “I” after the number indicates incomplete TIRs. Note the different scales on the y-axis. (PDF 201 kb) [file 1471-2164-13-272-S4.pdf]
